# Supplementary material for: A fast and specific fluorescent probe for thioredoxin reductase that works via disulphide bond cleavage
Source: Nat Commun. 2019 Jun 21;10:2745. doi: 10.1038/s41467-019-10807-8 (PMC6588570; doi:10.1038/s41467-019-10807-8)
Supplement: Supplementary file 1 — Supplementary Information [file 41467_2019_10807_MOESM1_ESM.pdf]

Supplementary Information

A fast and specific fluorescent probe for thioredoxin reductase that works via disulphide bond cleavage

Li *et al.*

Contents

- Supplementary Figures 1-84 (Pages 3-58)
- Supplementary Tables 1-4 and Cartesian Coordinates (Pages 59-64)
- Supplementary Methods (Pages 65-75)
- Supplementary Notes (Pages 76-82)
- Supplementary References (Pages 83-85)

Supplementary Figures

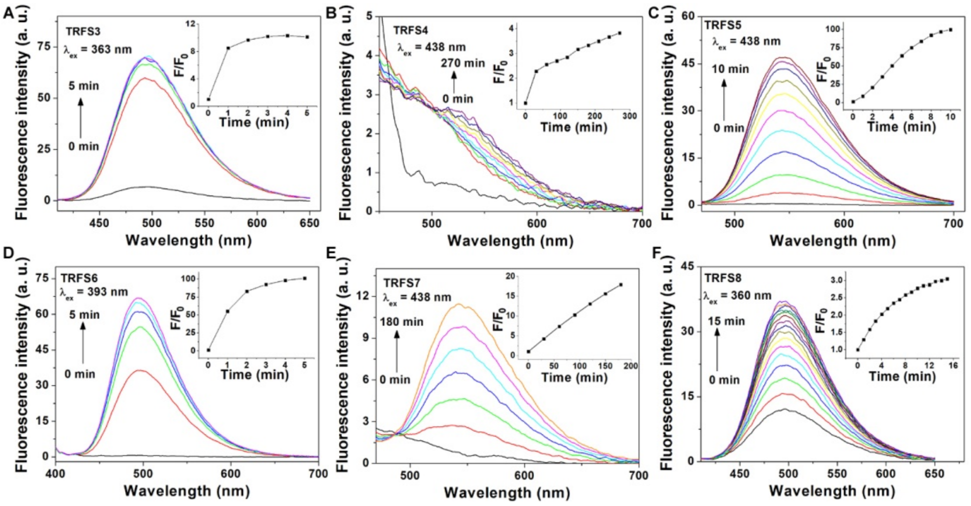

Supplementary Figure 1. Response of TRFS probes to TCEP. (A) λ_{ex}=363 nm; (B) λ_{ex}=438 nm; (C) λ_{ex}=438 nm; (D) λ_{ex}=393 nm; (E) λ_{ex}=438 nm; (F) λ_{ex}=360 nm. The insets in (A), (B), (C), (D), (E) and (F) showed the time-dependent changes of emission at 495 nm, 538 nm, 538 nm, 495 nm, 538 nm and 495 nm, respectively. The probes (10 μM) were incubated with TCEP (1 mM) at 37 ^{o}C in TE buffer. The time-dependent emission spectra were recorded. Source data are provided as a Source Data file.

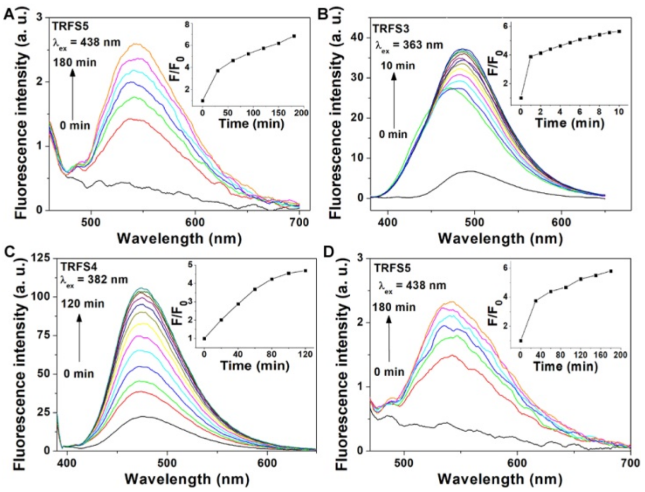

Supplementary Figure 2. Reduction of TRFS probes by GSH and TrxR/NADPH. (A) TRFS5 (10 μM) was incubated with GSH (1 mM) at 37 ^{o}C in TE buffer. The emission spectra (λ_{ex}=438 nm) were recorded every 30 min for 180 min. The inset showed the time-dependent changes of emission at 538 nm. (B)-(D) TRFS probes (10 μM) were incubated with TrxR/NADPH (50 nM/200 μM) at 37 ^{o}C in TE buffer. The emission spectra were recorded. (B) λ_{ex}=363 nm; (C) λ_{ex}=382 nm; (D) λ_{ex}=438 nm. The insets in (B), (C) and (D) showed the time-dependent changes of emission at 495 nm, 475 nm and 538 nm, respectively. Source data are provided as a Source Data file.

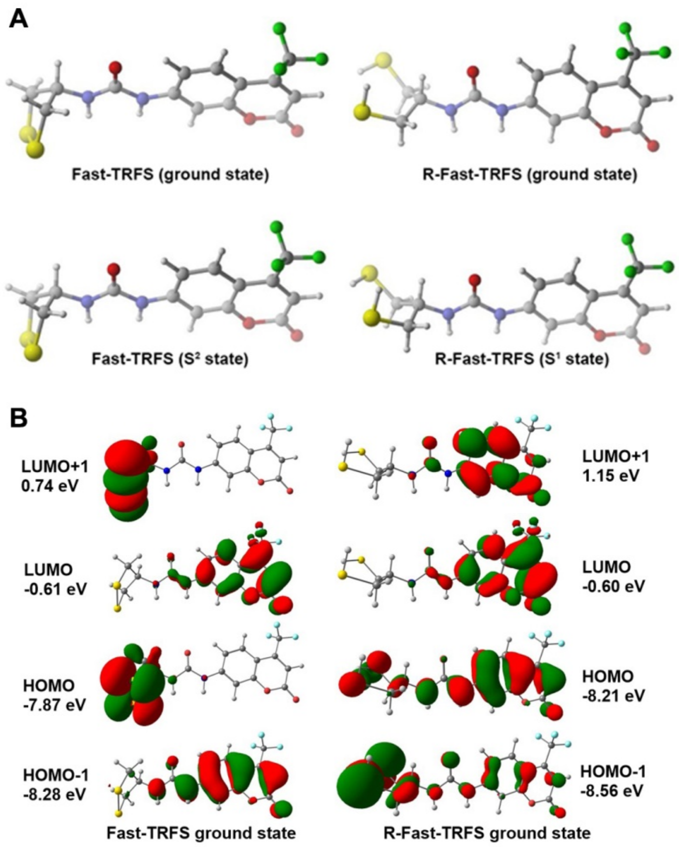

Supplementary Figure 3. Theoretical calculations of Fast-TRFS and the reduced Fast-TRFS. (A) Optimized structures of Fast-TRFS and R-Fast-TRFS. (B) Frontier molecular orbitals and corresponding energies of Fast-TRFS and R-Fast-TRFS in their ground states.

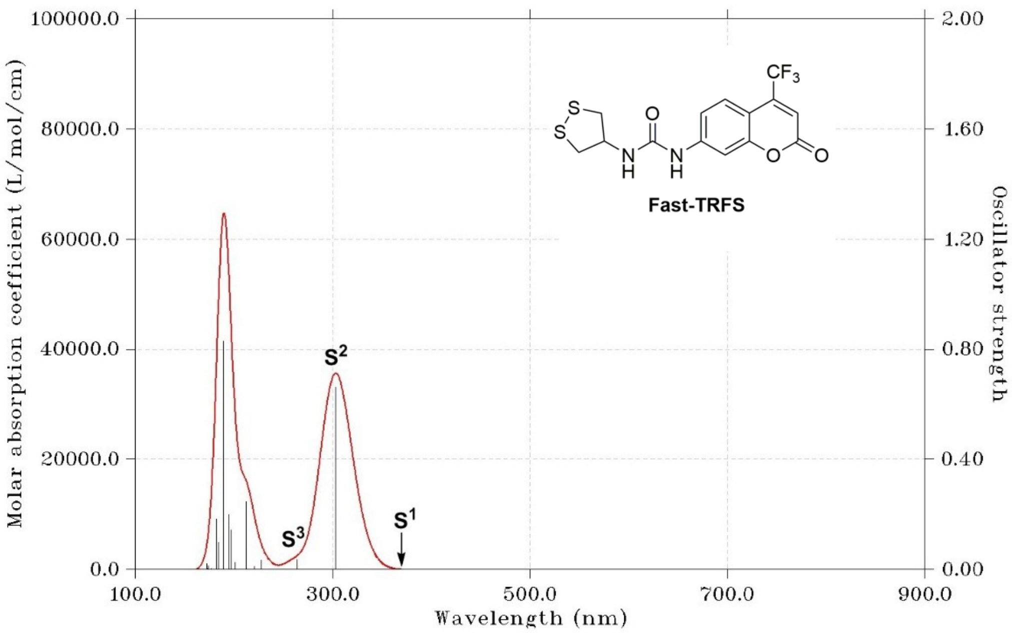

Supplementary Figure 4. The calculated UV-Vis absorption spectrum of Fast-TRFS using optimized ground state structure.

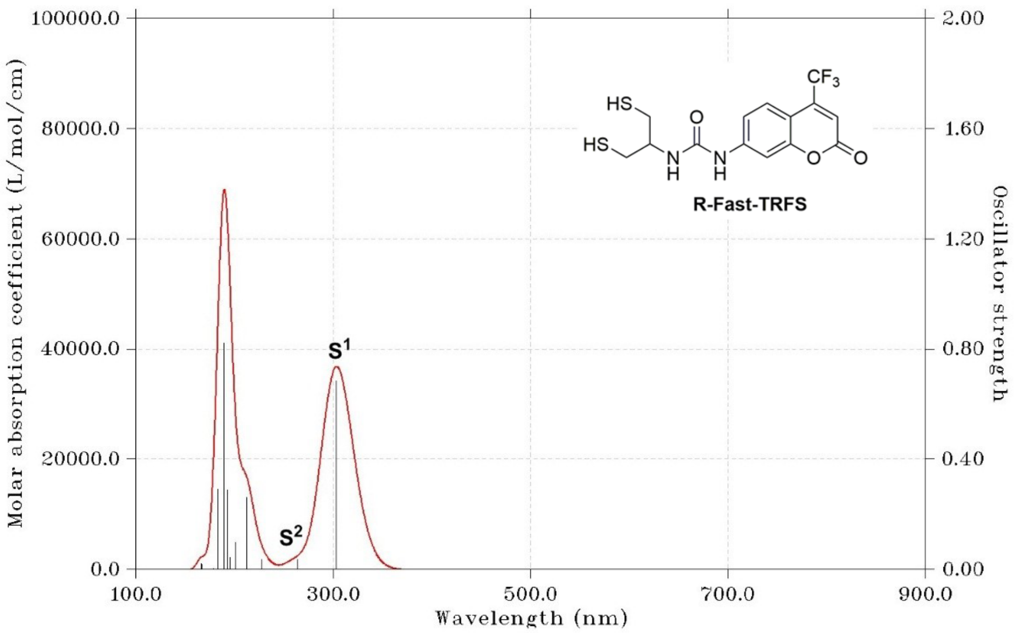

Supplementary Figure 5. The calculated UV-Vis absorption spectrum of R-Fast-TRFS using optimized ground state structure.

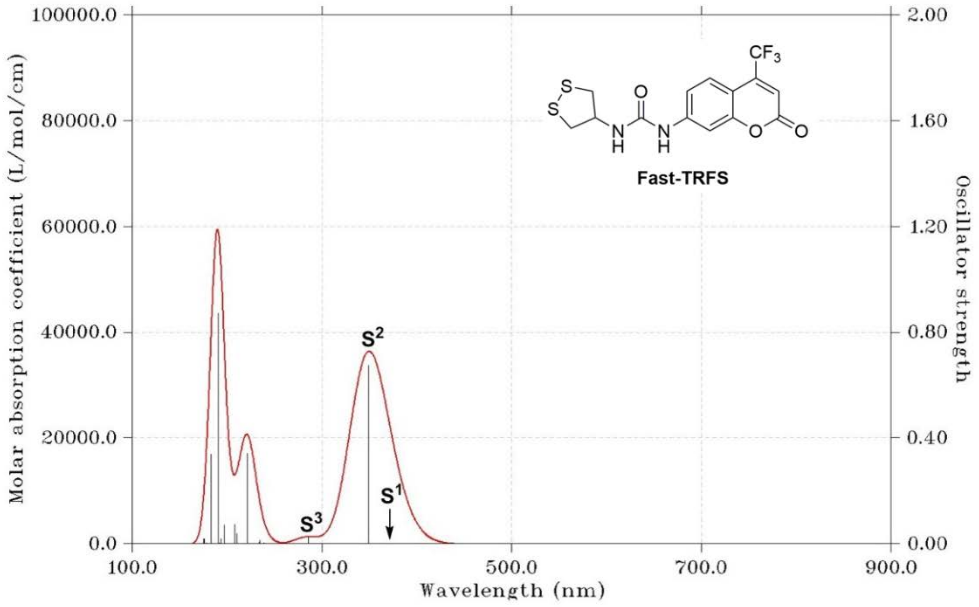

Supplementary Figure 6. The calculated fluorescence emission spectrum of Fast-TRFS using optimized $\mathbf{S^2}$ state structure.

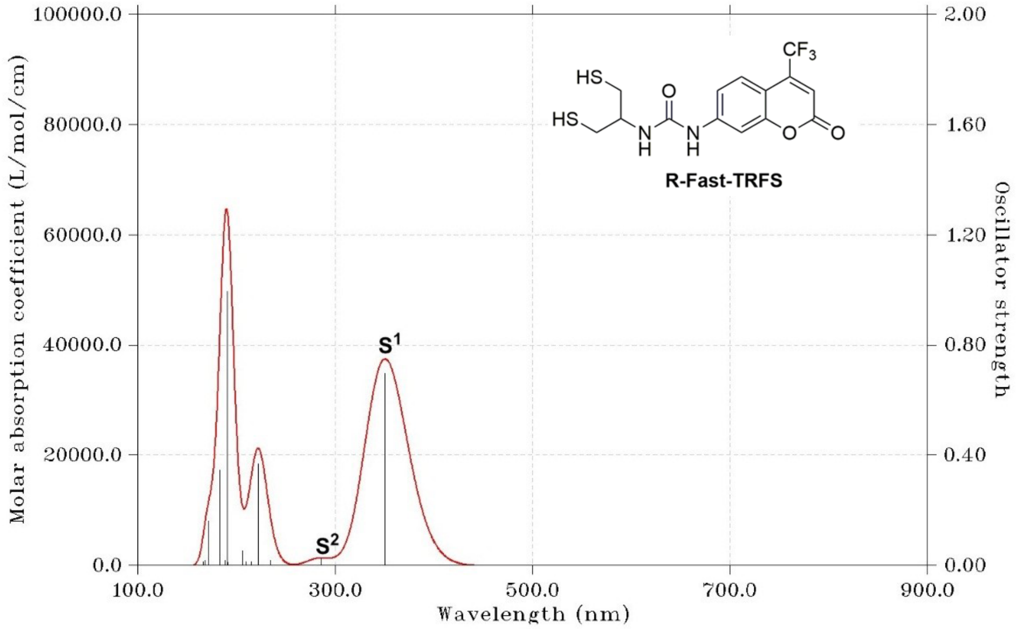

Supplementary Figure 7. The calculated fluorescence emission spectrum of R-Fast-TRFS using optimized $\mathbf{S^1}$ state structure.

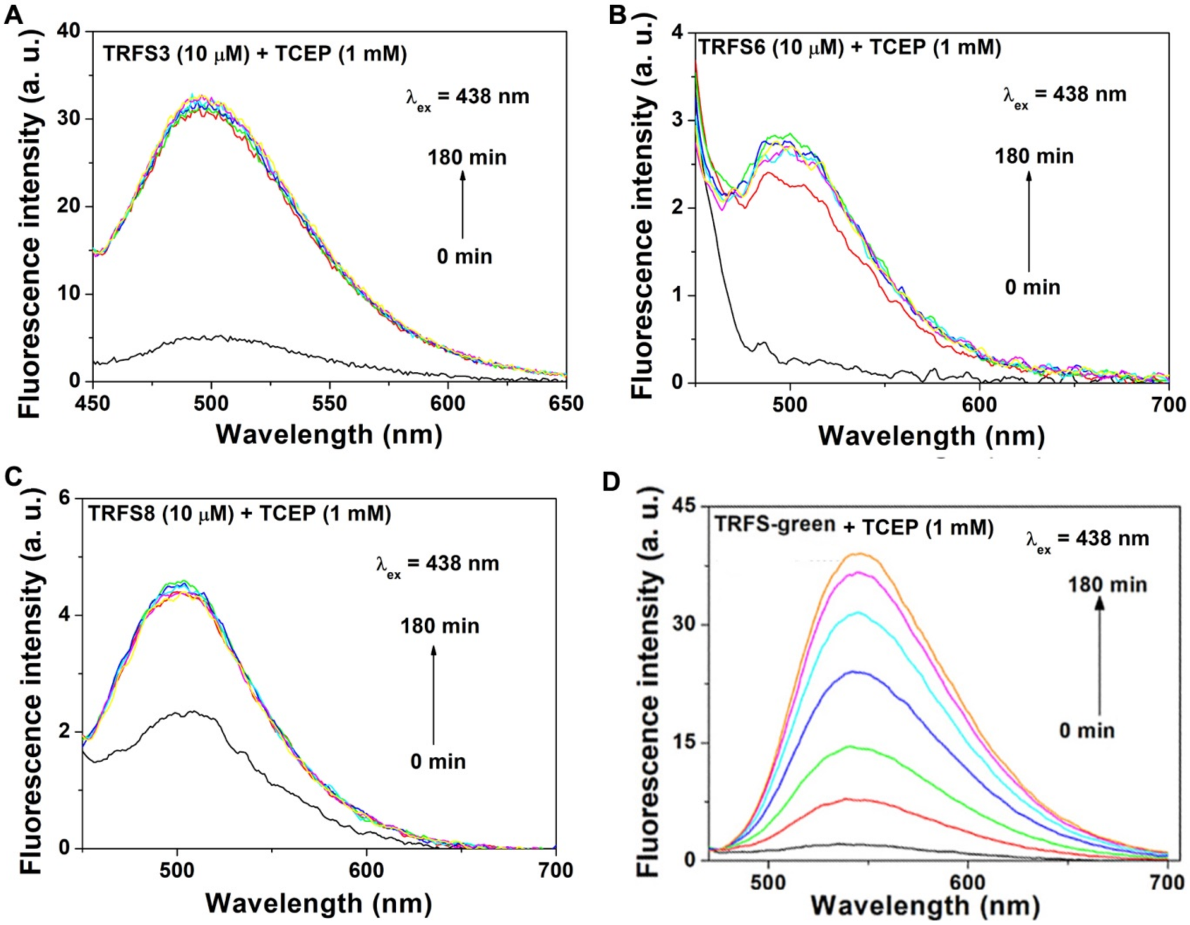

Supplementary Figure 8. Response of TRFS probes to TCEP. (A) TRFS3. (B) TRFS6. (C) TRFS8. The emission spectra were acquired by exciting the reaction mixture at 438 nm. The slight increase of the emission centering at aroud 495 nm is due to the direct clevage of the disulfide/diselenide bonds. For comparision, the realeased ANA from TRFS-green was shown in (D), which has a typical emission centering at 540 nm (D). Source data are provided as a Source Data file.

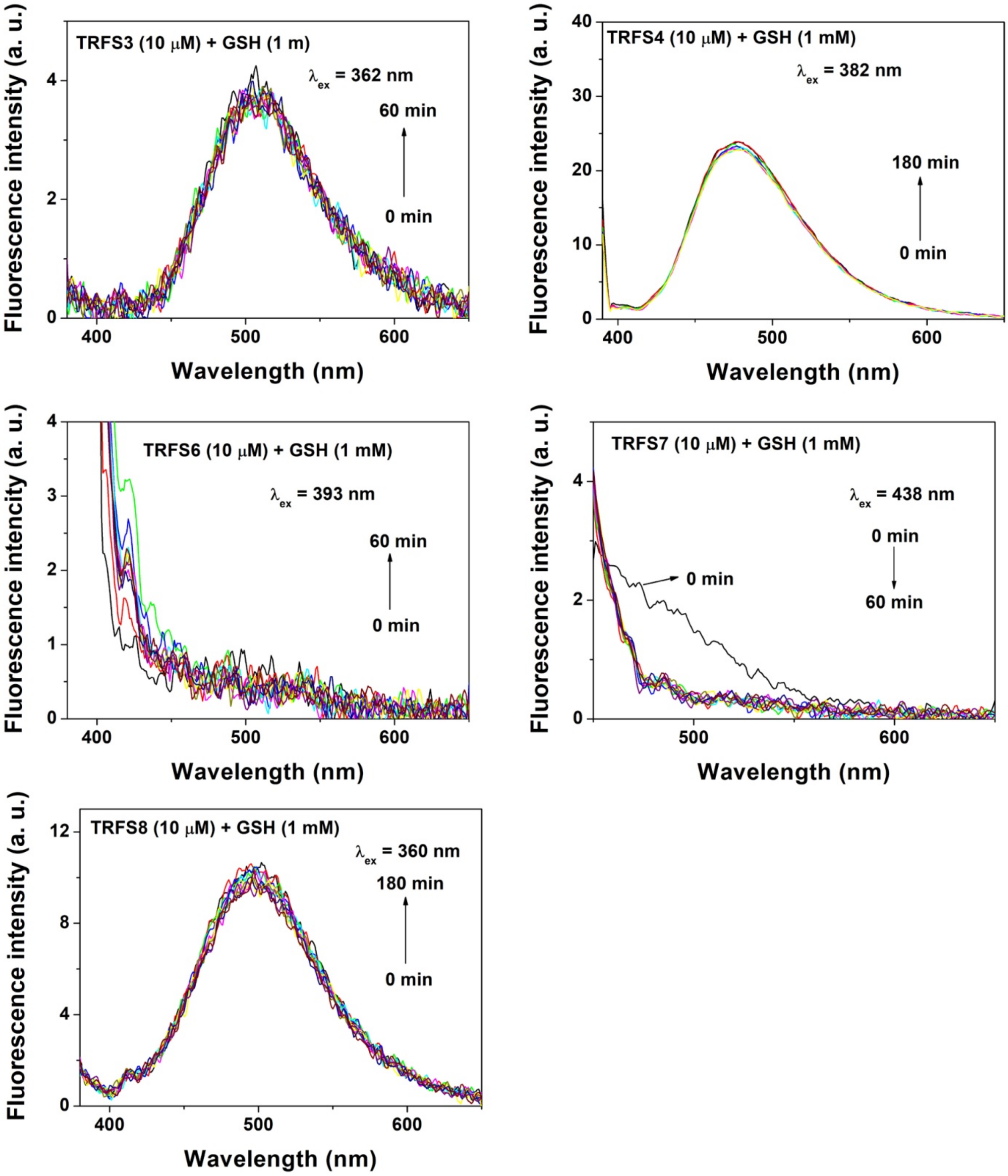

Supplementary Figure 9. Response of TRFS probes to GSH. Source data are provided as a Source Data file.

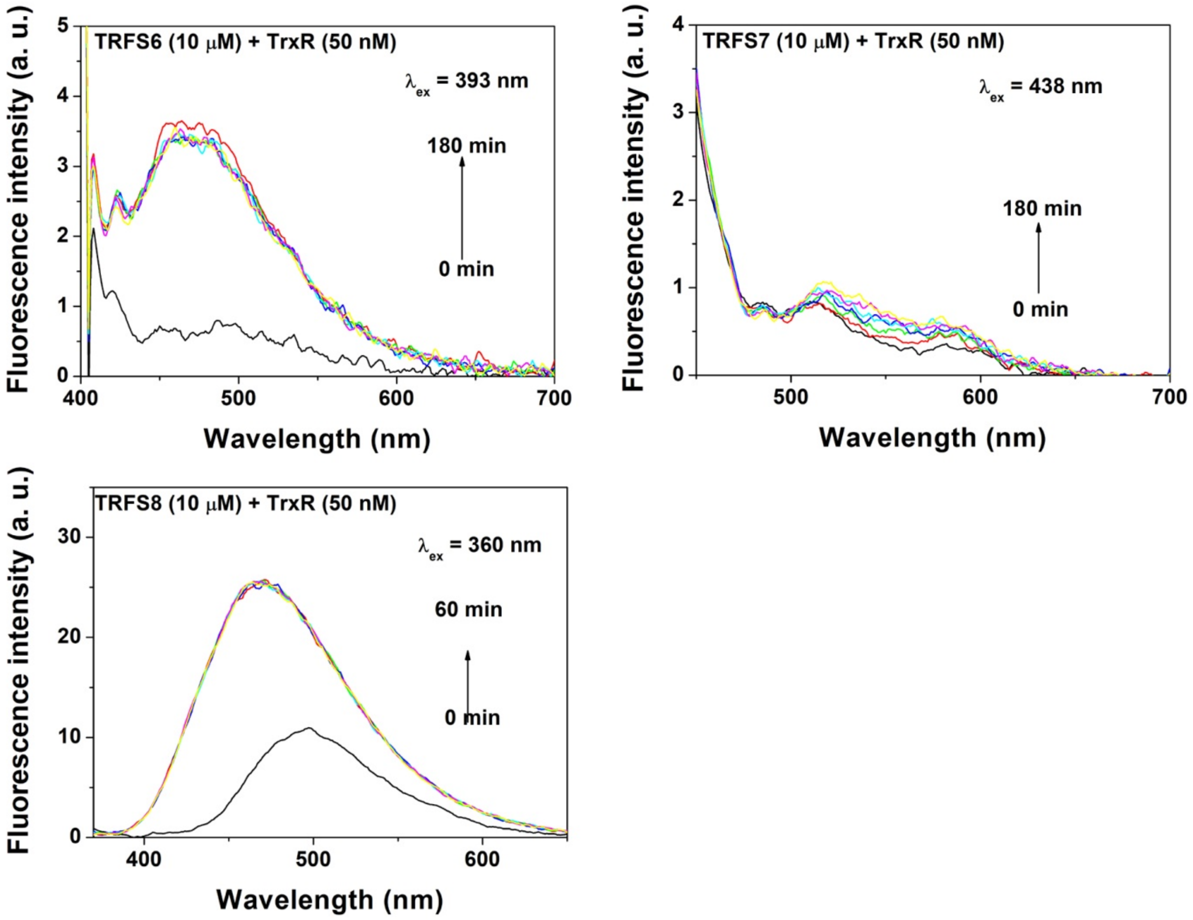

Supplementary Figure 10. Response of TRFS probes to TrxR/NADPH. The slight increase of the emission centering at aroud 475 nm when excited at 360 nm or 390 nm is due to the endogenous fluorescence of NADPH. Source data are provided as a Source Data file.

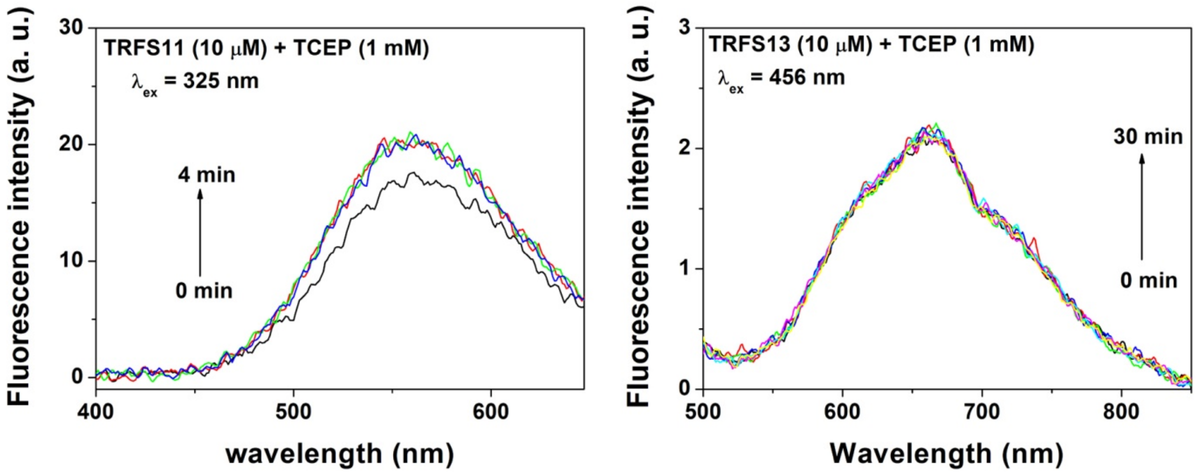

Supplementary Figure 11. Response of TRFS11 and TRFS13 to TCEP. Source data are provided as a Source Data file.

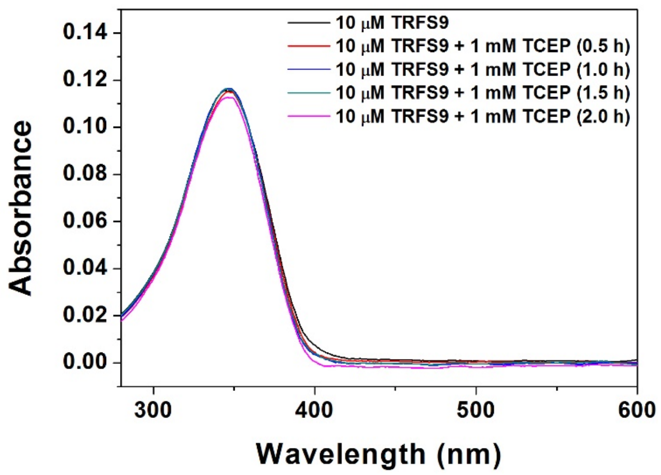

Supplementary Figure 12. No apparent absorbance change upon reduction of Fast-TRFS by TCEP. Fast-TRFS (10 μM) was incubated with TCEP (1 mM) at 37 °C in TE buffer. The absorbance spectra were scanned every 0.5 h for 2 h. Source data are provided as a Source Data file.

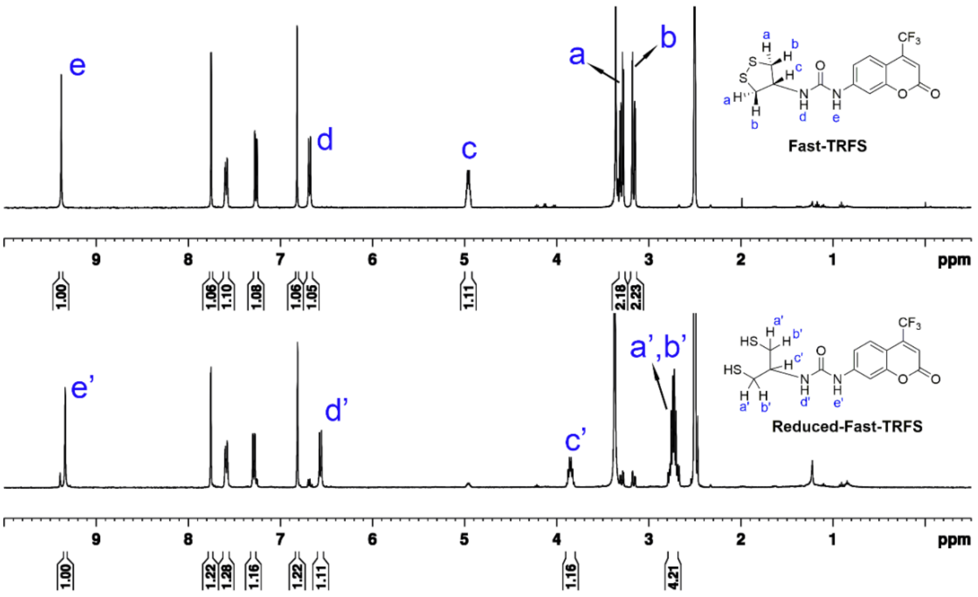

Supplementary Figure 13. ^{1}H NMR spectra of Fast-TRFS and R-Fast-TRFS in DMSO-d_6 (400 MHz). Source data are provided as a Source Data file.

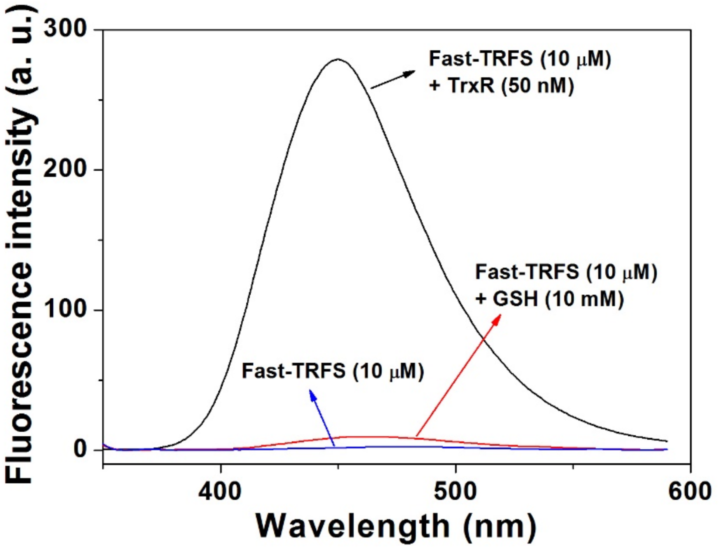

Supplementary Figure 14. Response of Fast-TRFS to GSH or TrxR. Fast-TRFS (10 μM) was incubated with GSH (10 mM) or TrxR (50 nM)/NADPH (200 μM) at 37 ^{o}C in TE buffer for 30 min. Source data are provided as a Source Data file.

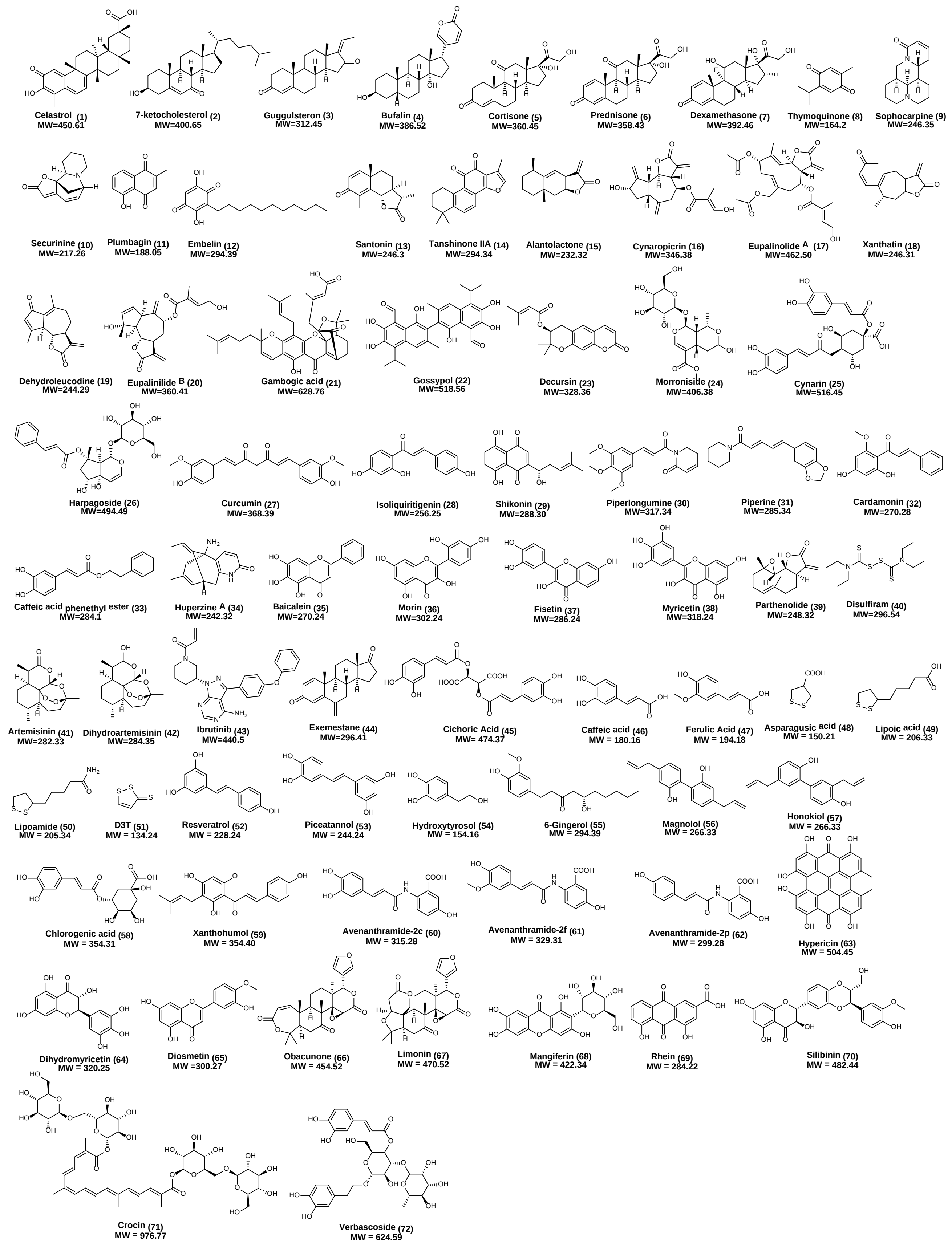

Supplementary Figure 15. Information of the natural product library.

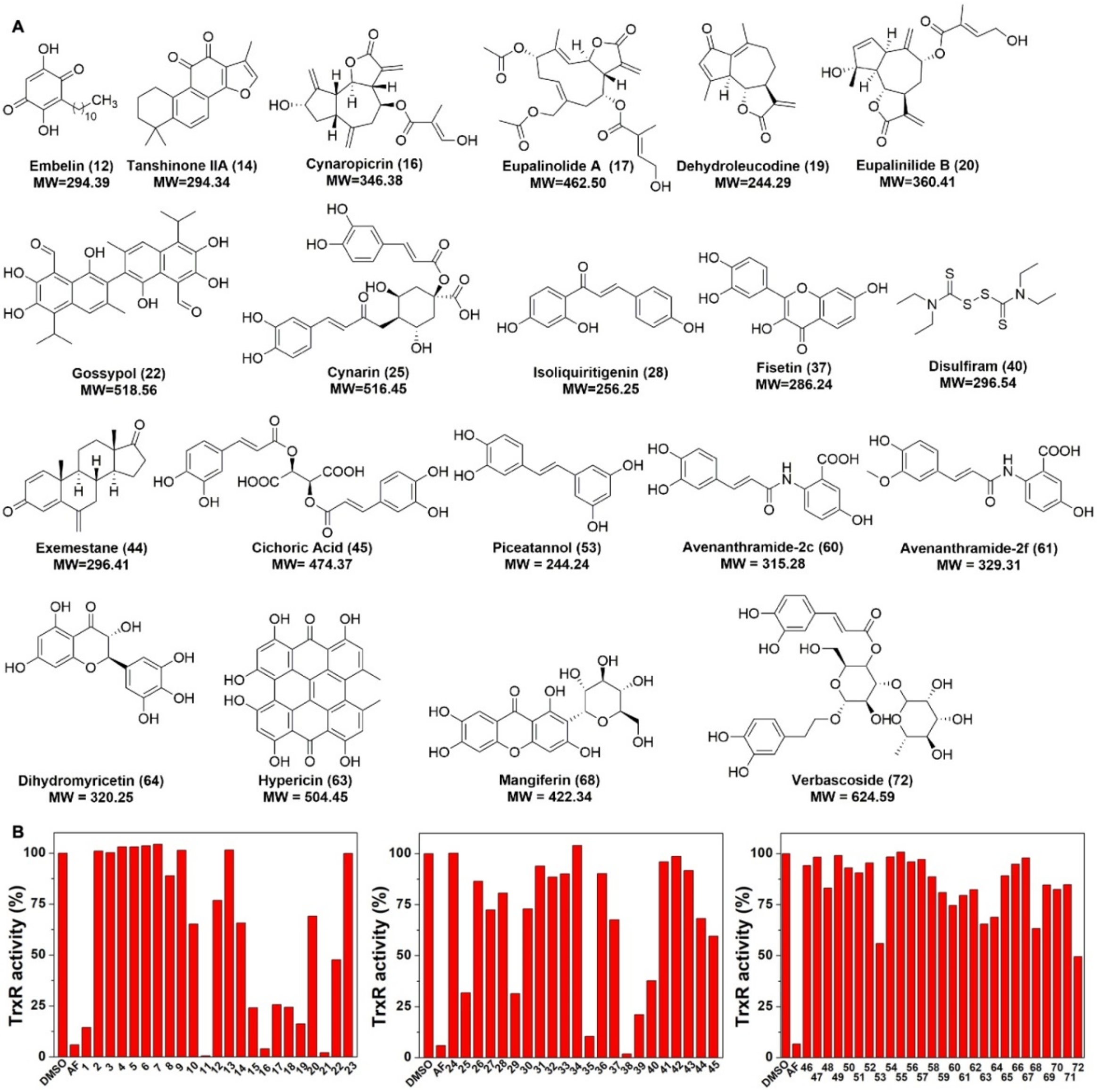

Supplementary Figure 16. Screening of natural product library. (A) Natural product inhibitors of TrxR discovered by the Fast-TRFS-based screening assay. (B) Screening TrxR inhibitors by Fast-TRFS using the crude protein extract as the source of TrxR. The HeLa cell lysate (0.3 mg/mL) was incubated with NADPH (100 μM) for 5 min at 37 ^{o}C. Compounds (50 μM each) or AF (1 μM, postive control) were then added and the mixture were continued to incubate for 1 h. The probe Fast-TRFS (10 μM) and NADPH (100 μM) were added to initiate the enzymatic reduction of Fast-TRFS. As the compounds were dissolved in DMSO, the blank sample was introduced the same ammount of DMSO (0.1%). The fluorescence change at 460 nm was recorded (λ_{ex}=345 nm) for 10 min on a fluorescent plate reader (Tecan Infinite M200), and the rate of fluorescence increase within the initial 5 min was calculated. The relative TrxR activity was expressed as the percentage of the DMSO-treated smaple. Source data are provided as a Source Data file.

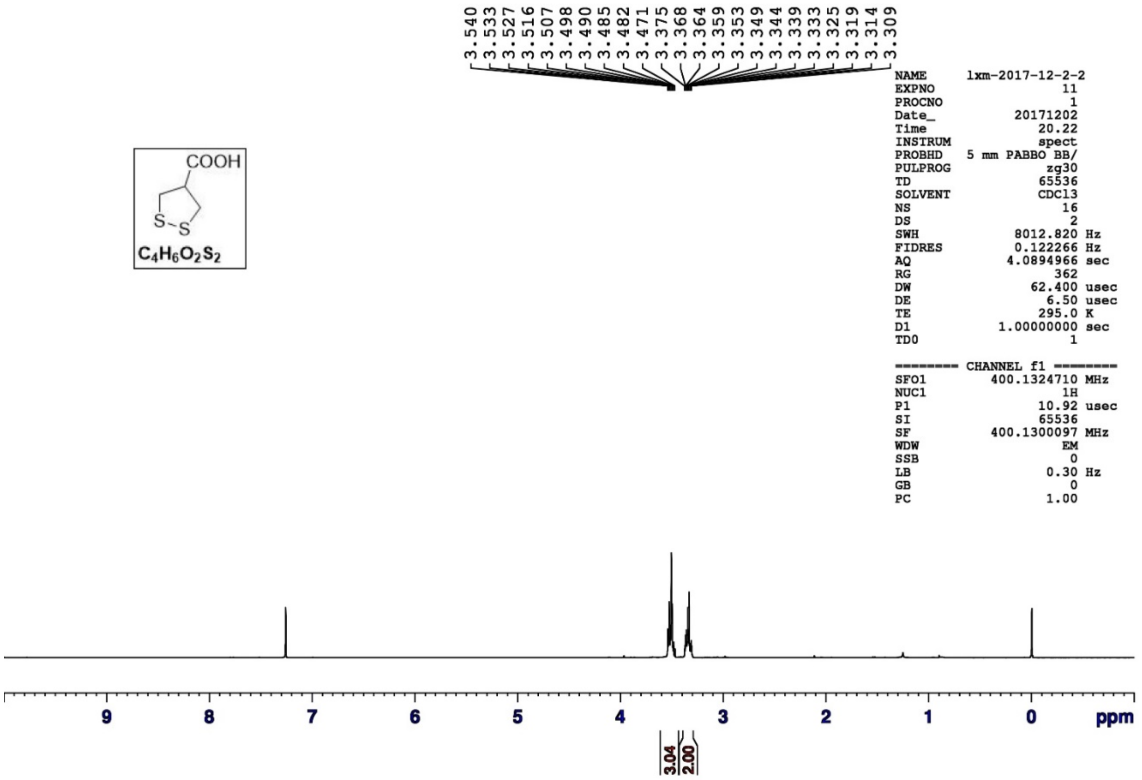

Supplementary Figure 17. ^{1}H NMR Spectra of compound **1** in $CDCl_3$ (400 MHz).

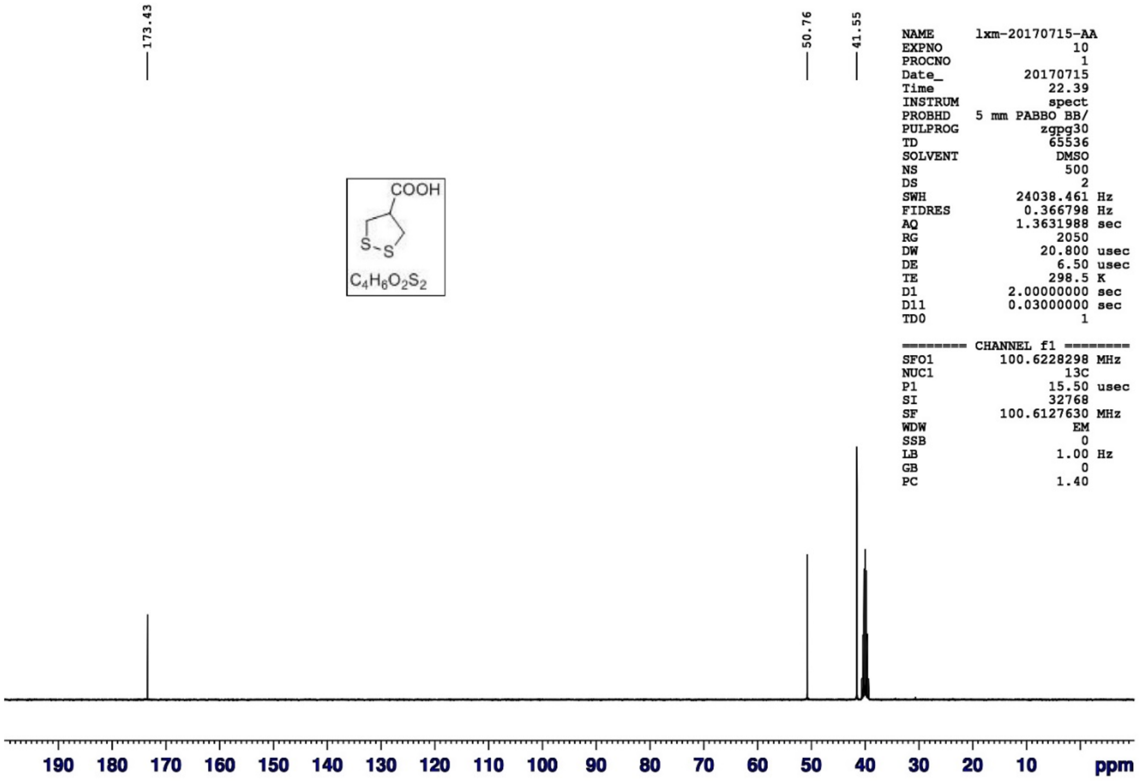

Supplementary Figure 18. ^{13}C NMR Spectra of compound **1** in DMSO-d_6 (100 MHz).

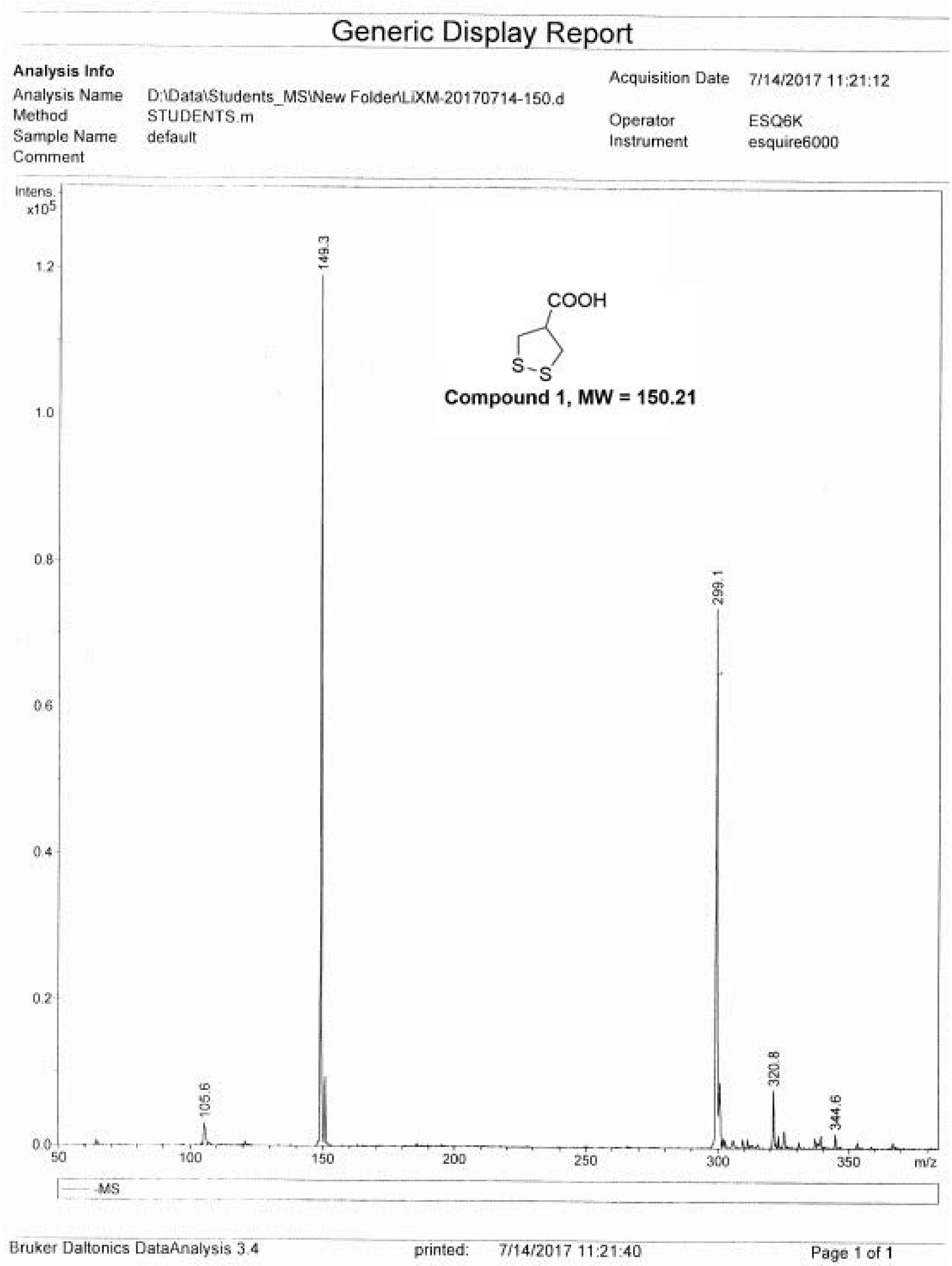

Supplementary Figure 19. MS Spectra of compound **1** (ESI).

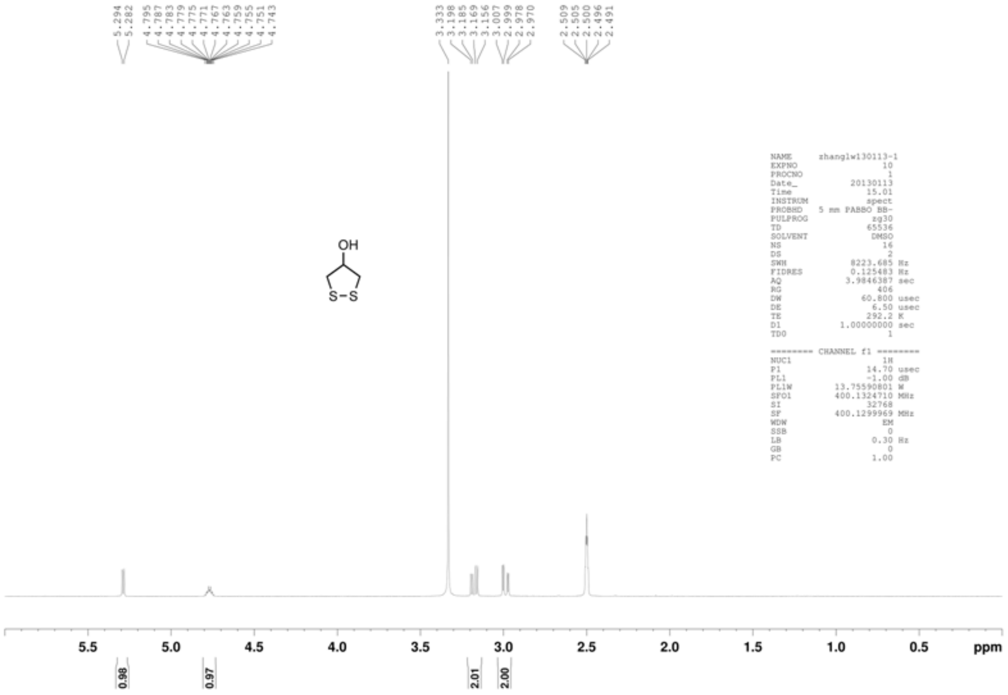

Supplementary Figure 20. ^{1}H NMR Spectra of compound **2** in DMSO-d_6 (400 MHz).

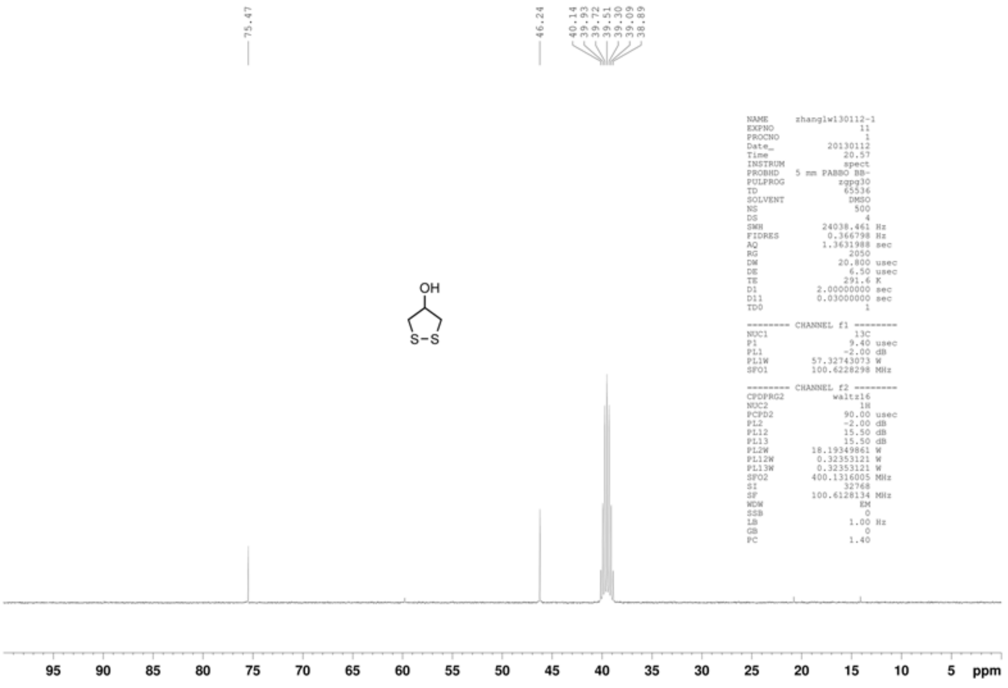

Supplementary Figure 21. ^{13}C NMR Spectra of compound **2** in DMSO-d_6 (100 MHz).

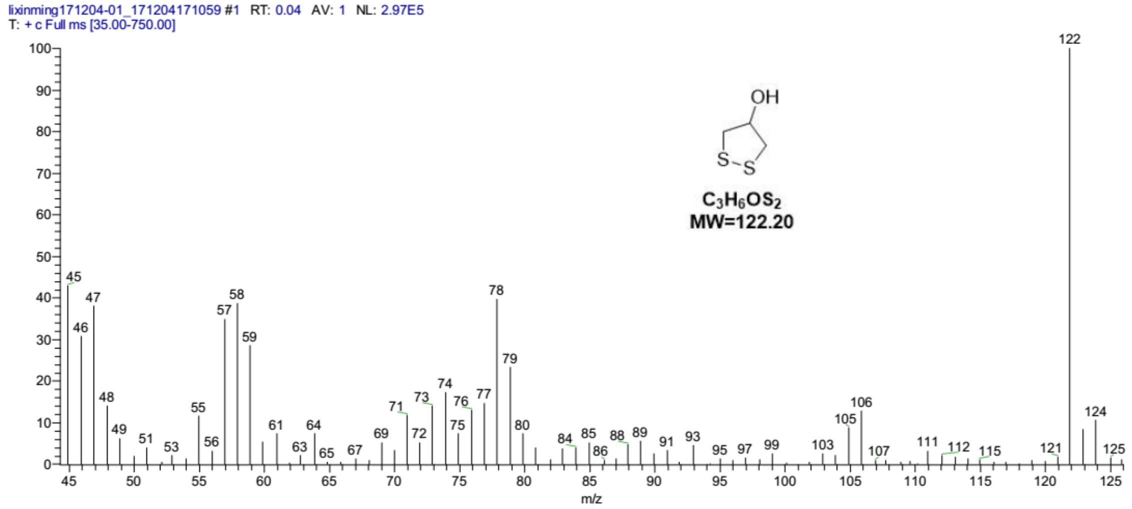

Supplementary Figure 22. MS Spectra of compound **2** (EI).

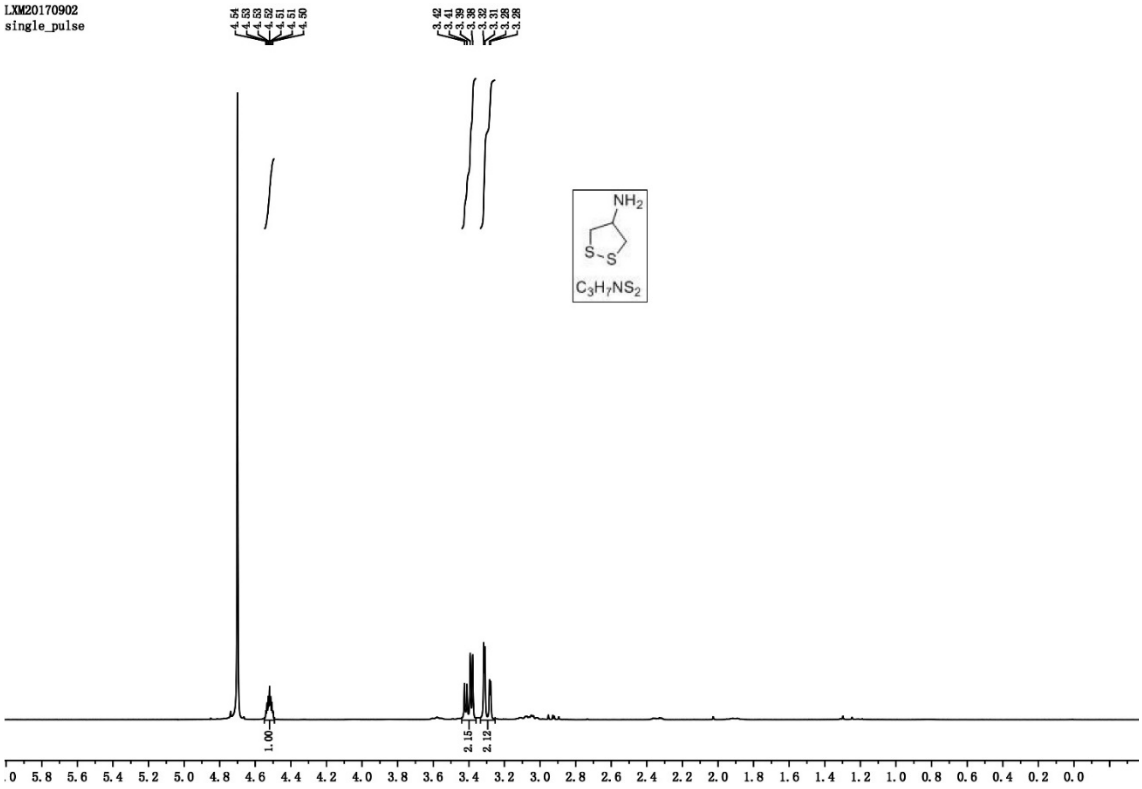

Supplementary Figure 23. ^{1}H NMR Spectra of compound **3** in D_2O (400 MHz).

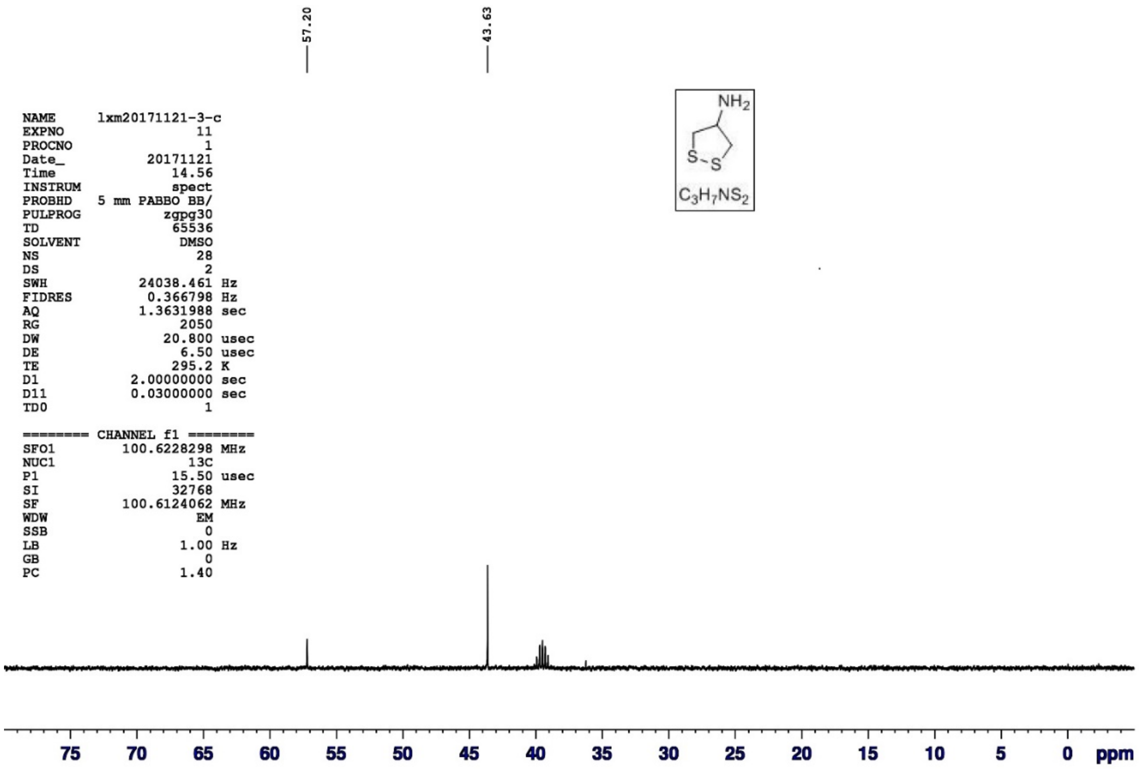

Supplementary Figure 24. ^{13}C NMR Spectra of compound **3** in DMSO-d_6 (100 MHz).

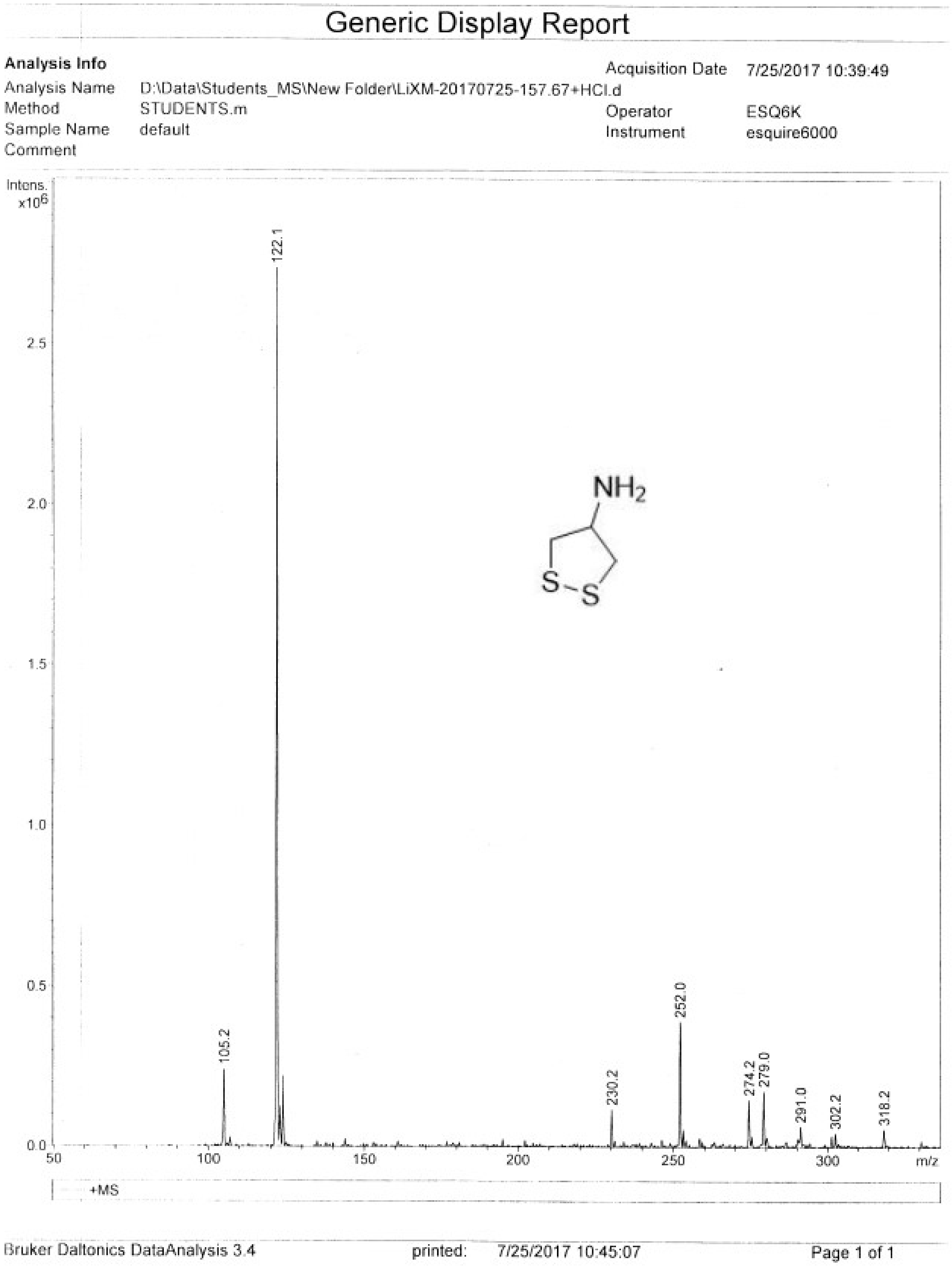

Supplementary Figure 25. MS Spectra of compound **3** (ESI).

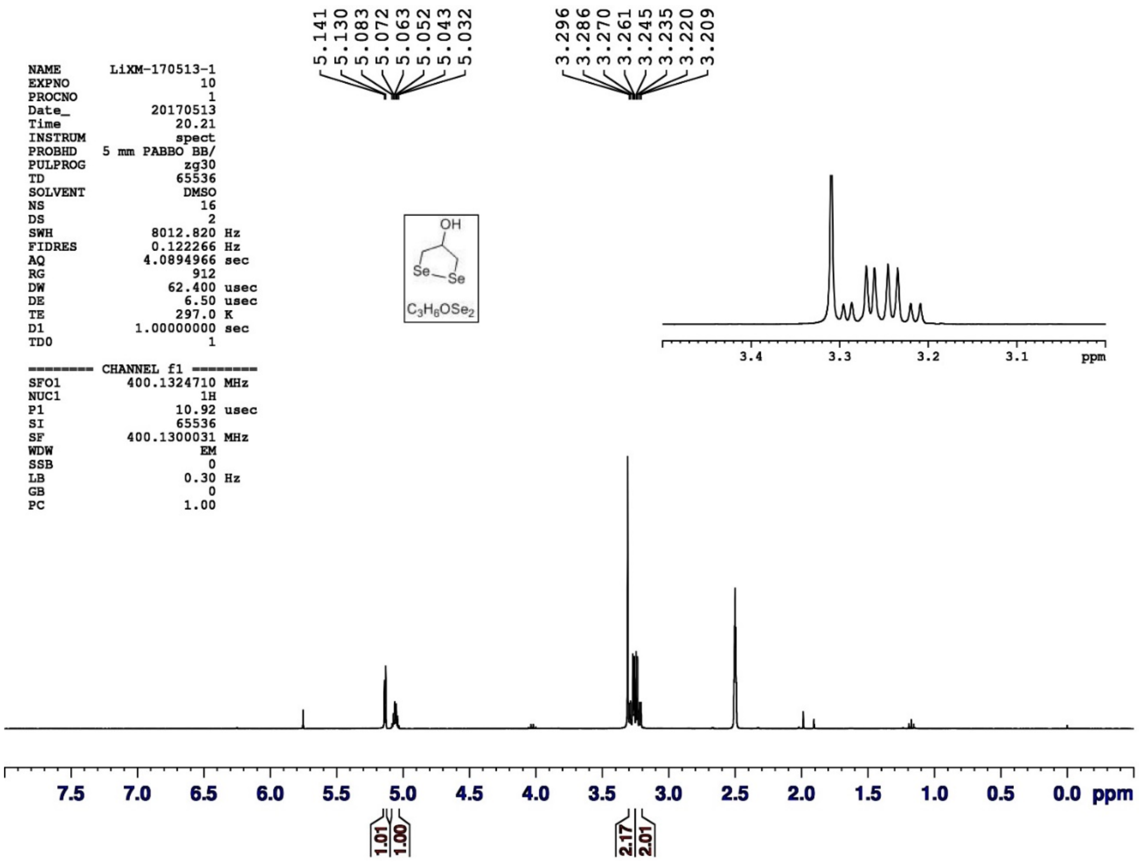

Supplementary Figure 26. ^{1}H NMR Spectra of compound **4** in DMSO-d_6 (400 MHz).

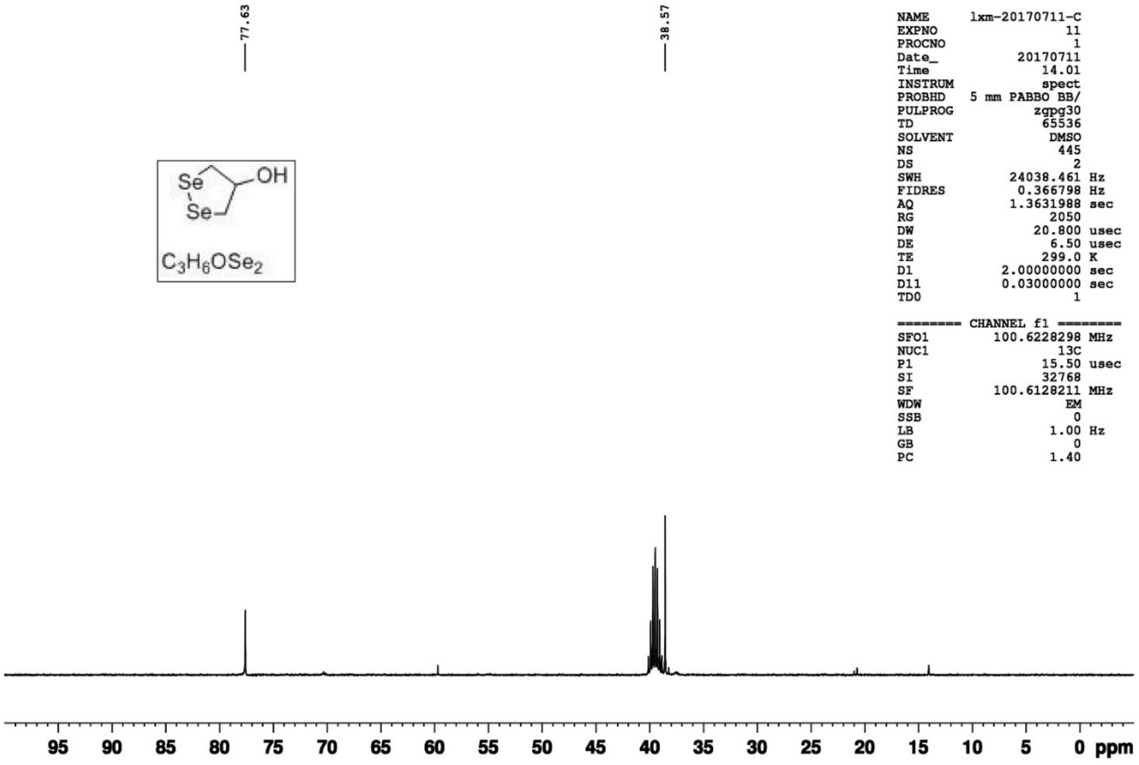

Supplementary Figure 27. ^{13}C NMR Spectra of compound **4** in DMSO-d_6 (100 MHz).

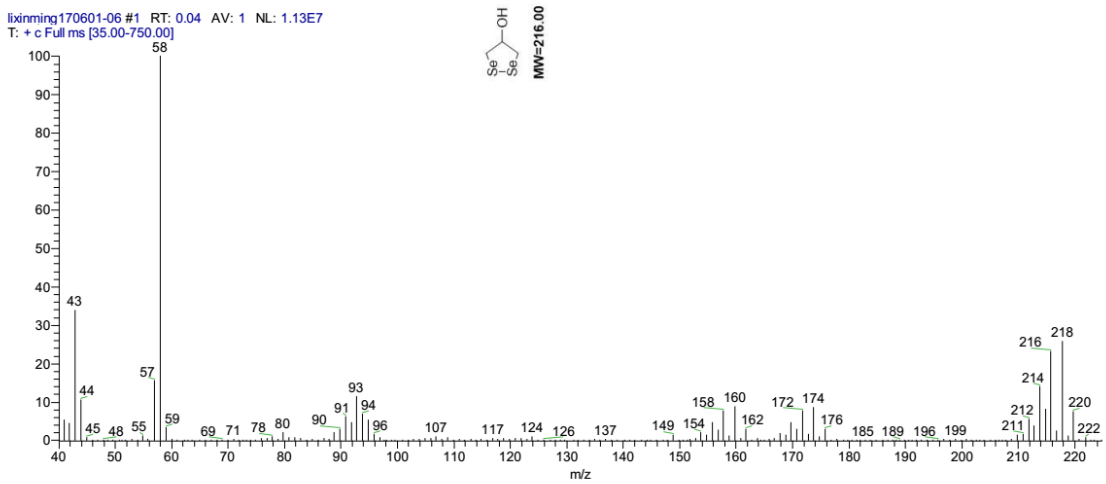

Supplementary Figure 28. MS Spectra of compound **4** (EI).

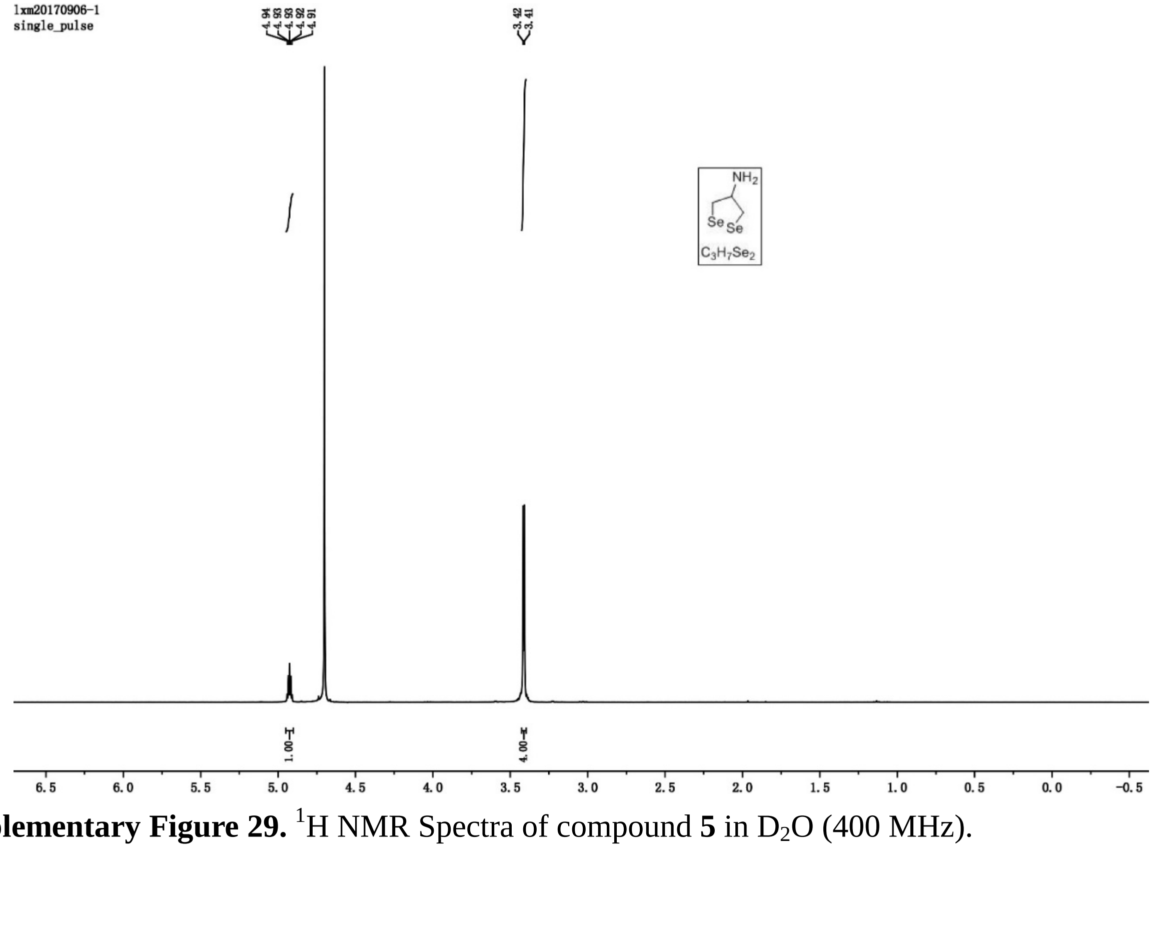

Supplementary Figure 29. ^{1}H NMR Spectra of compound **5** in D_2O (400 MHz).

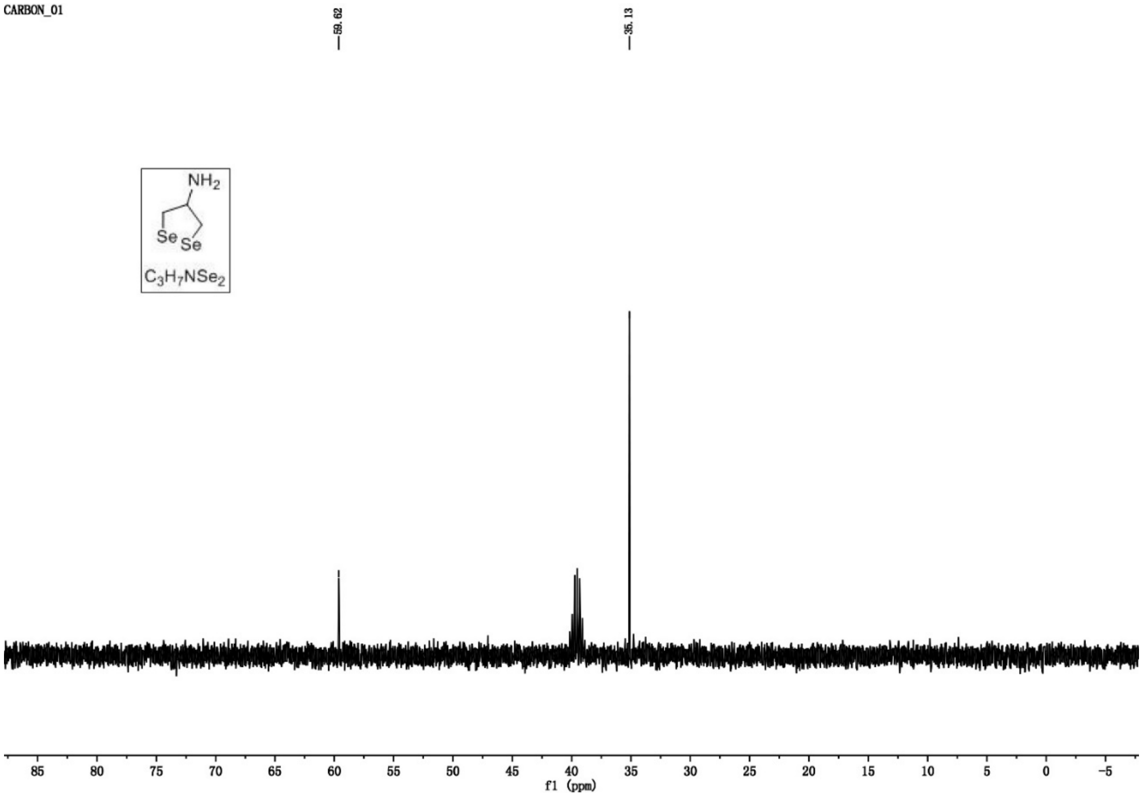

Supplementary Figure 30. ^{13}C NMR Spectra of compound **5** in DMSO-d_6 (100 MHz).

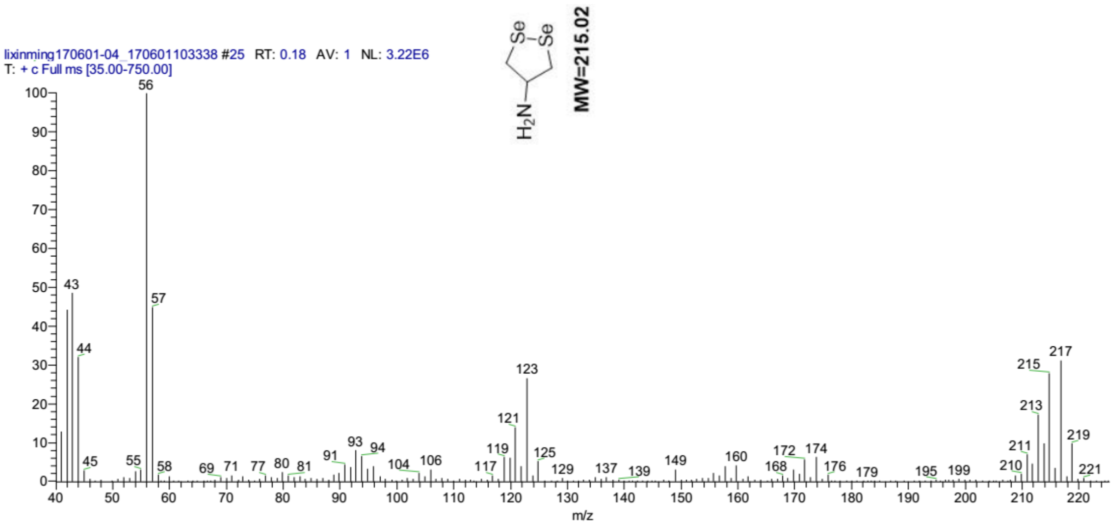

Supplementary Figure 31. MS Spectra of compound **5** (EI).

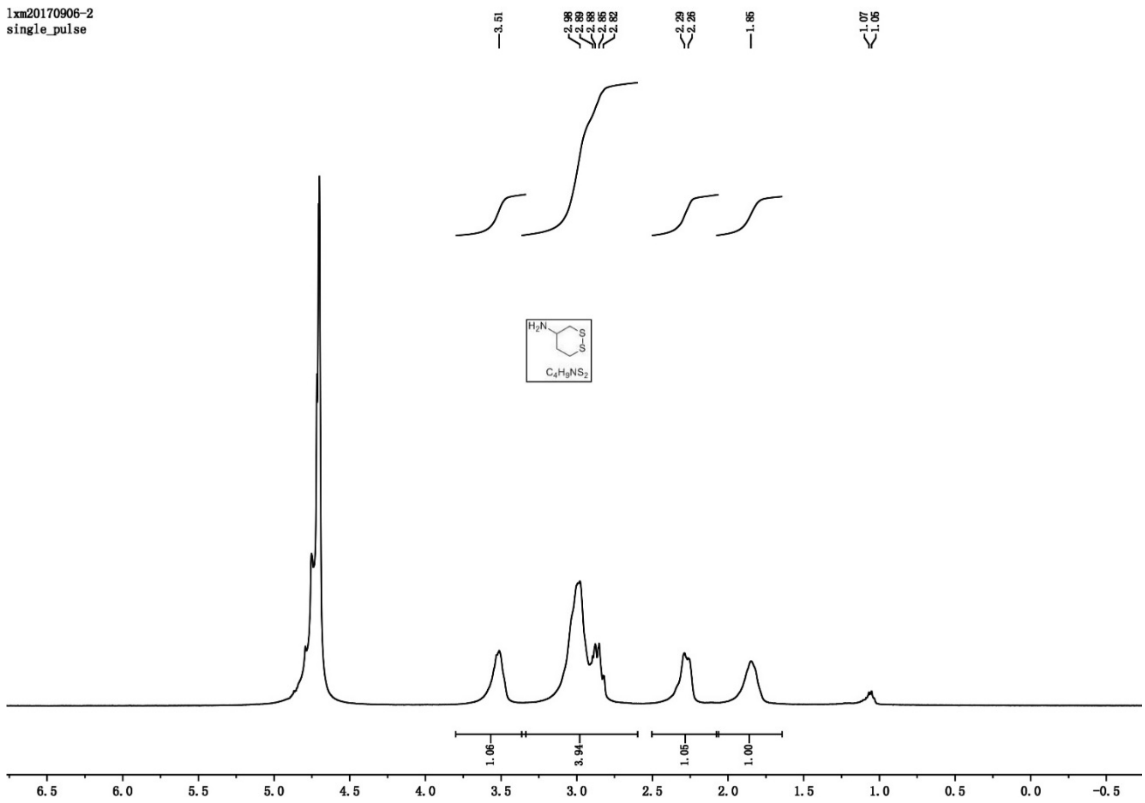

Supplementary Figure 32. ^{1}H NMR Spectra of compound **6** in D_2O (400 MHz).

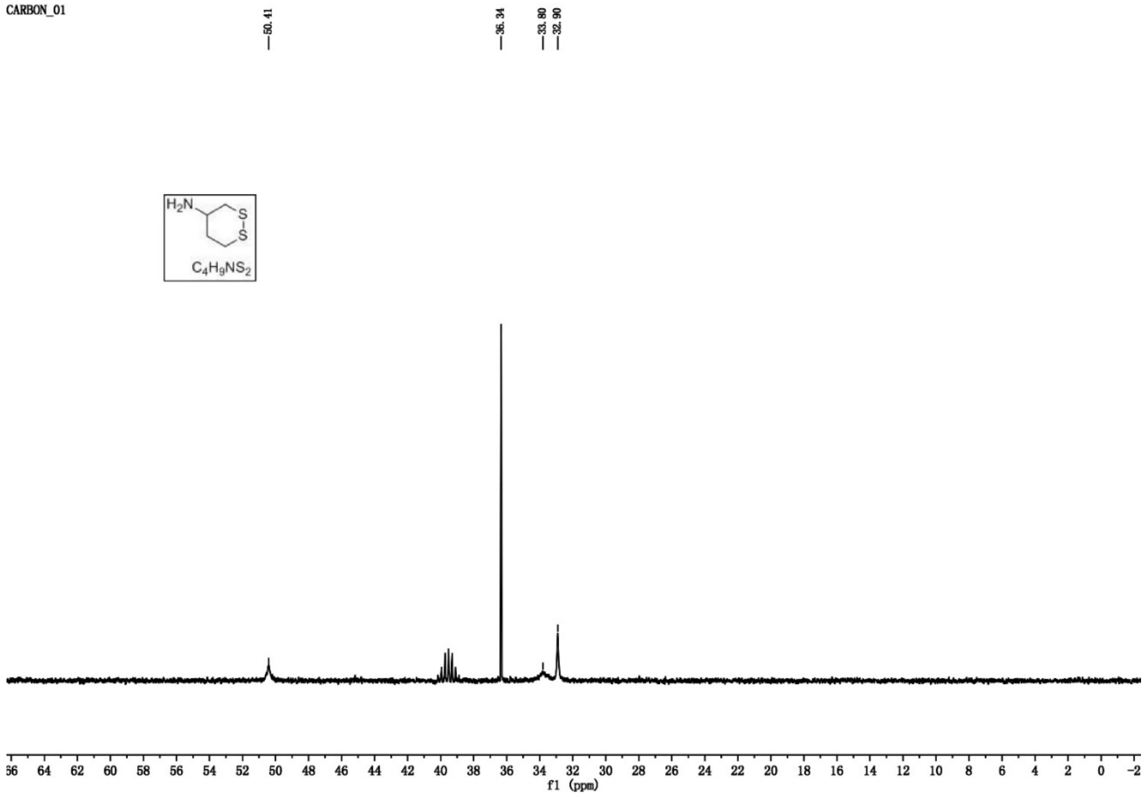

Supplementary Figure 33. ^{13}C NMR Spectra of compound **6** in DMSO-d_6 (100 MHz).

Generic Display Report

Analysis Info

Analysis Name	D:\Data\Students_MS\New Folder\LiXM-20170725-171.7-HCl.d	Acquisition Date	7/25/2017 10:48:22
Method	STUDENTS.m	Operator	ESQ6K
Sample Name	default	Instrument	esquire6000
Comment			

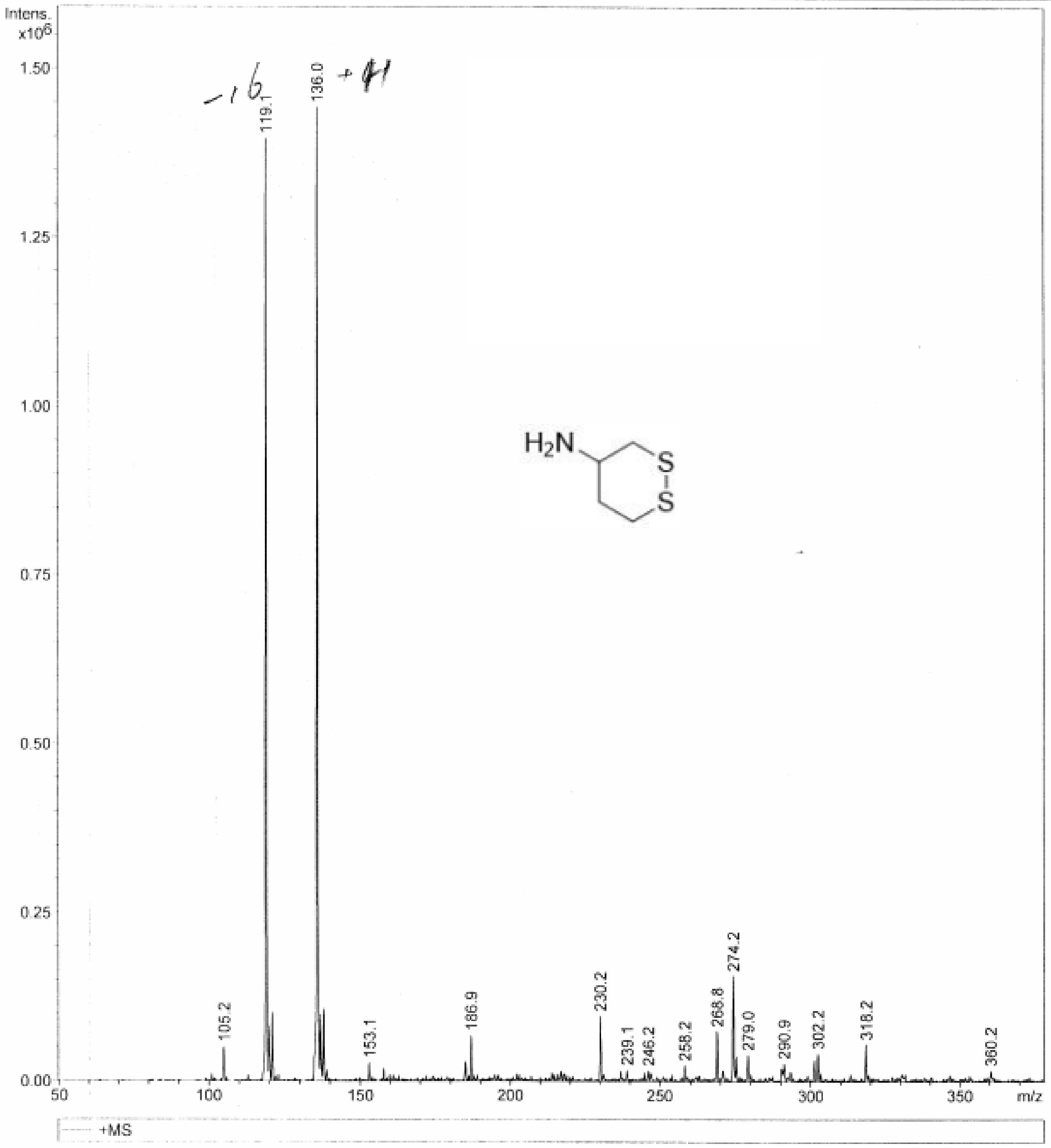

Bruker Daltonics DataAnalysis 3.4 printed: 7/25/2017 10:48:47 Page 1 of 1

Supplementary Figure 34. MS Spectra of compound **6** (ESI).

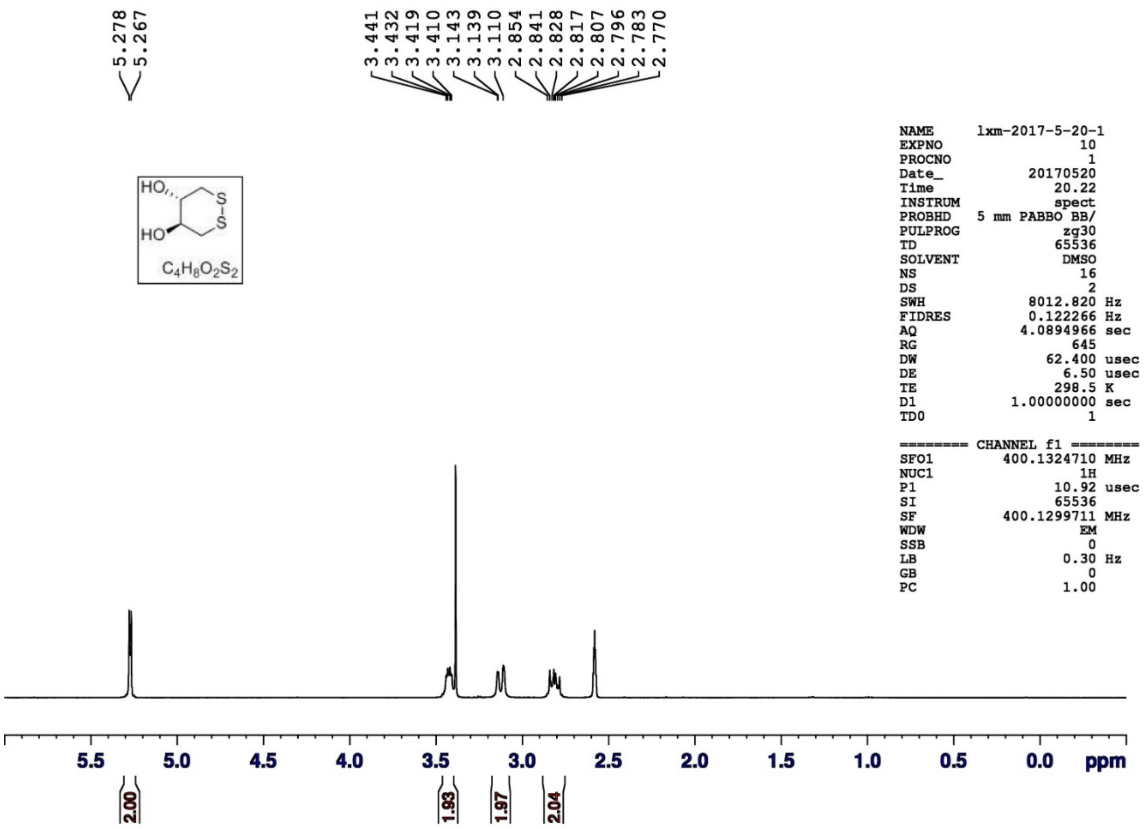

Supplementary Figure 35. ^{1}H NMR Spectra of compound **7** in DMSO-d_6 (400 MHz).

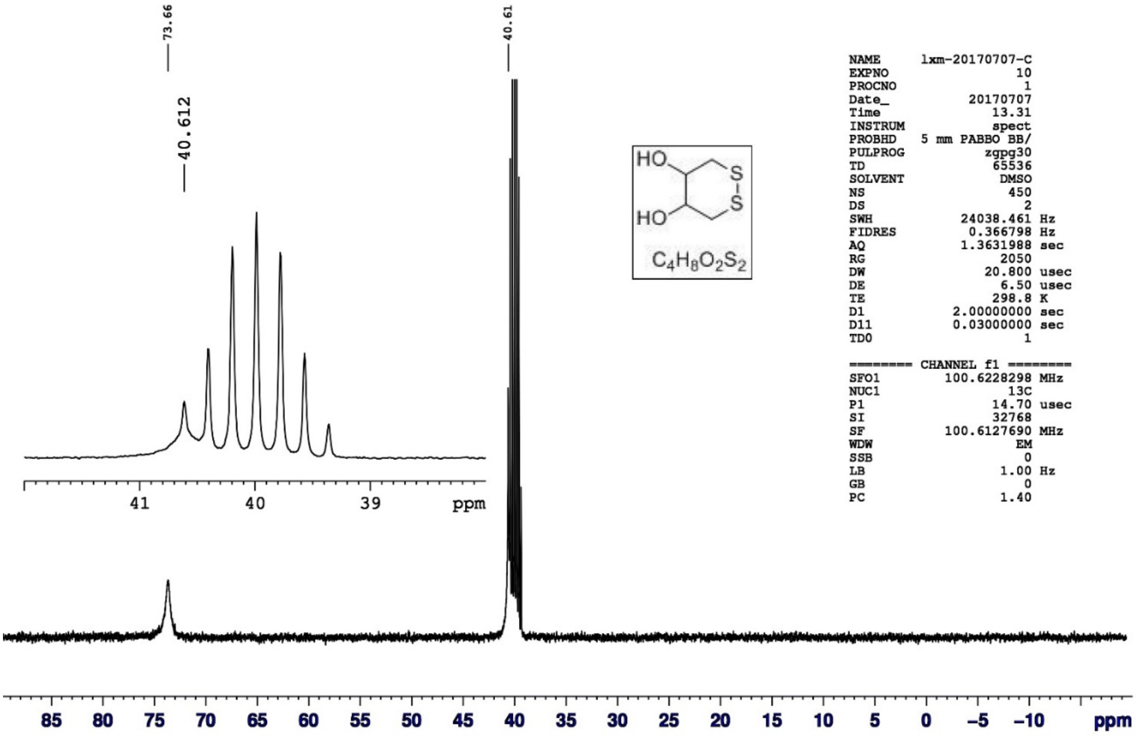

Supplementary Figure 36. ^{13}C NMR Spectra of compound **7** in DMSO-d_6 (100 MHz).

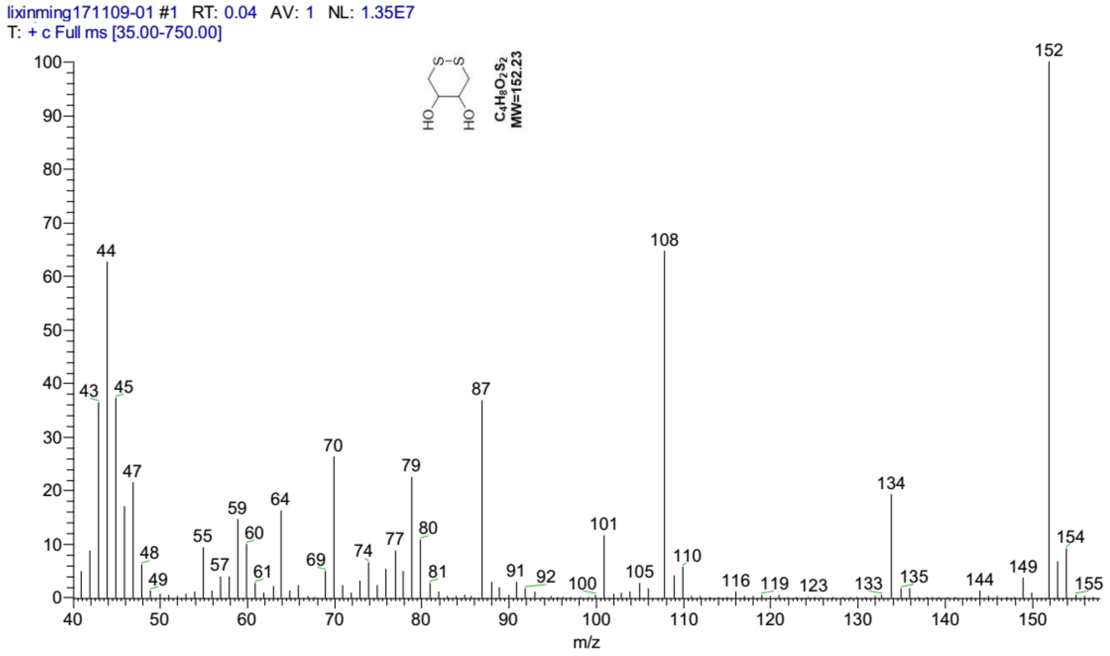

Supplementary Figure 37. MS Spectra of compound **7** (EI).

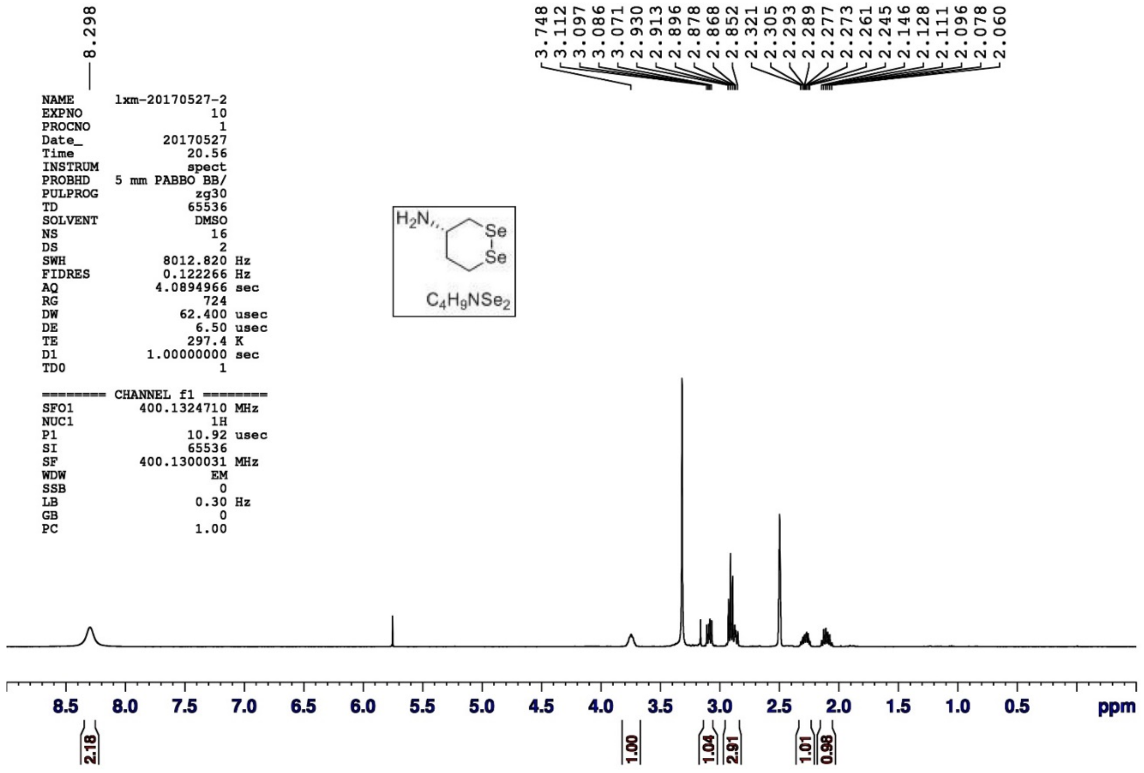

Supplementary Figure 38. ^{1}H NMR Spectra of compound **8** in DMSO-d_6 (400 MHz).

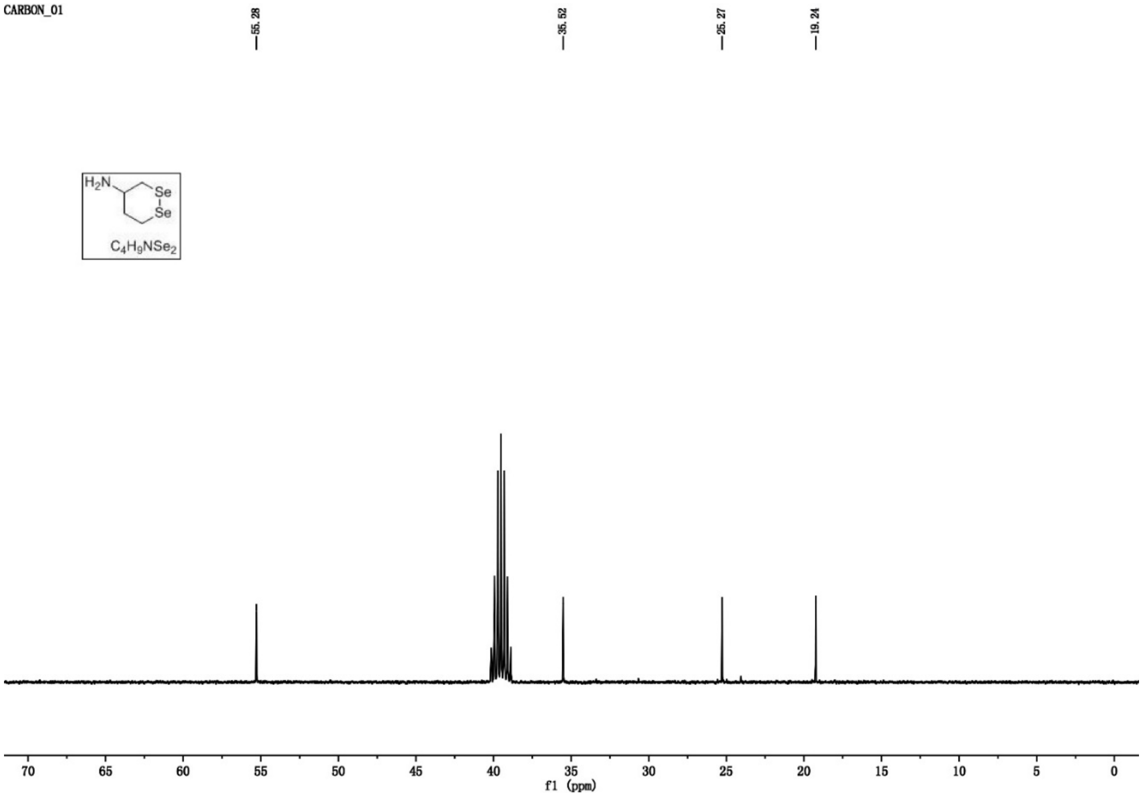

Supplementary Figure 39. ^{13}C NMR Spectra of compound **8** in DMSO-d_6 (100 MHz).

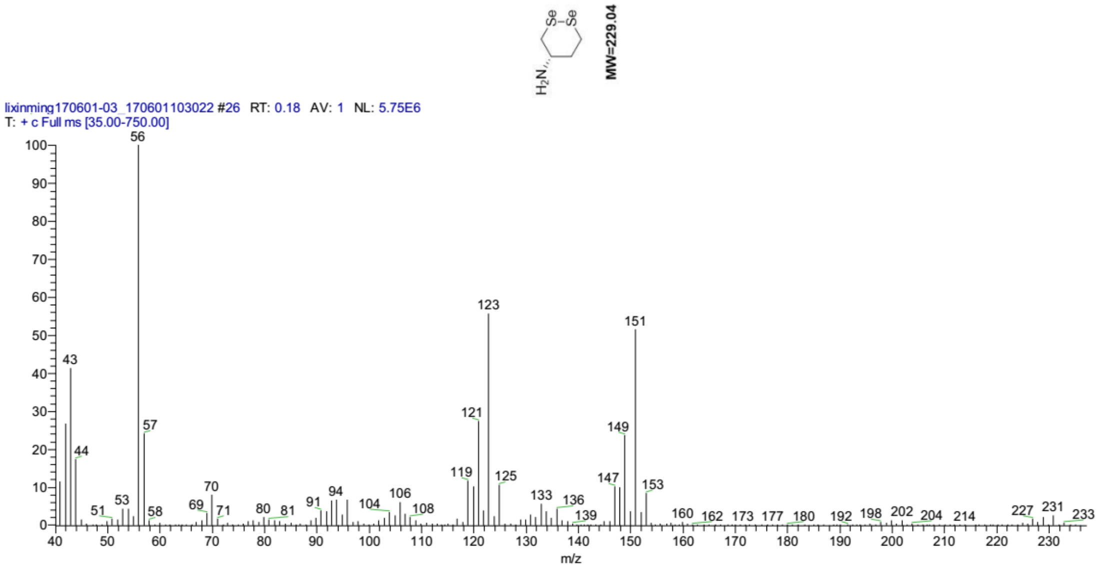

Supplementary Figure 40. MS Spectra of compound **8** (EI).

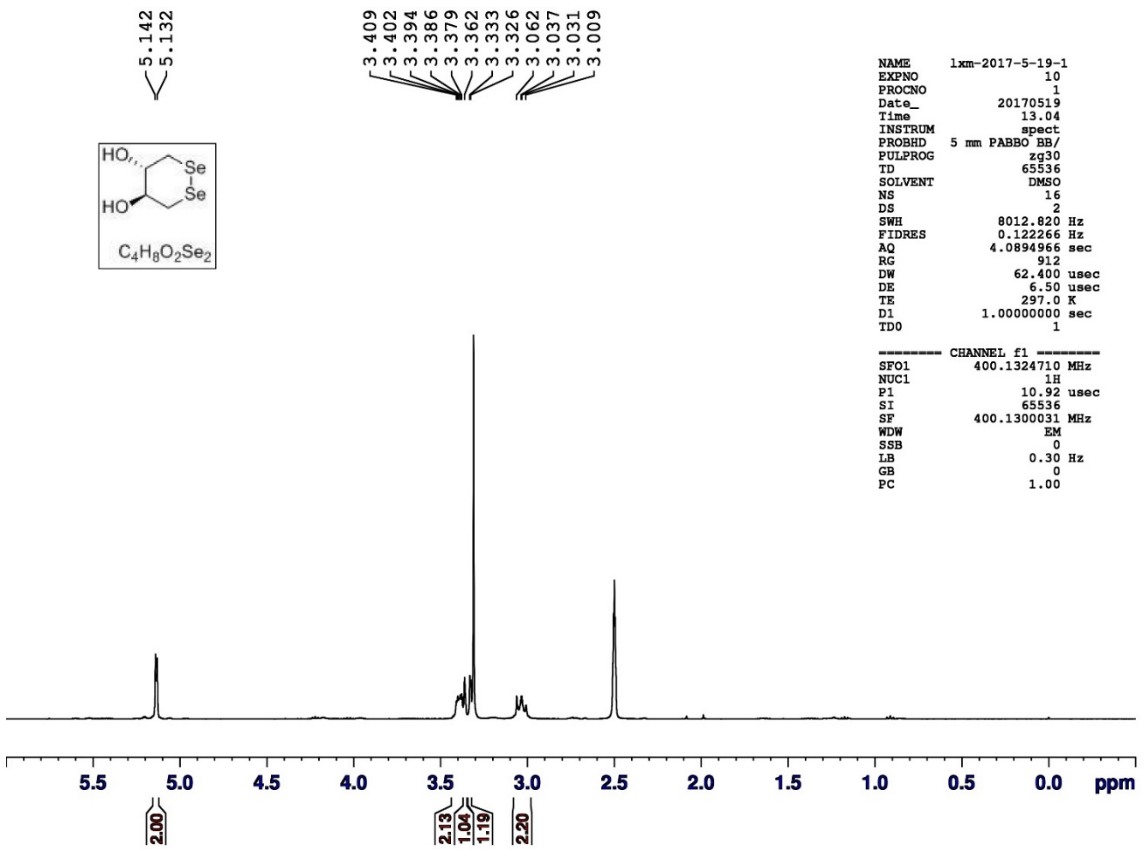

Supplementary Figure 41. ^{1}H NMR Spectra of compound **9** in DMSO-d_6 (400 MHz).

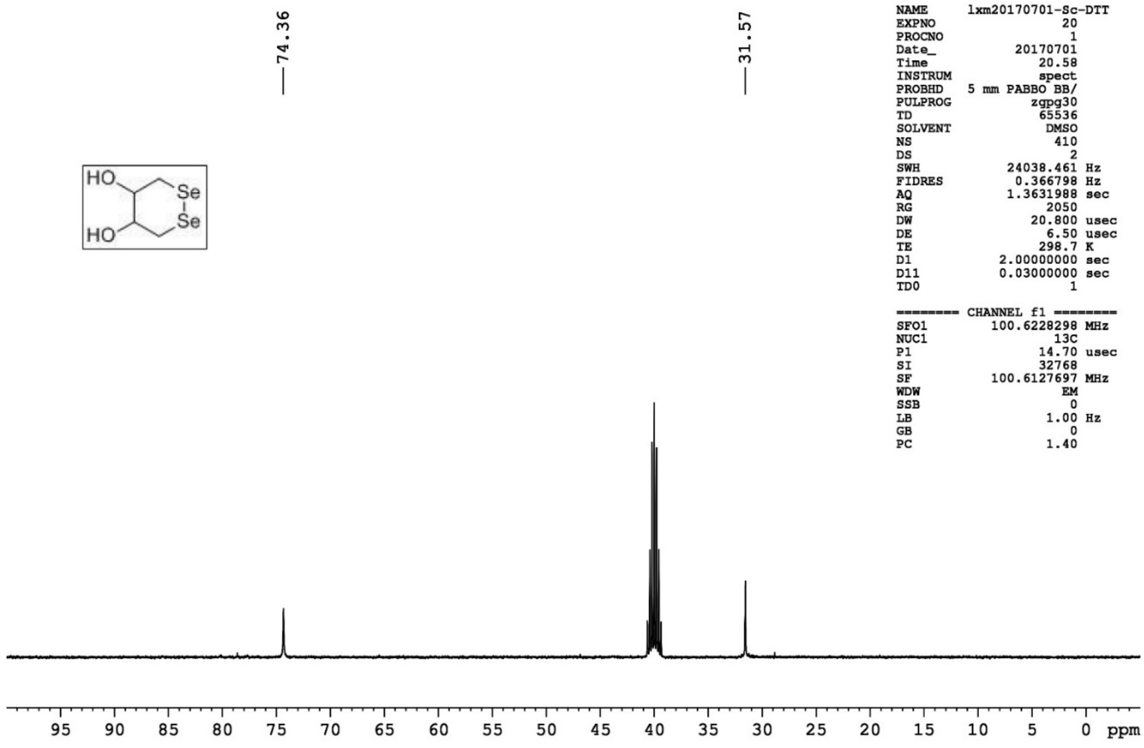

Supplementary Figure 42. ^{13}C NMR Spectra of compound **9** in DMSO-d_6 (100 MHz).

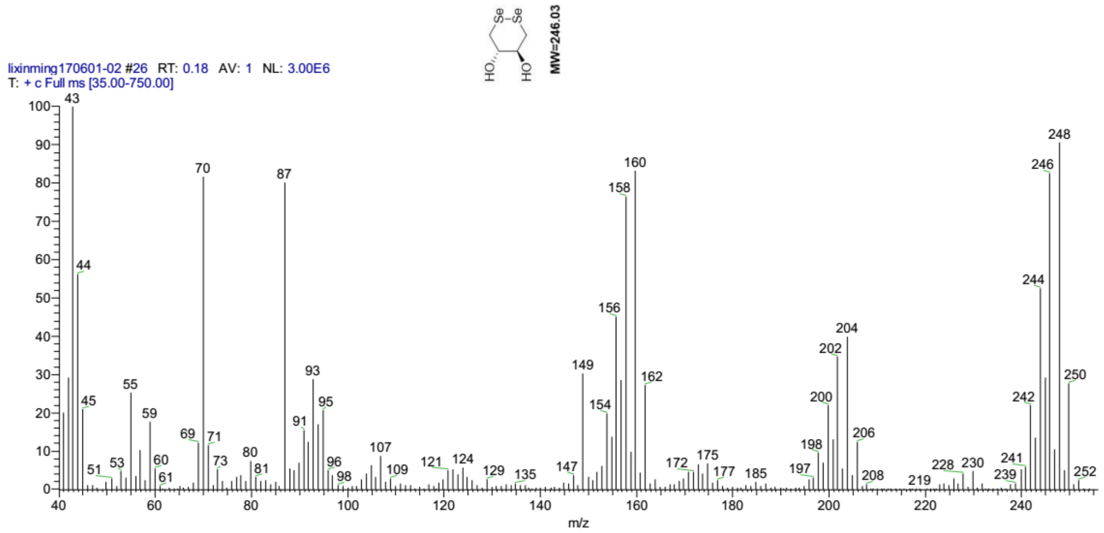

Supplementary Figure 43. MS Spectra of compound **9** (EI).

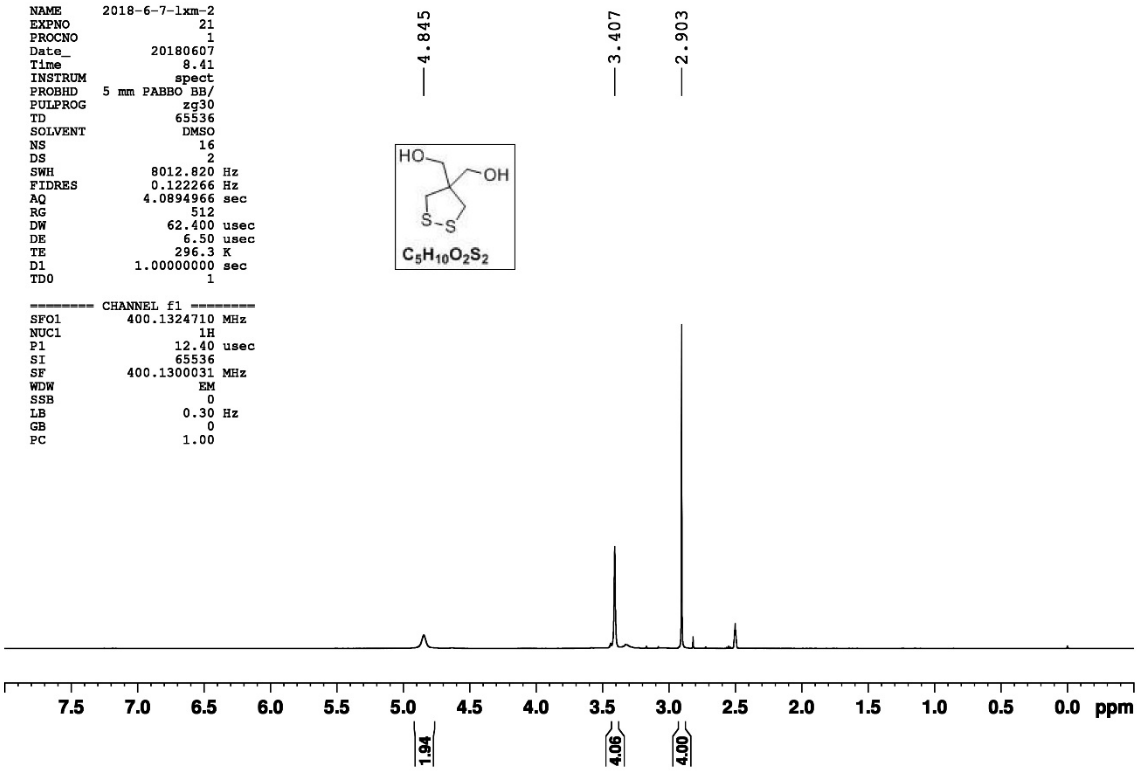

Supplementary Figure 44. ^{1}H NMR Spectra of compound **10** in DMSO-d$_6$ (400 MHz).

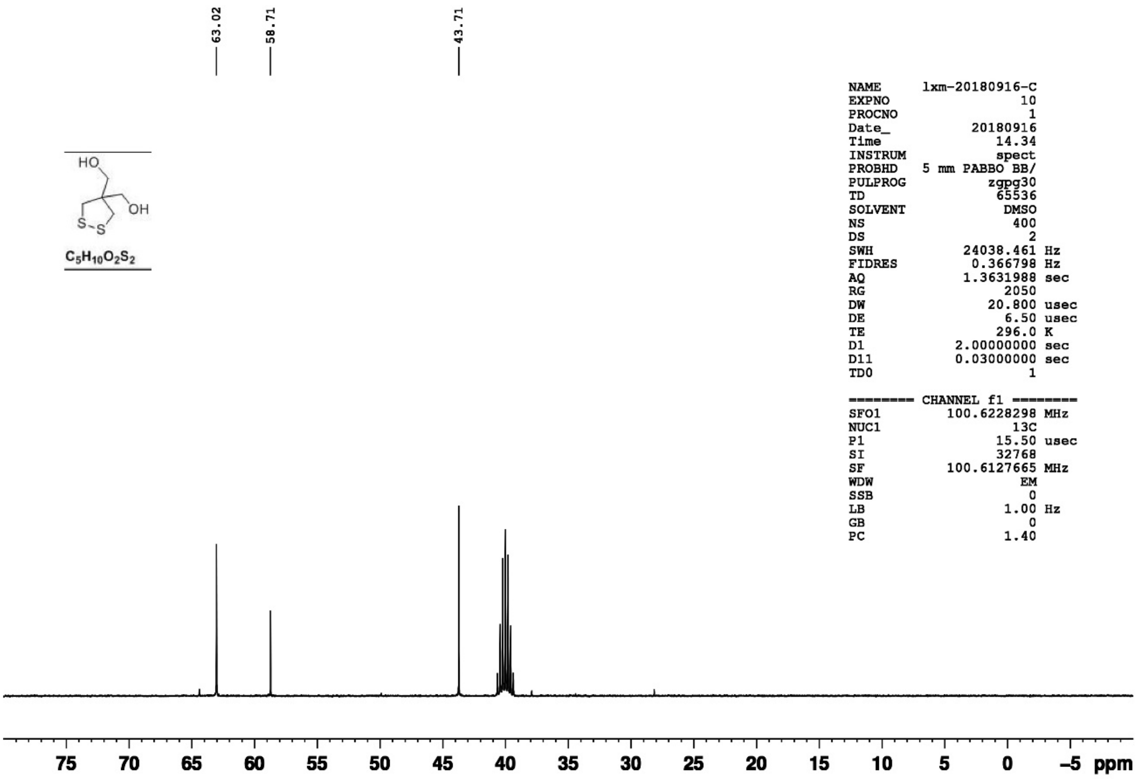

Supplementary Figure 45. ^{13}C NMR Spectra of compound **10** in DMSO-d_6 (100 MHz).

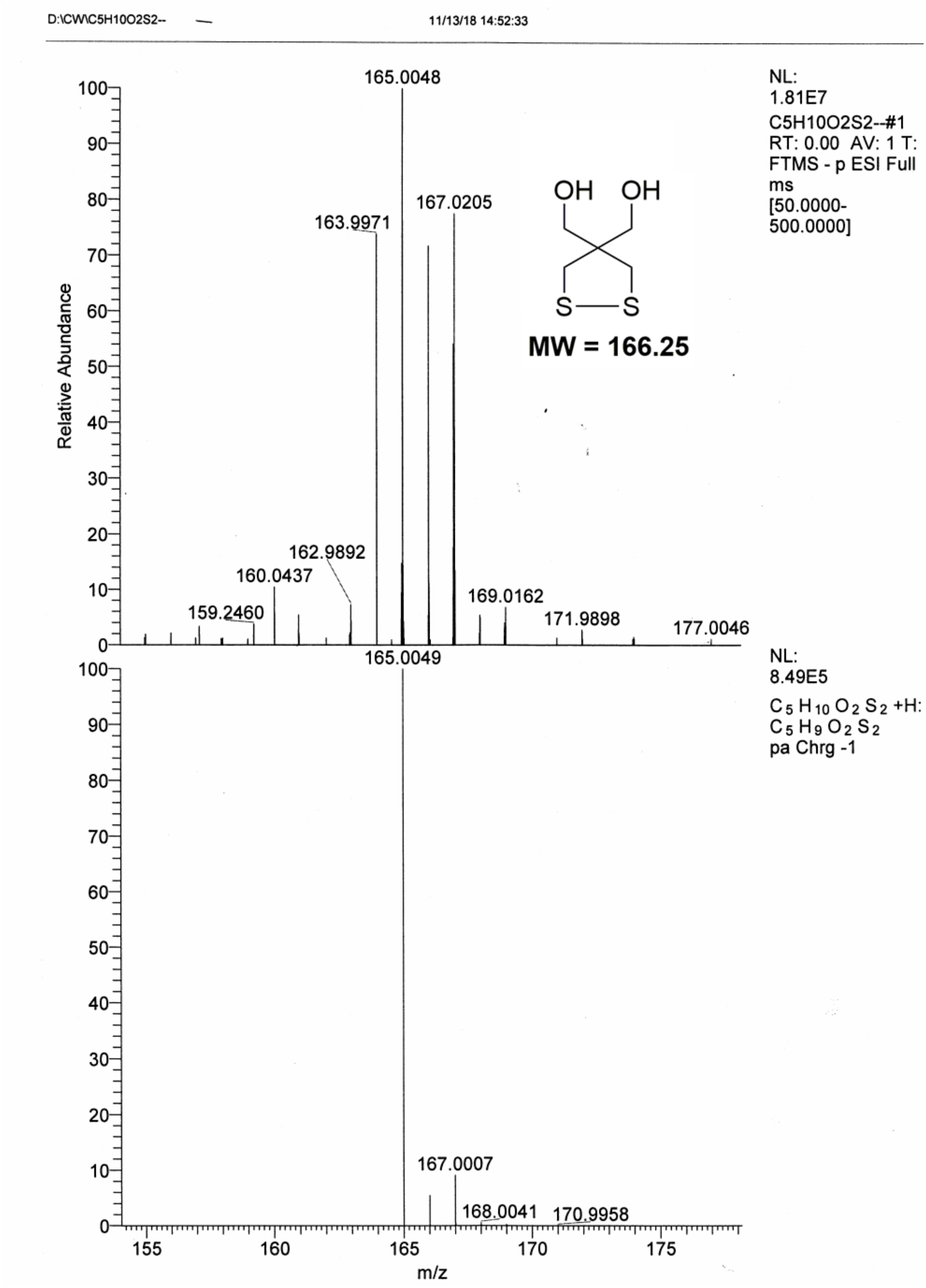

Supplementary Figure 46. MS Spectra of compound **10** (ESI).

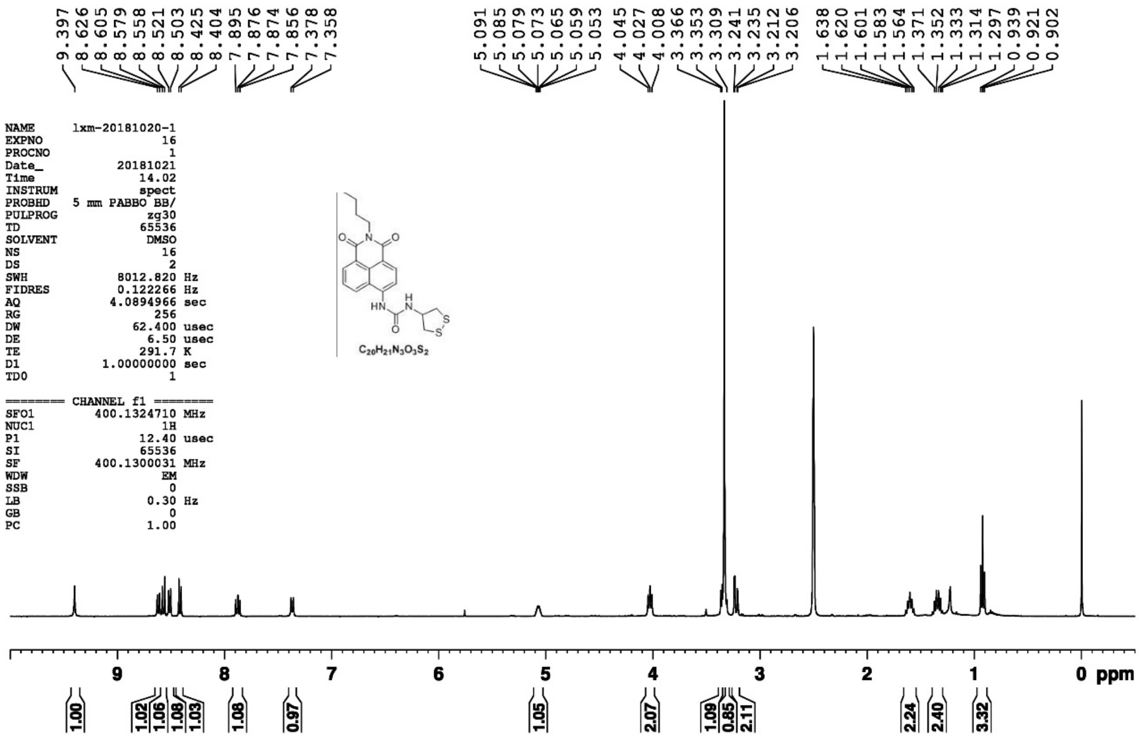

Supplementary Figure 47. ^{1}H NMR Spectra of **TRFS3** in DMSO-d_6 (400 MHz).

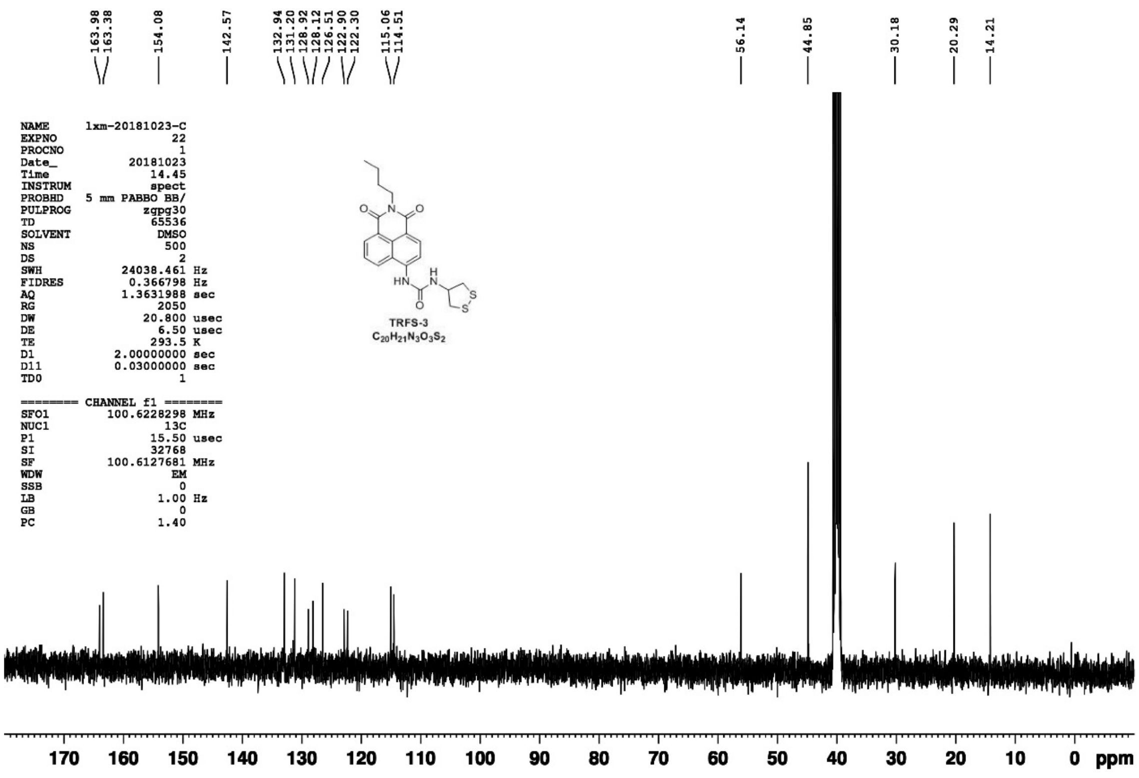

Supplementary Figure 48. ^{13}C NMR Spectra of **TRFS3** in DMSO-d_6 (100 MHz)

SHIMADZU LabSolutions 分析报告

TRFS-3

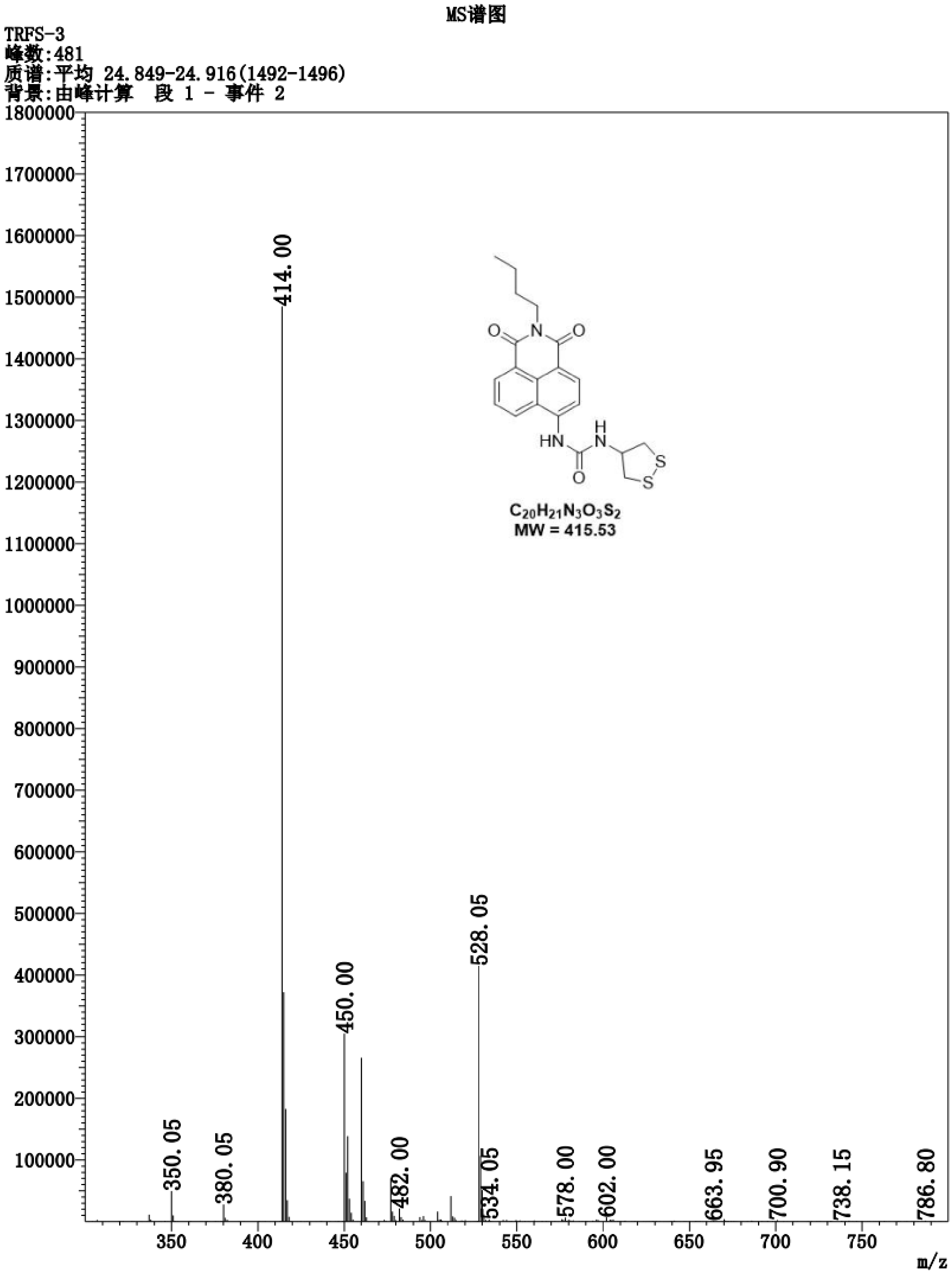

Supplementary Figure 49. MS Spectra of **TRFS3** (ESI).

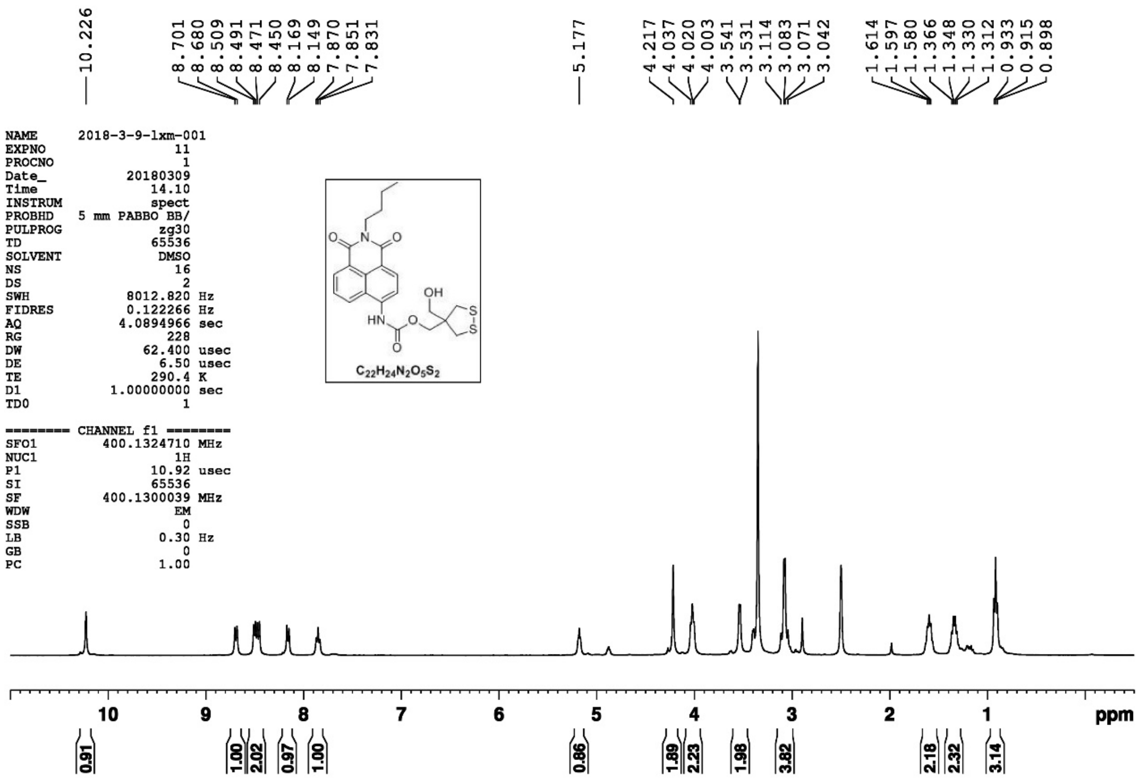

Supplementary Figure 50. ^{1}H NMR Spectra of **TRFS4** in DMSO-d_6 (400 MHz)

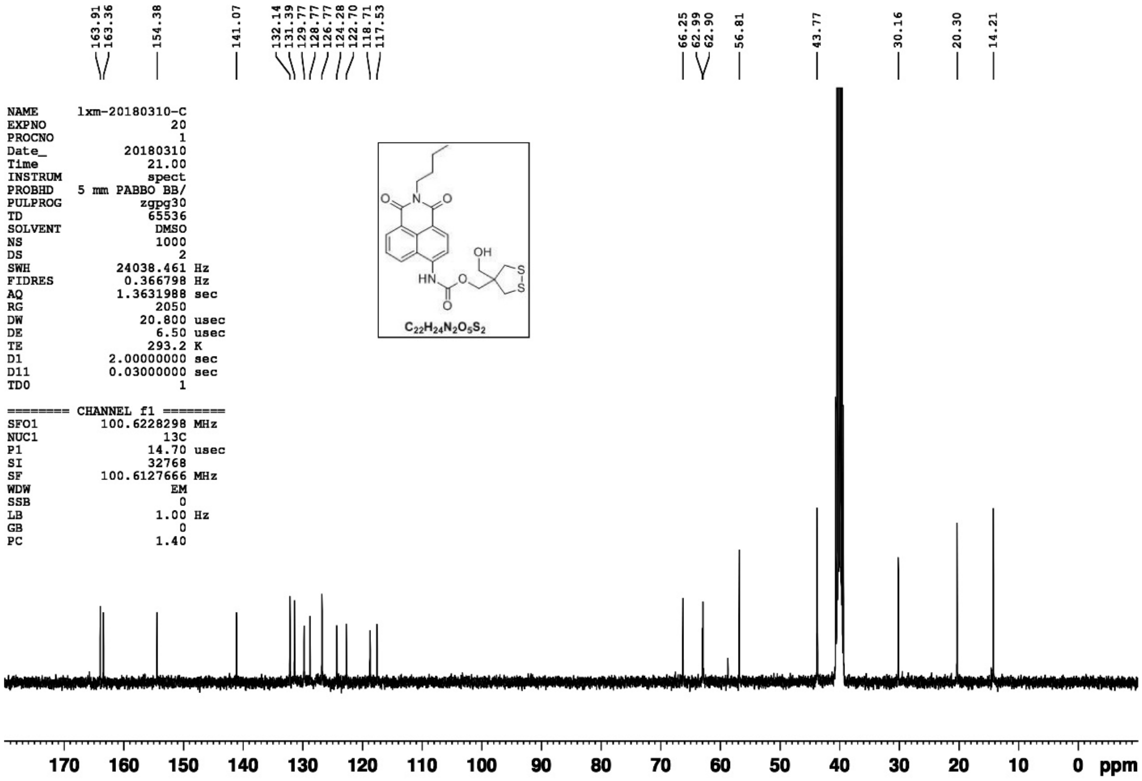

Supplementary Figure 51. ^{13}C NMR Spectra of **TRFS4** in DMSO-d_6 (100 MHz)

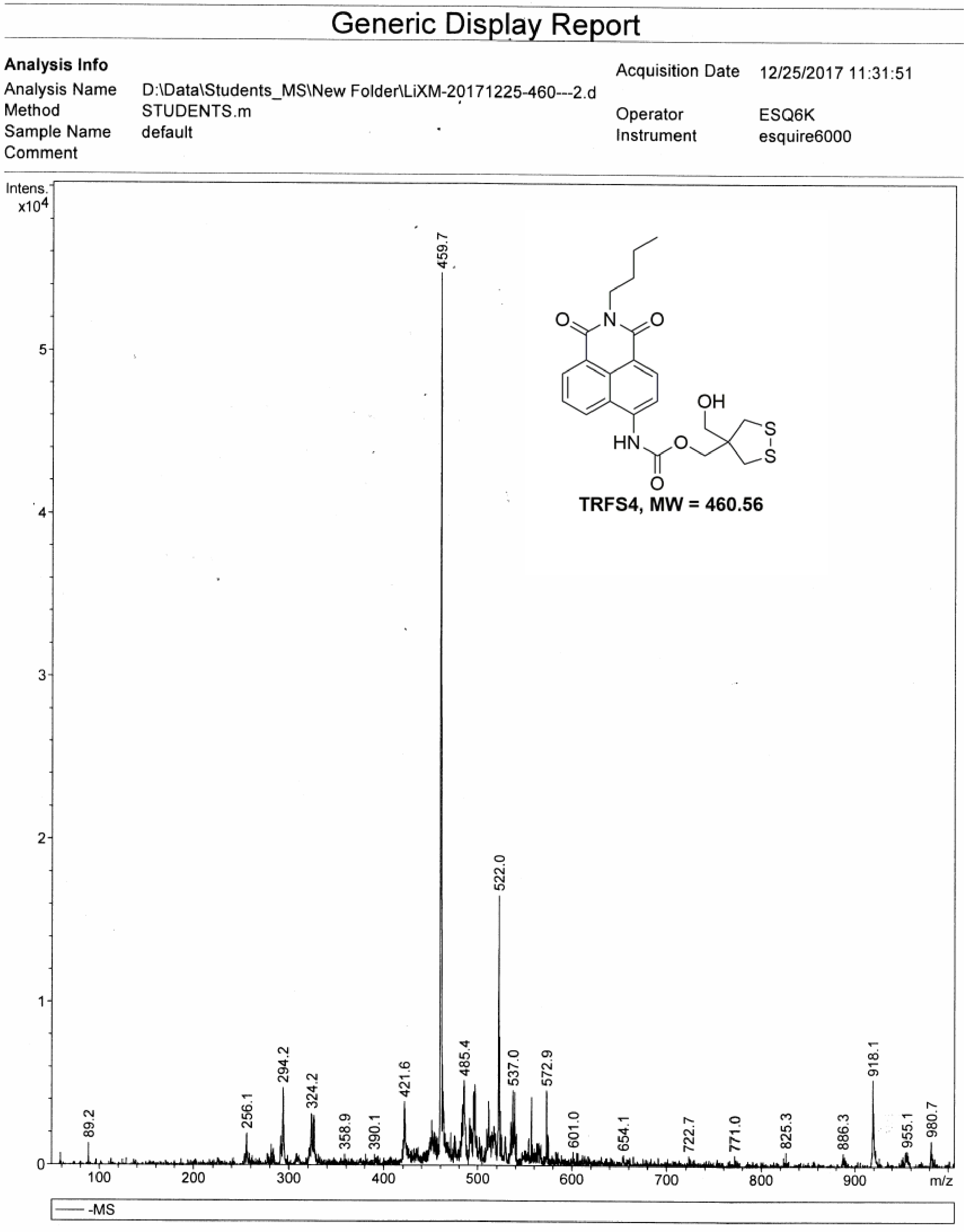

Supplementary Figure 52. MS Spectra of **TRFS4** (ESI).

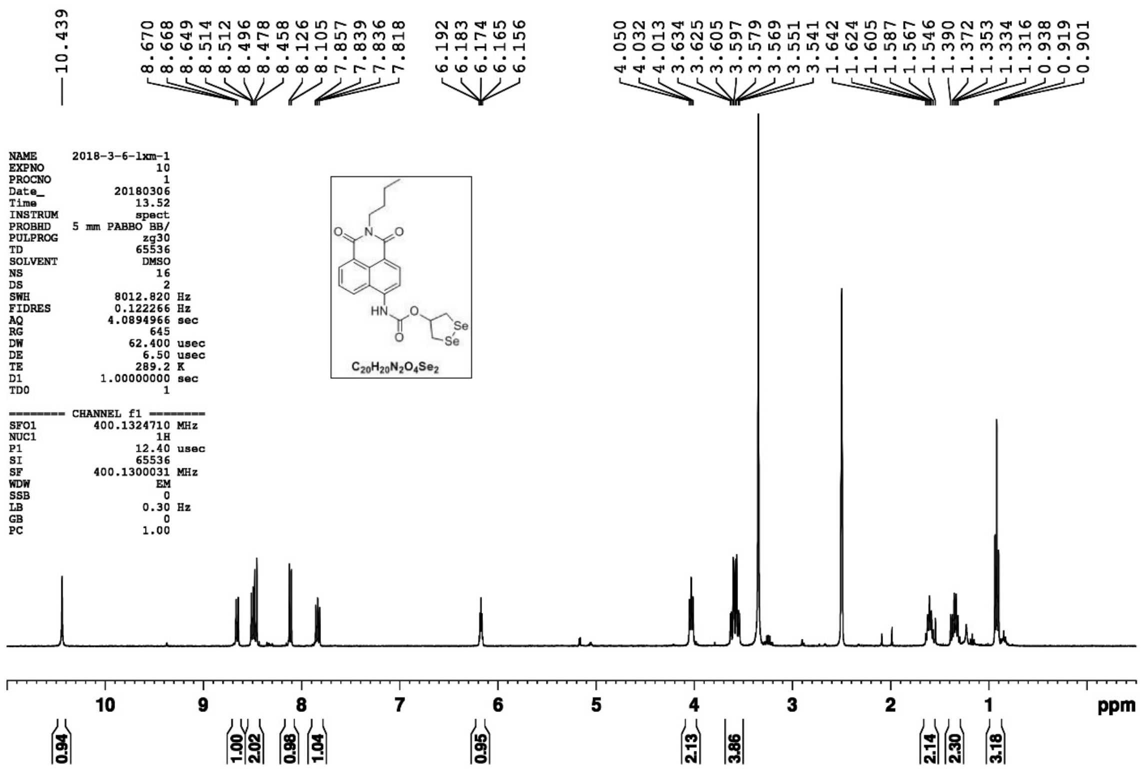

Supplementary Figure 53. ^{1}H NMR Spectra of **TRFS5** in DMSO-d_6 (400 MHz)

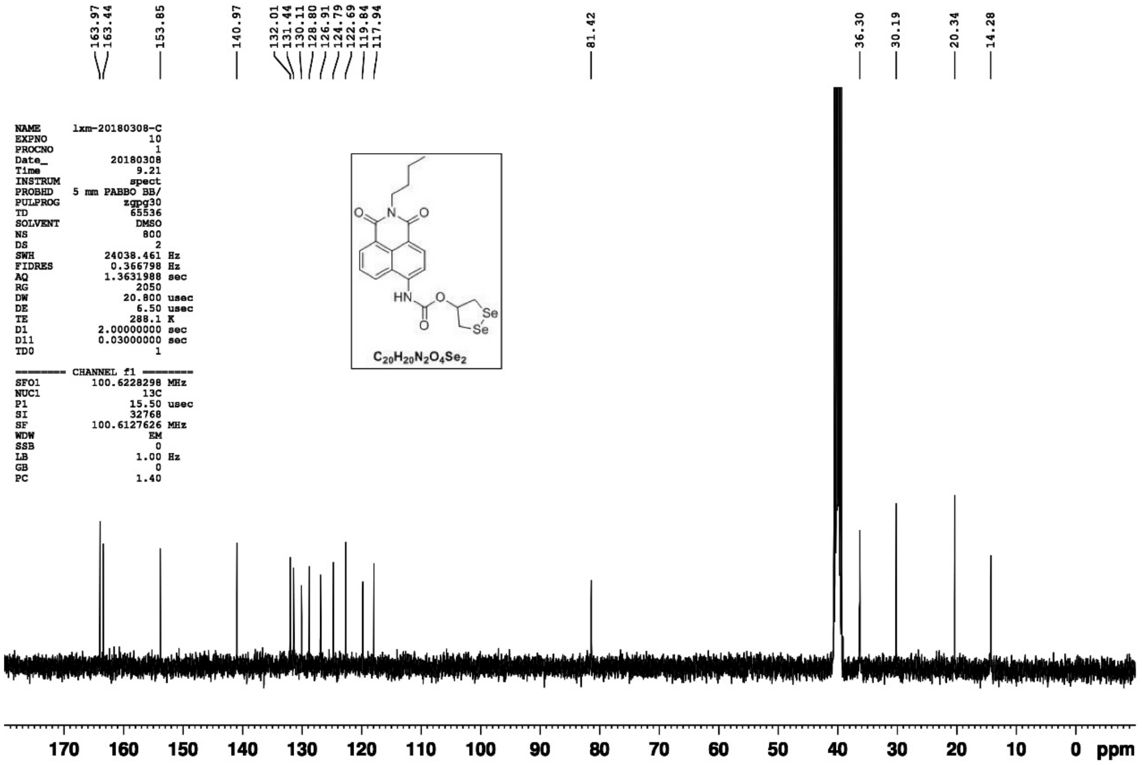

Supplementary Figure 54. ^{13}C NMR Spectra of **TRFS5** in DMSO-d_6 (100 MHz).

![SHIMADZU LabSolutions] 分析报告

TRFS-4

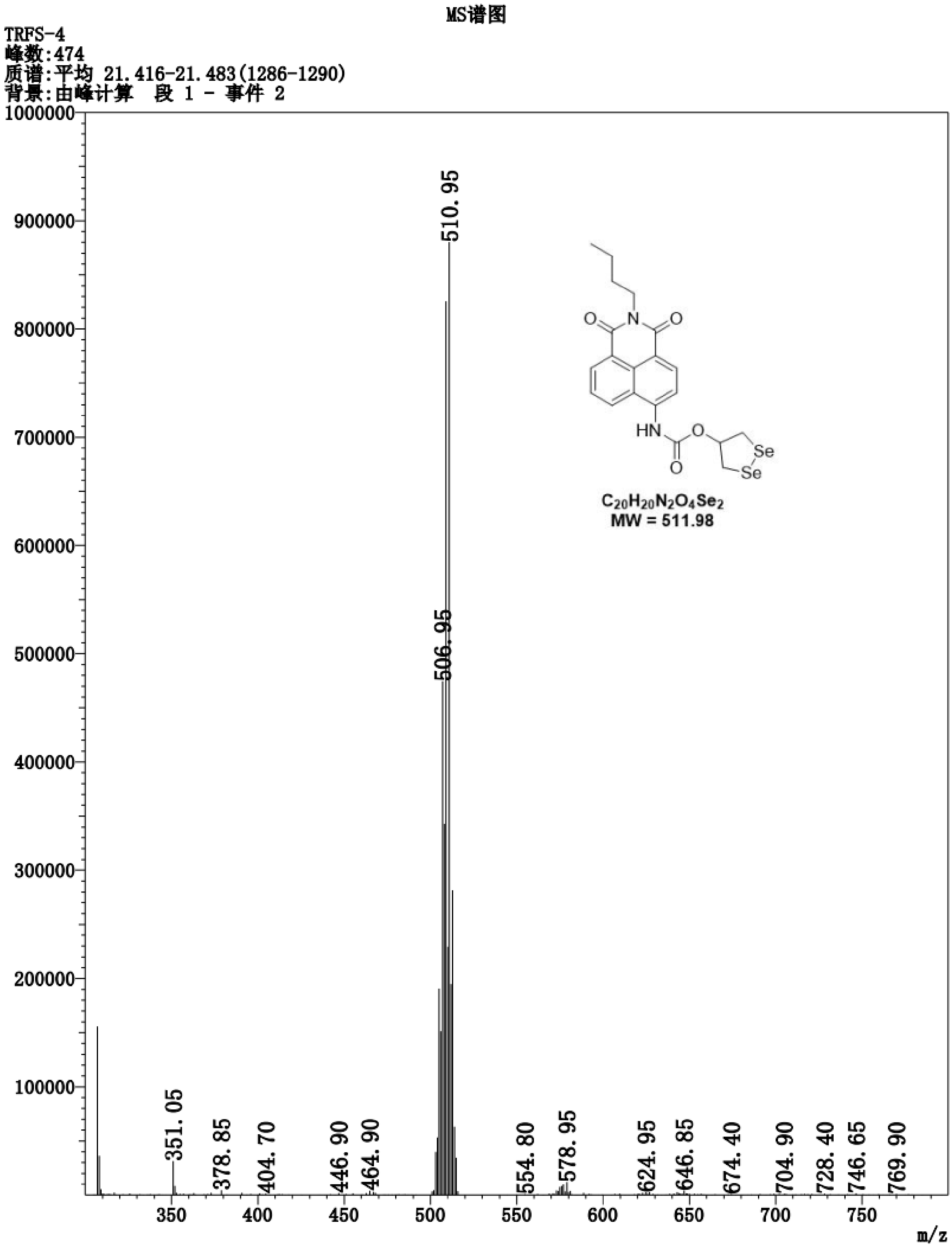

Supplementary Figure 55. MS Spectra of **TRFS5** (ESI).

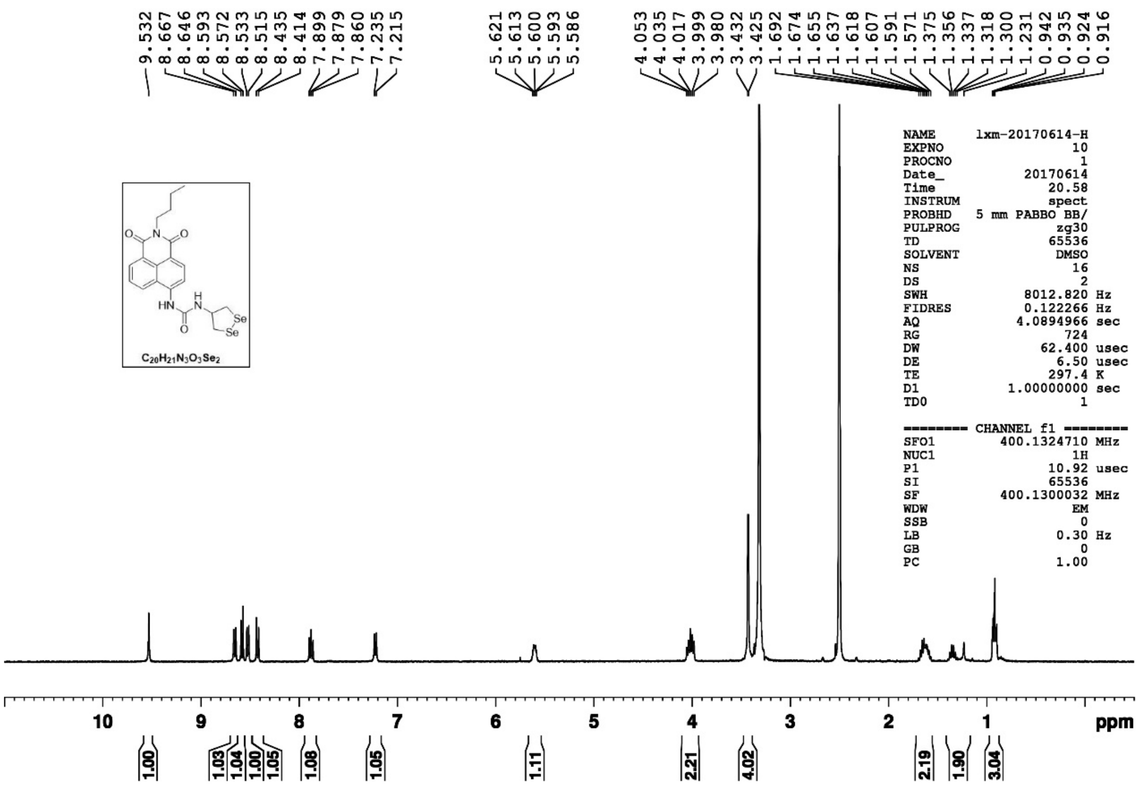

Supplementary Figure 56. ^{1}H NMR Spectra of **TRFS6** in DMSO-d_6 (400 MHz).

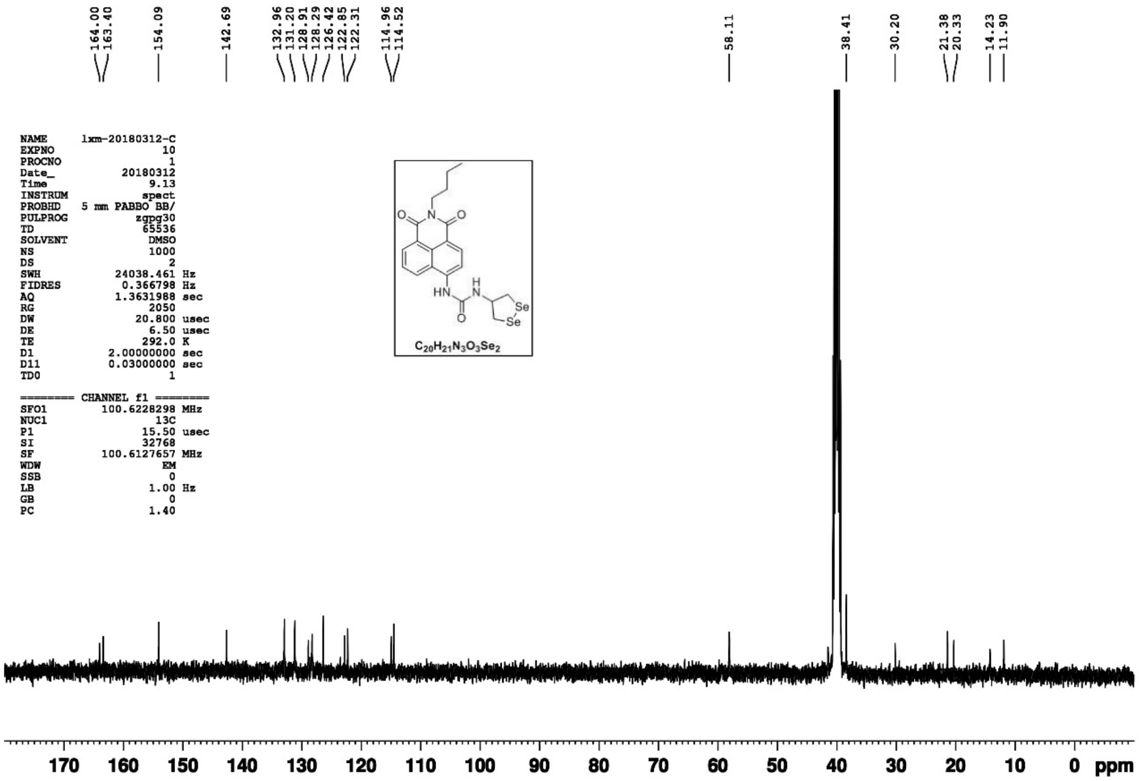

Supplementary Figure 57. ^{13}C NMR of **TRFS6** in DMSO-d_6 (100 MHz).

SHIMADZU LabSolutions 分析报告

TRFS-5

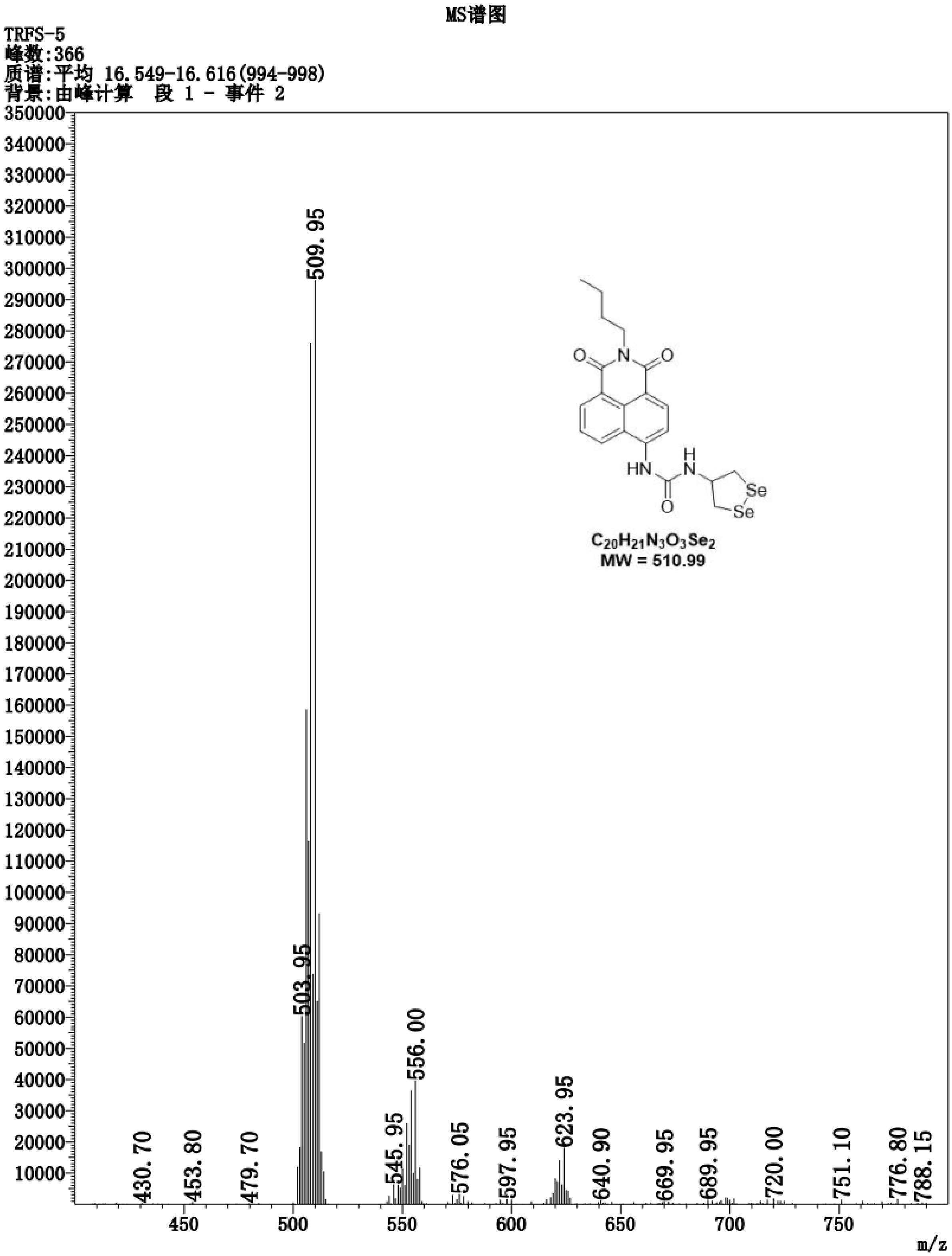

Supplementary Figure 58. MS Spectra of **TRFS6** (ESI)

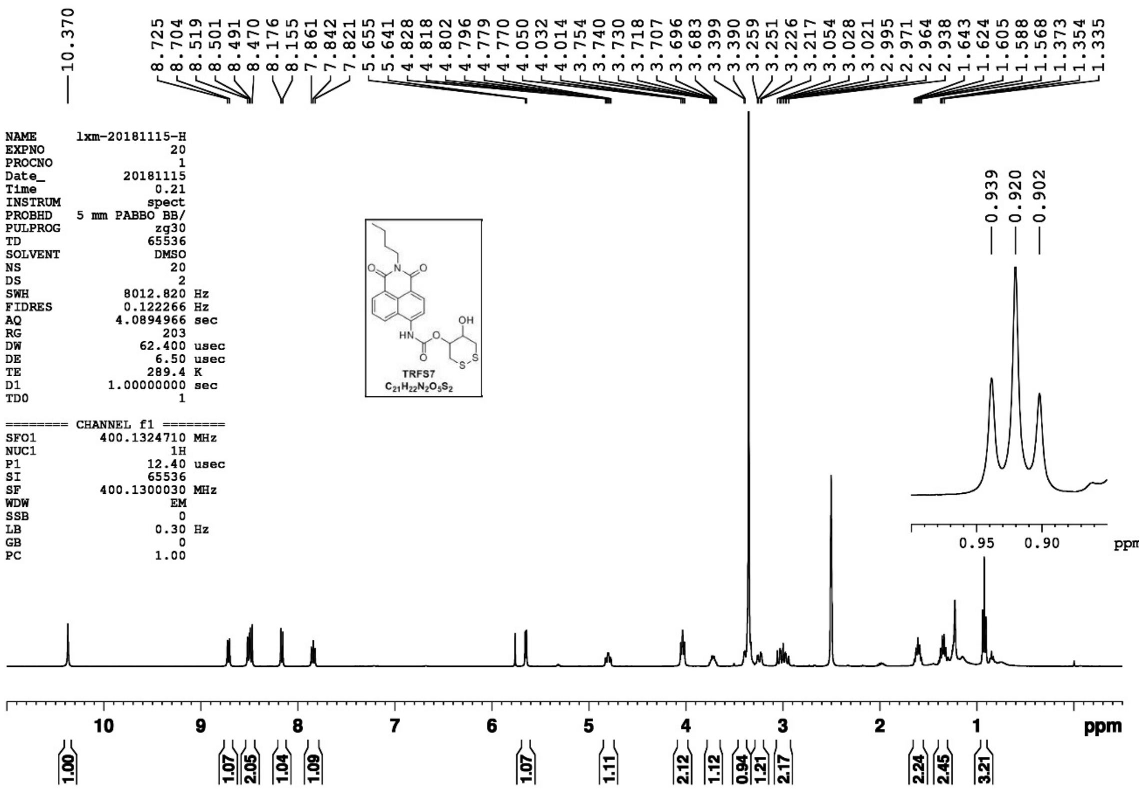

Supplementary Figure 59. ^{1}H NMR Spectra of **TRFS7** in DMSO-d_6 (400 MHz)

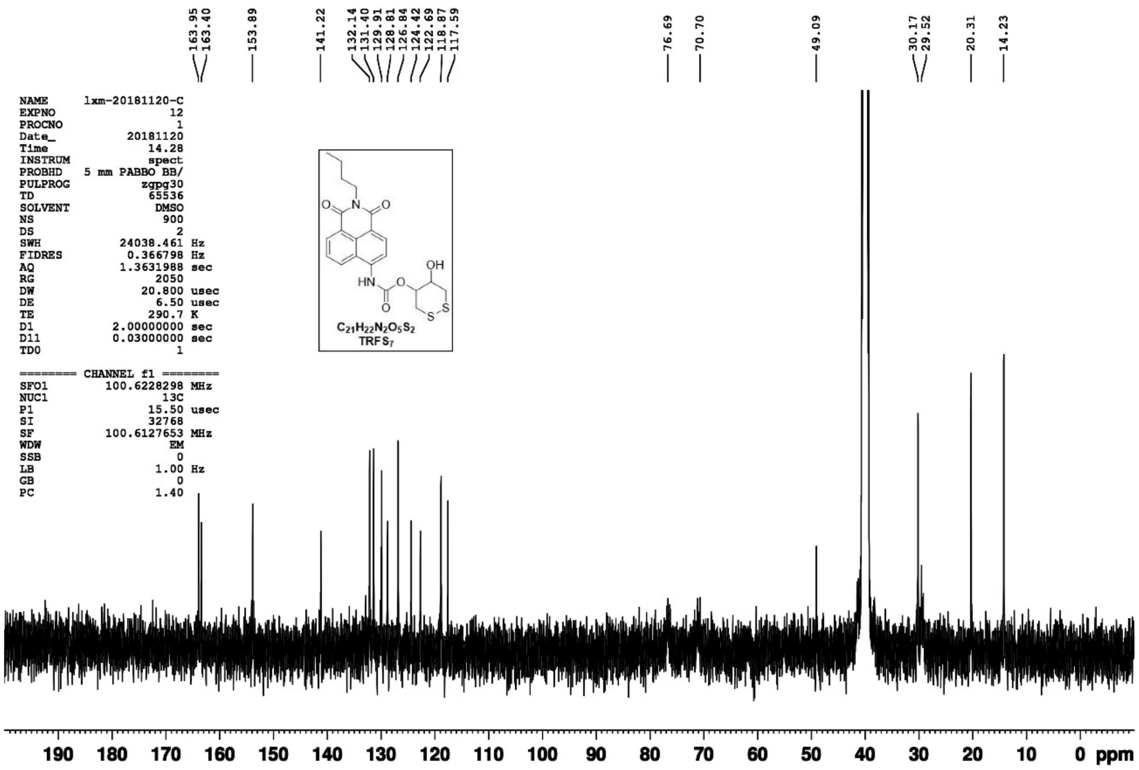

Supplementary Figure 60. ^{13}C NMR Spectra of **TRFS7** in DMSO-d_6 (100 MHz)

Generic Display Report

Analysis Info

Analysis Name	D:\Data\Students_MS\New Folder\LiXM-20180110-446.d	Acquisition Date	1/10/2018 11:30:05
Method	STUDENTS.m	Operator	ESQ6K
Sample Name	default	Instrument	esquire6000
Comment			

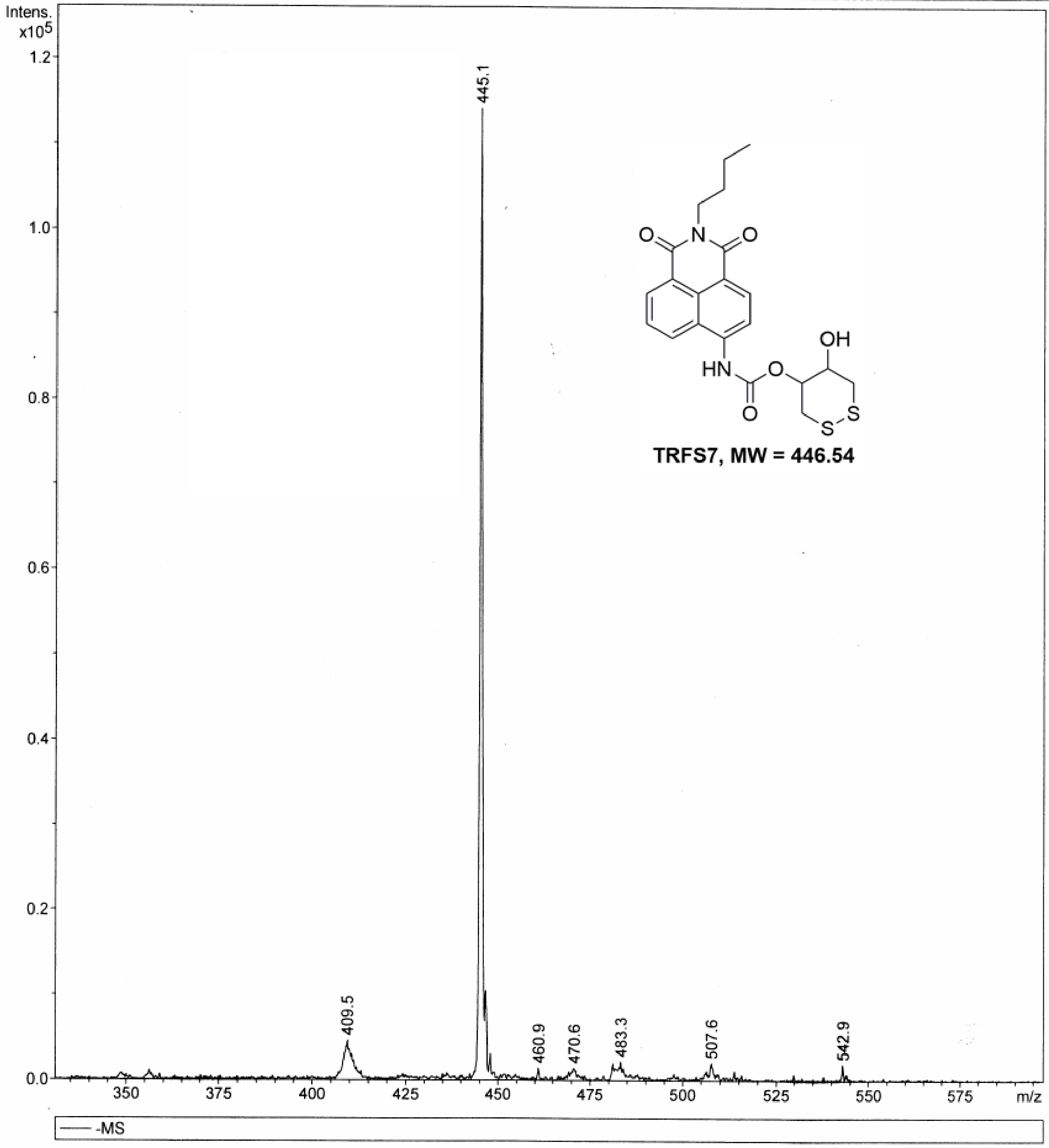

Bruker Daltonics DataAnalysis 3.4 printed: 1/10/2018 11:31:13 Page 1 of 1

Supplementary Figure 61. MS Spectra of **TRFS7** (ESI).

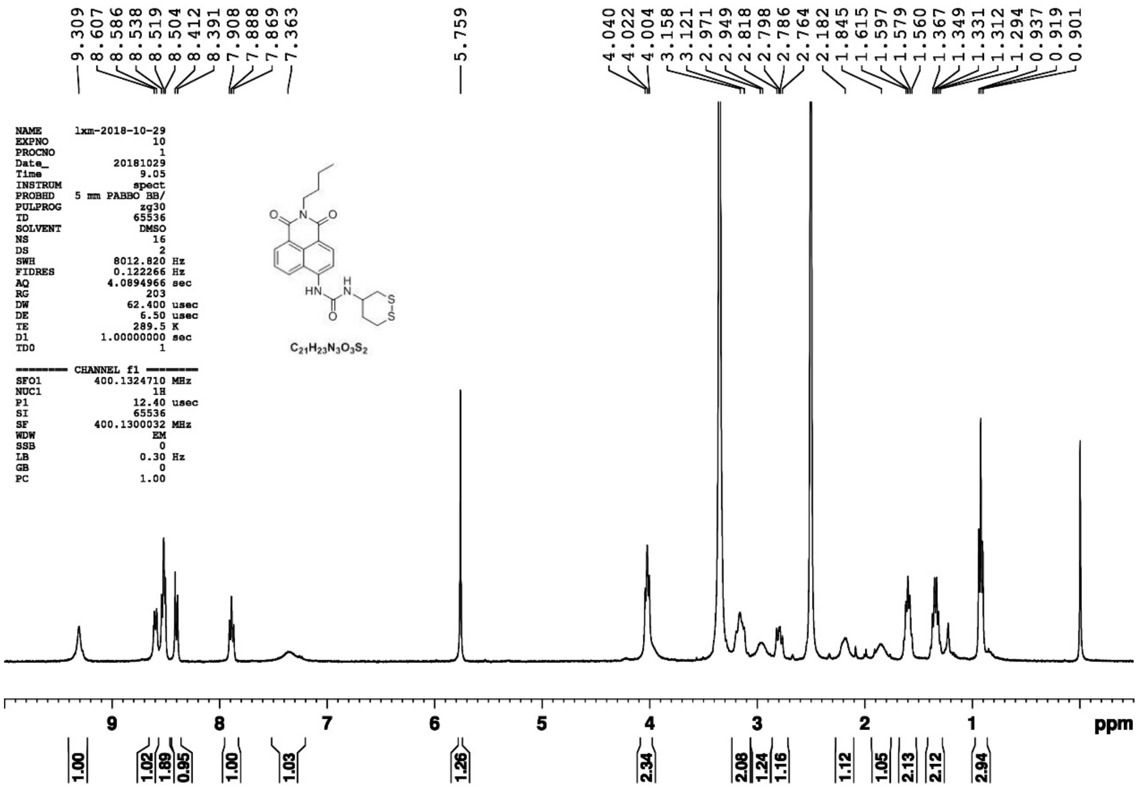

Supplementary Figure 62. ^{1}H NMR Spectra of **TRFS8** in DMSO-d_6 (400 MHz).

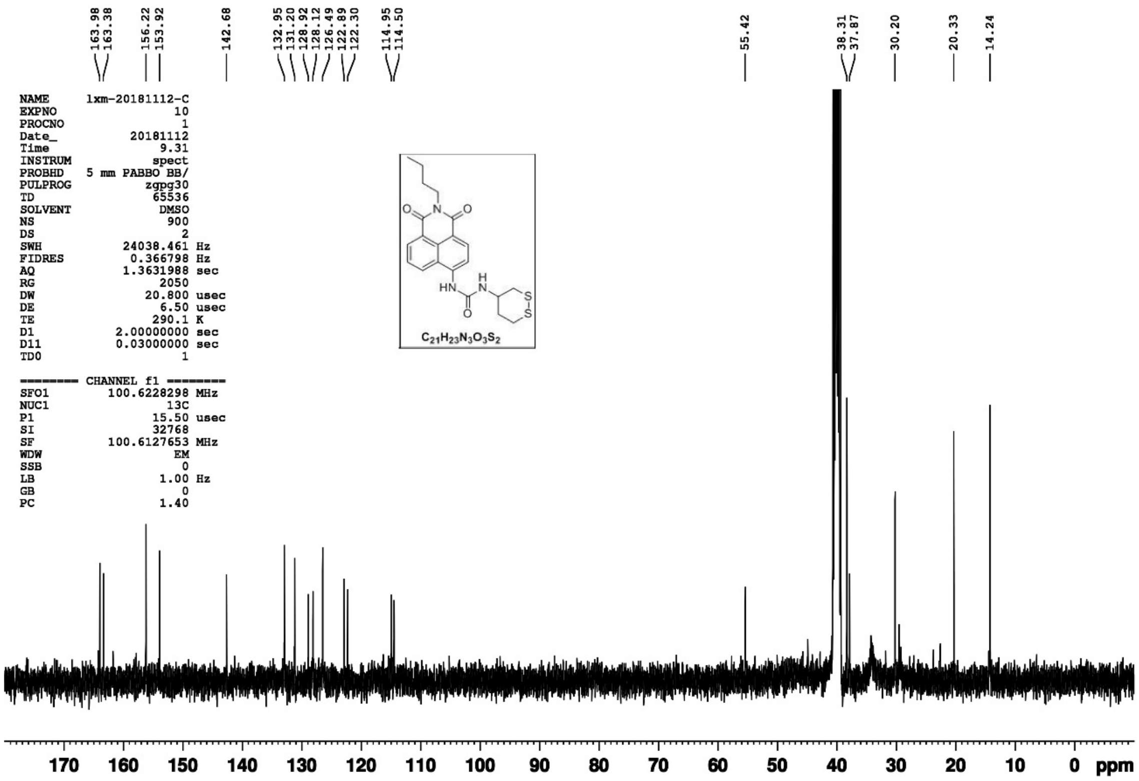

Supplementary Figure 63. ^{13}C NMR Spectra of **TRFS8** in DMSO-d_6 (100 MHz).

SHIMADZU LabSolutions 分析报告

TRFS-9

MS谱图

TRFS-9
峰数:478
质谱:平均 14.149-14.216(850-854)
背景:由峰计算　段 1 - 事件 2

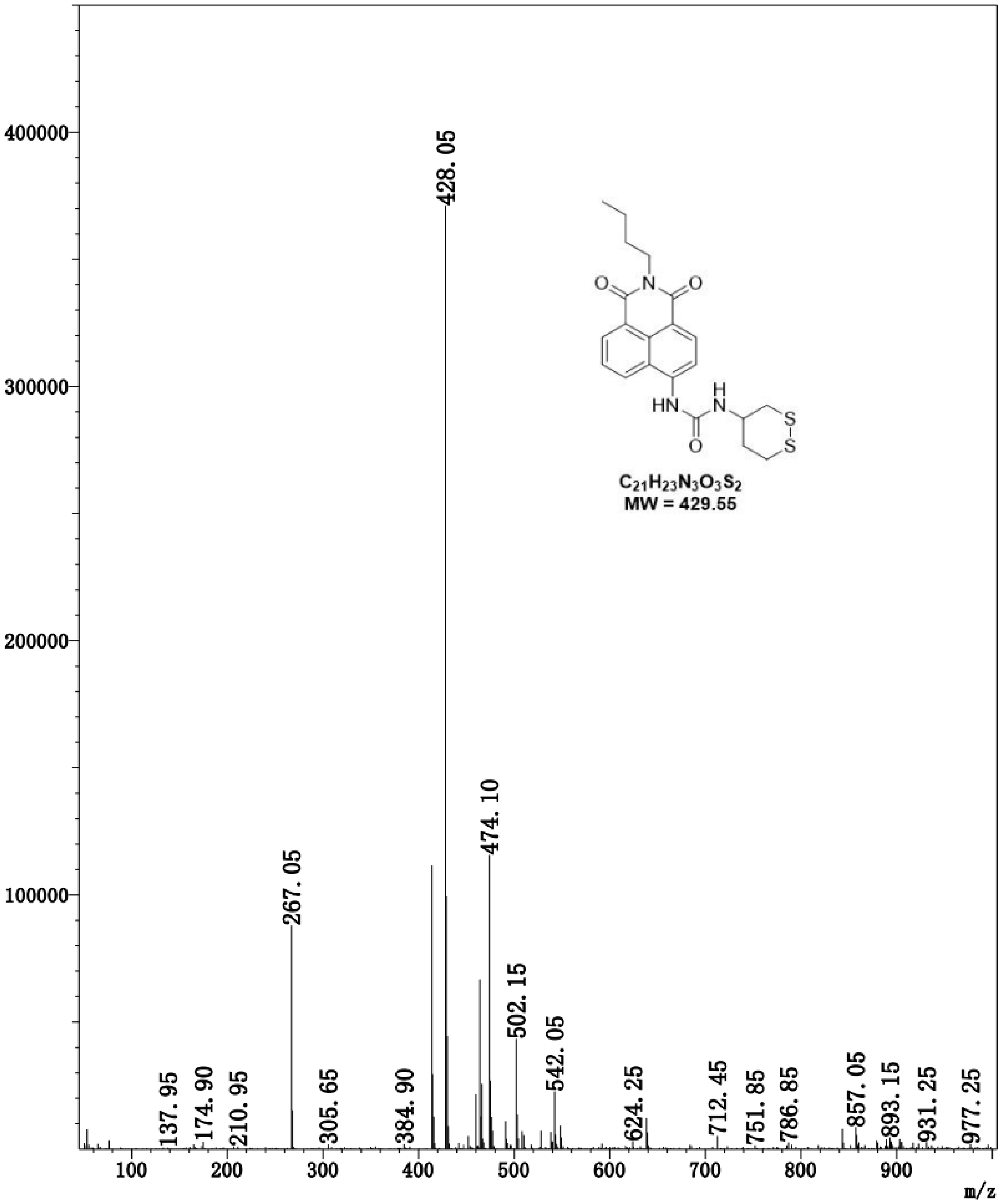

Supplementary Figure 64. MS Spectra of **TRFS8** (ESI).

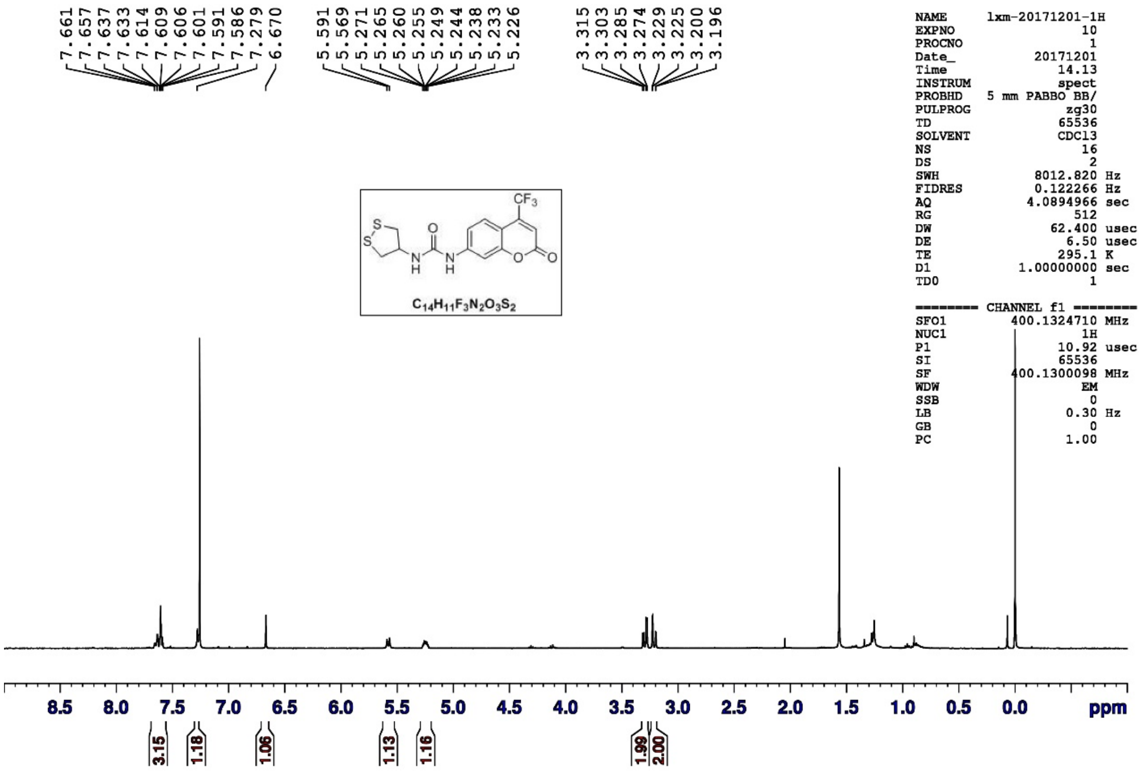

Supplementary Figure 65. ^{1}H NMR Spectra of **TRFS9** in DMSO-d$_6$ (400 MHz).

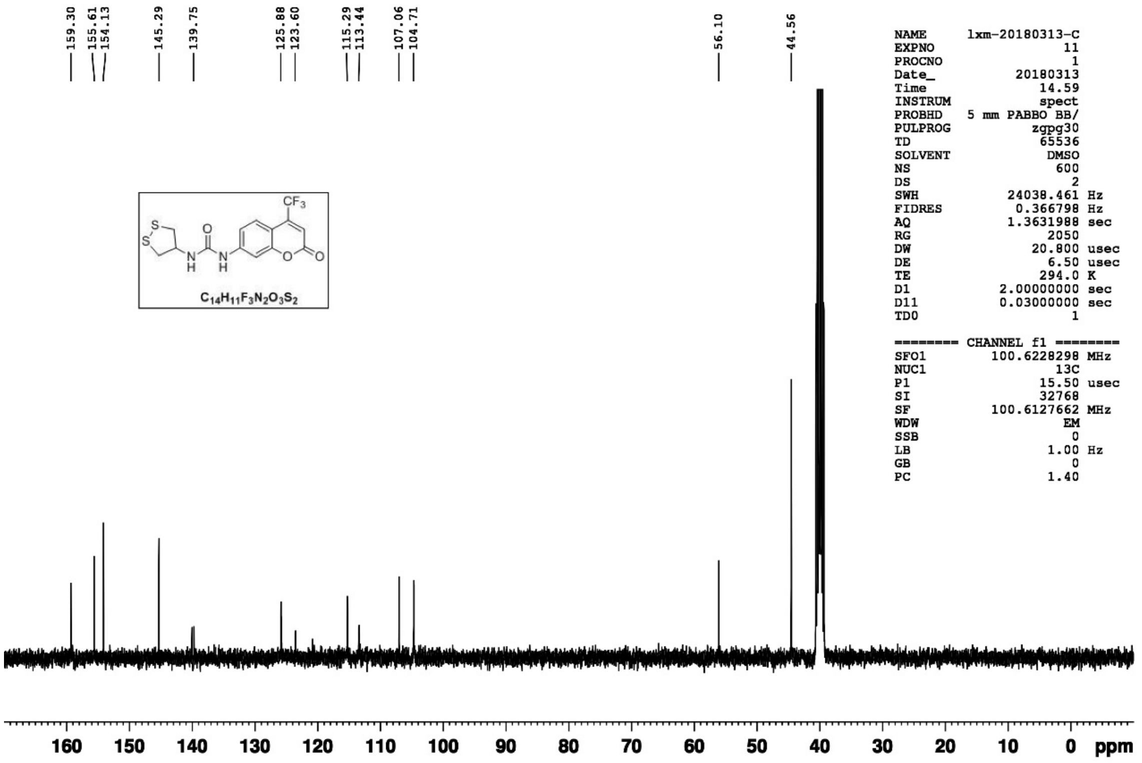

Supplementary Figure 66. ^{13}C NMR Spectra of **TRFS9** in DMSO-d$_6$ (100 MHz).

Generic Display Report

Analysis Info

Analysis Name	D:\Data\Students_MS\New Folder\LiXM-20171122-TRFS-1-376--.d	Acquisition Date	11/22/2017 11:37:00
Method	STUDENTS.m	Operator	ESQ6K
Sample Name	default	Instrument	esquire6000
Comment			

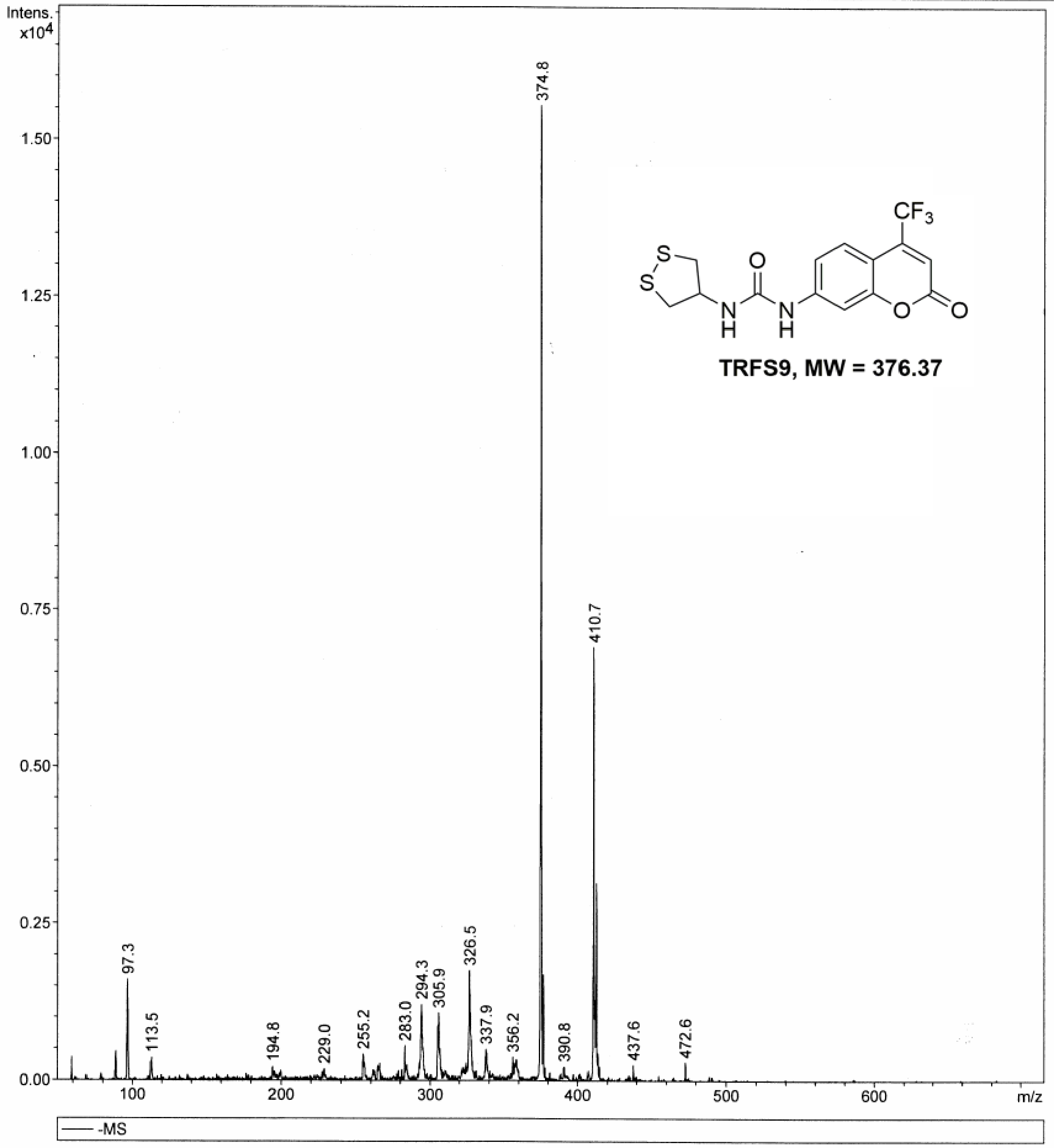

Bruker Daltonics DataAnalysis 3.4 printed: 11/22/2017 11:39:14 Page 1 of 1

Supplementary Figure 67. MS Spectra of **TRFS9** (ESI).

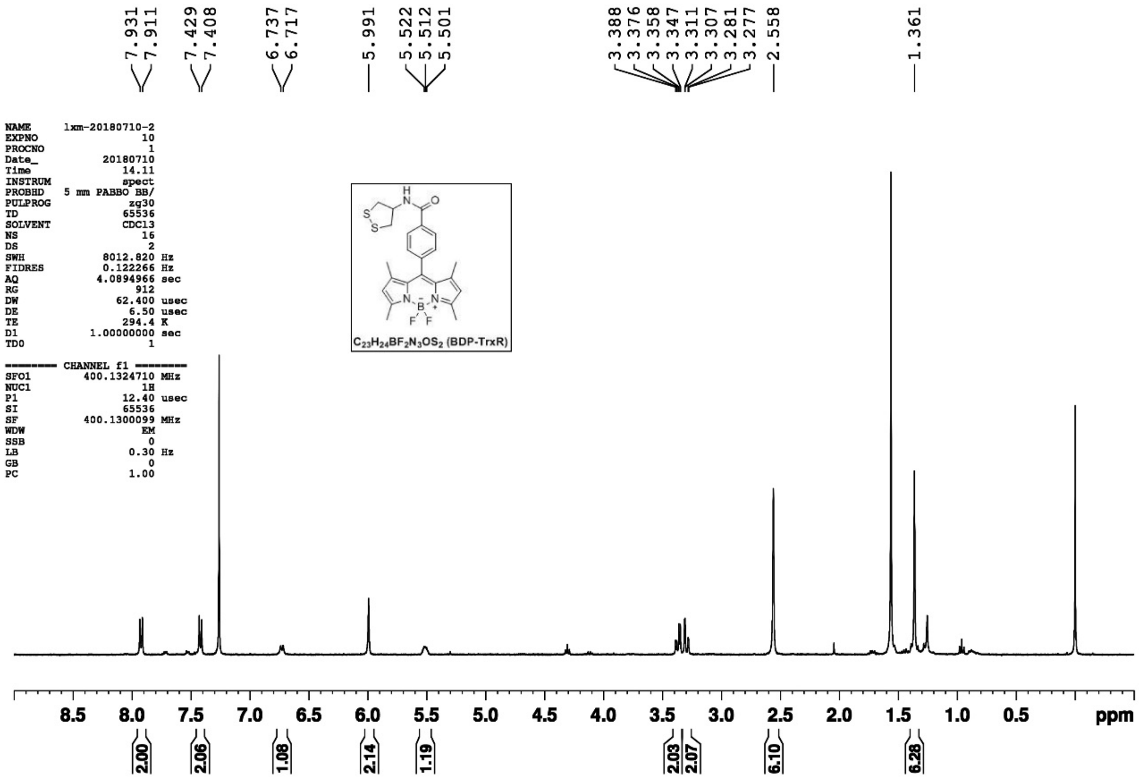

Supplementary Figure 68. ^{1}H NMR Spectra of **TRFS10** in CDCl3 (400 MHz).

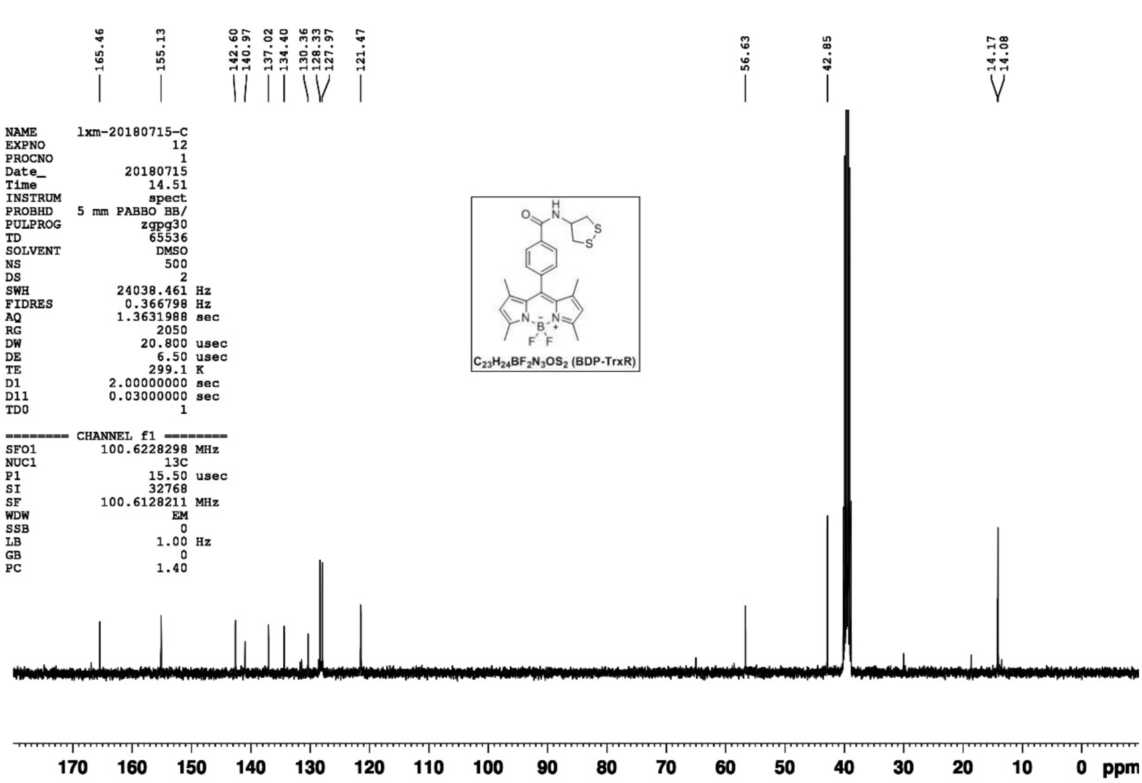

Supplementary Figure 69. ^{13}C NMR Spectra of **TRFS10** in DMSO-d_6 (100 MHz).

Generic Display Report

Analysis Info

Analysis Name	D:\Data\Students_MS\New Folder\20180710-LXM-471.d	Acquisition Date	7/10/2018 11:23:13
Method	STUDENTS.m	Operator	ESQ6K
Sample Name	default	Instrument	esquire6000
Comment			

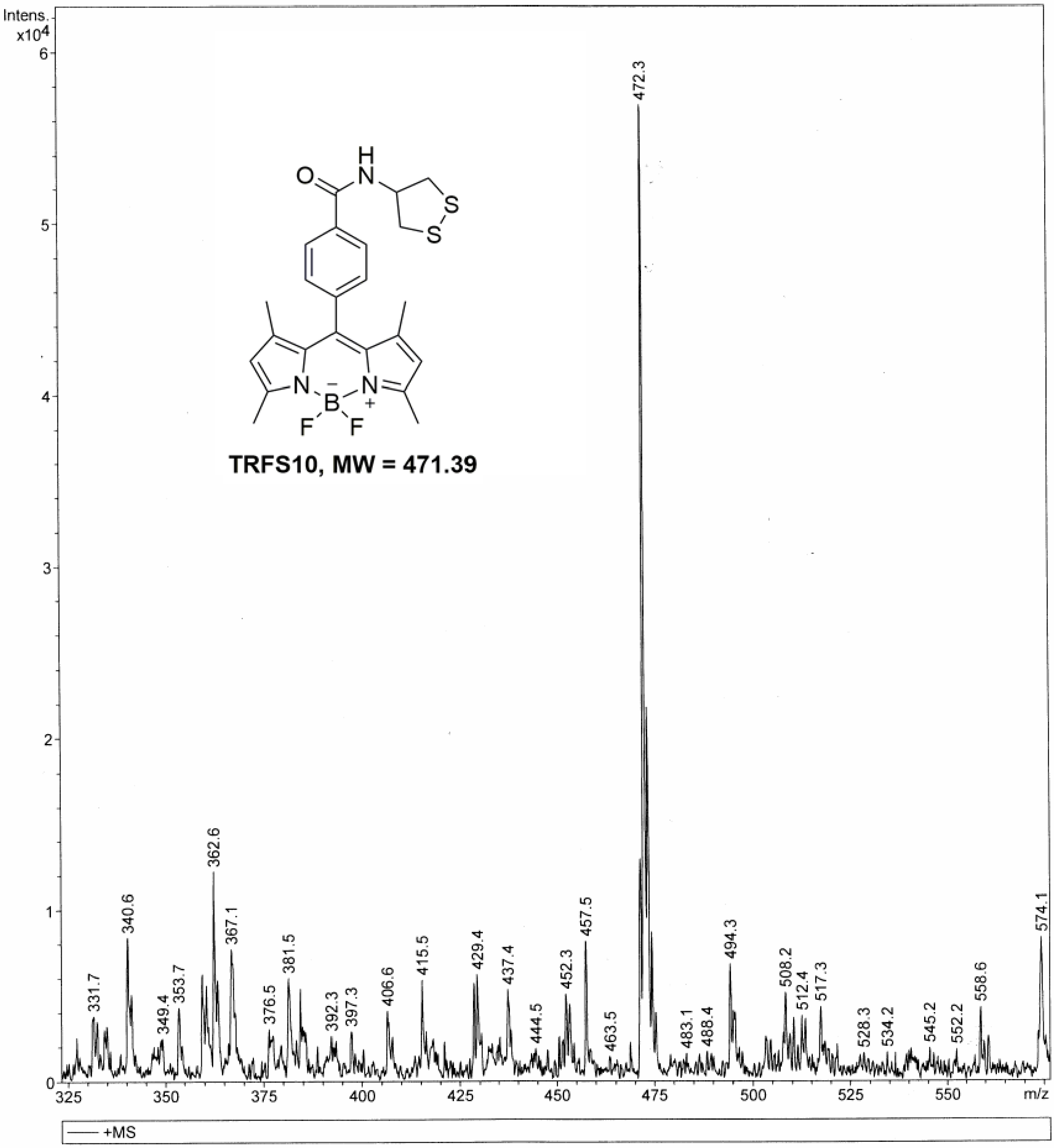

Bruker Daltonics DataAnalysis 3.4 printed: 7/10/2018 11:24:00 Page 1 of 1

Supplementary Figure 70. MS Spectra of **TRFS10** (ESI).

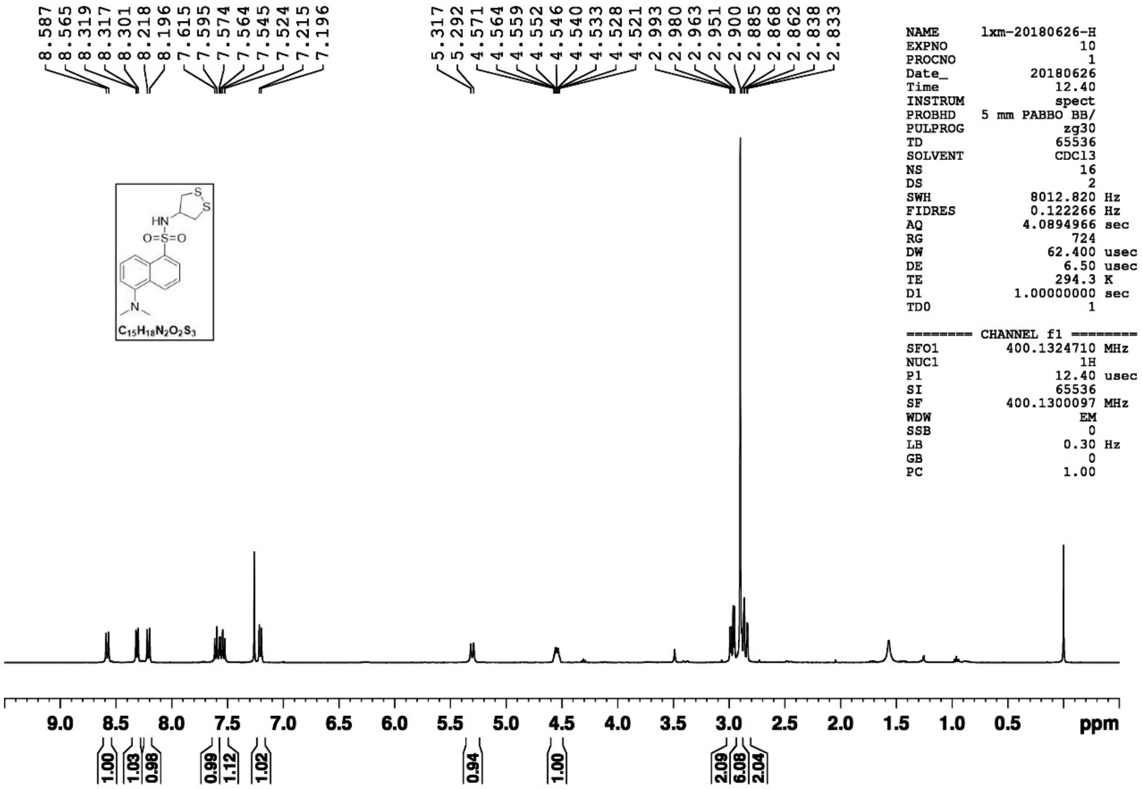

Supplementary Figure 71. ^{1}H NMR Spectra of **TRFS11** in $CDCl_3$ (400 MHz).

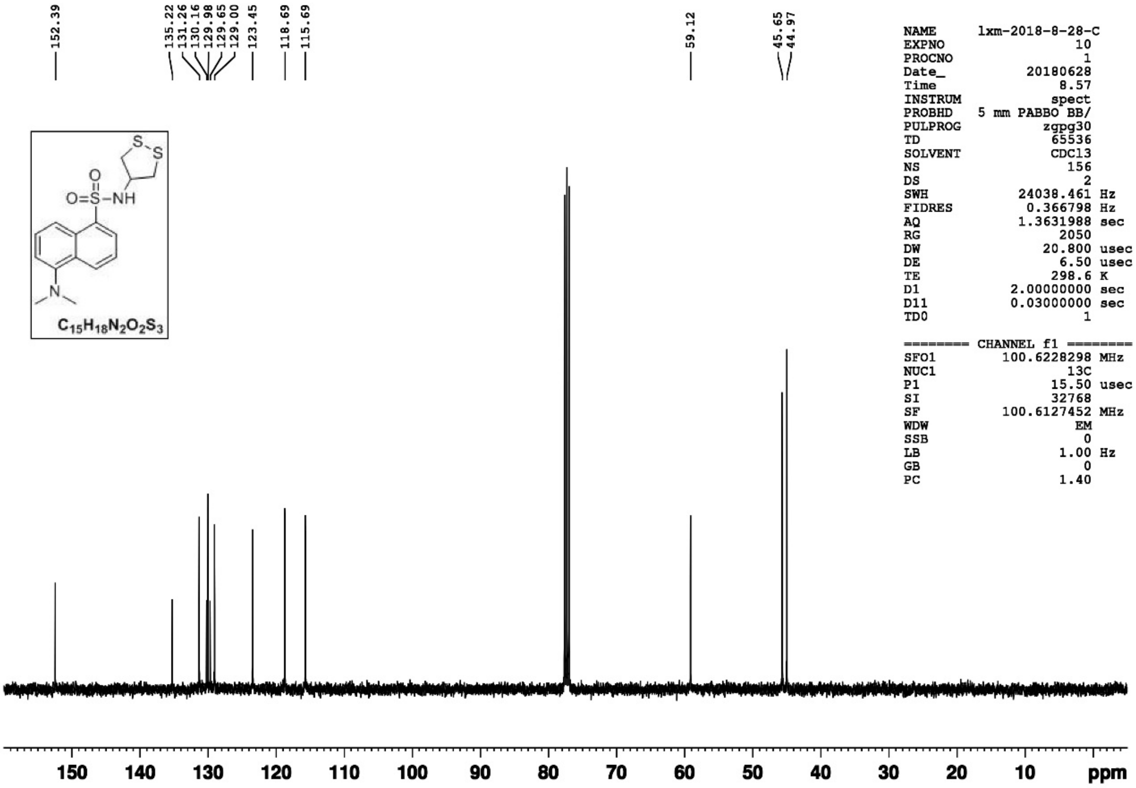

Supplementary Figure 72. ^{13}C NMR Spectra of **TRFS11** in $CDCl_3$ (100 MHz, $CDCl_3$)

Generic Display Report

Analysis Info

Analysis Name	D:\Data\Students_MS\New Folder\20180626-LXM-354.d	Acquisition Date	6/27/2018 11:15:45
Method	STUDENTS.m	Operator	ESQ6K
Sample Name	default	Instrument	esquire6000
Comment			

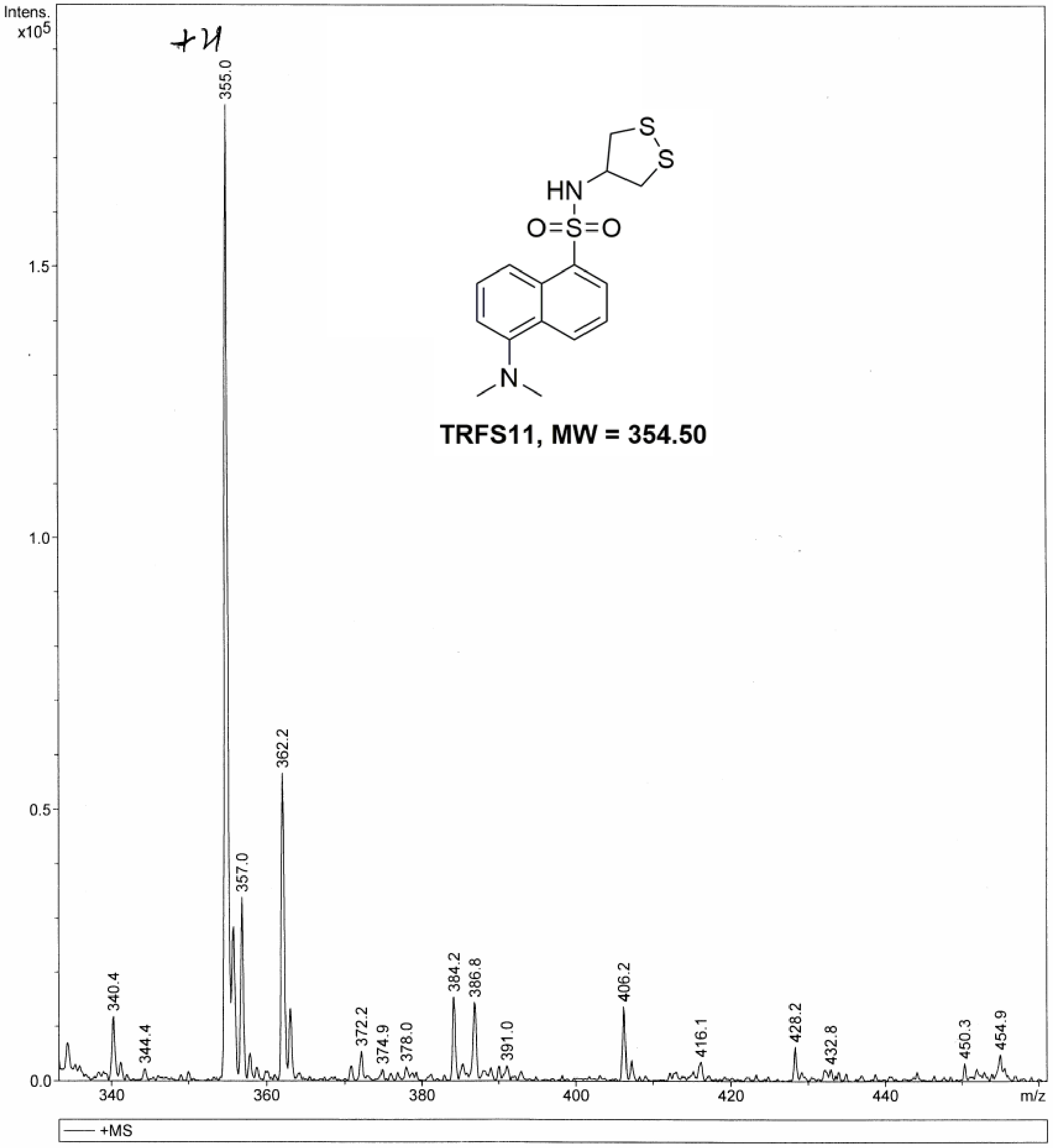

Bruker Daltonics DataAnalysis 3.4 printed: 6/27/2018 11:16:27 Page 1 of 1

Supplementary Figure 73. MS Spectra of **TRFS11** (ESI).

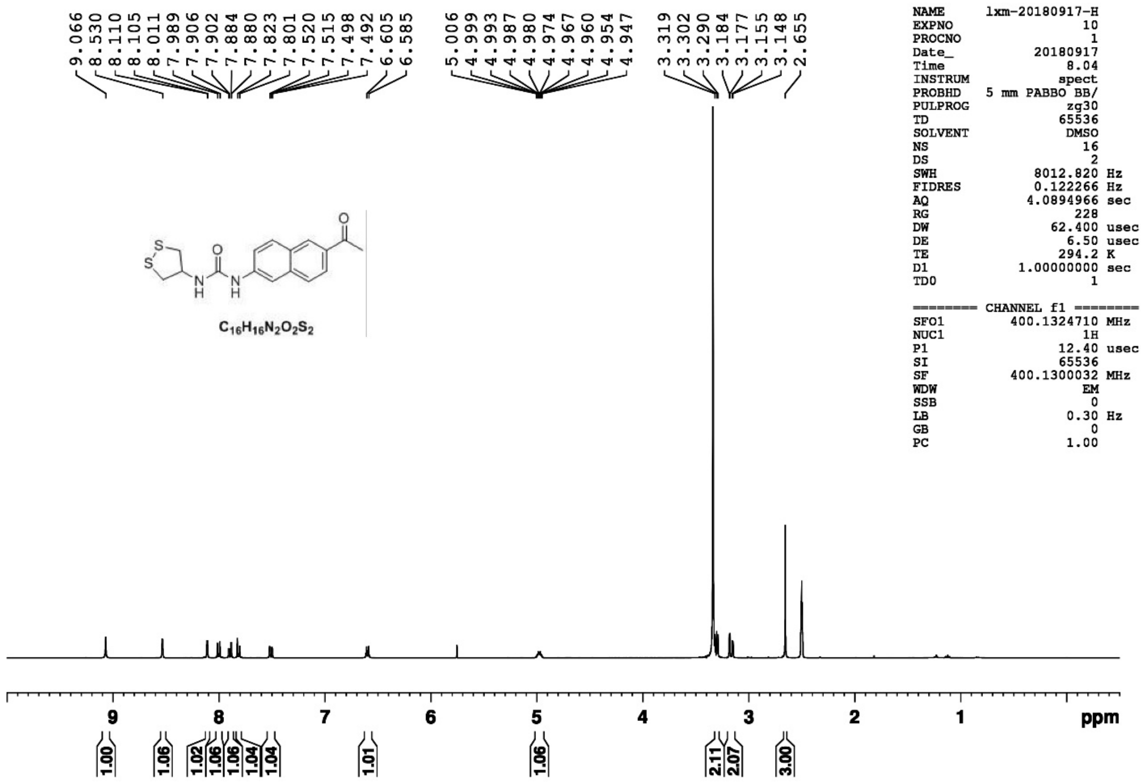

Supplementary Figure 74. ^{1}H NMR Spectra of **TRFS12** in DMSO-d_6 (400 MHz).

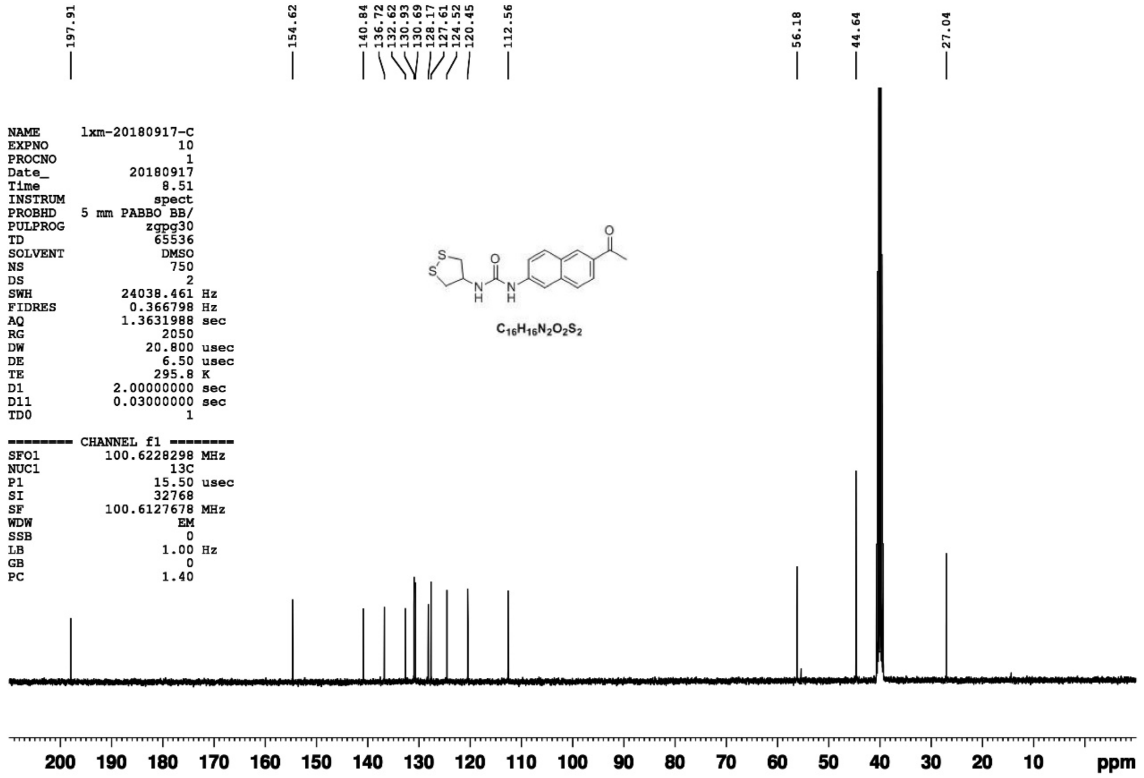

Supplementary Figure 75. ^{13}C NMR Spectra of **TRFS12** in DMSO-d_6 (100 MHz).

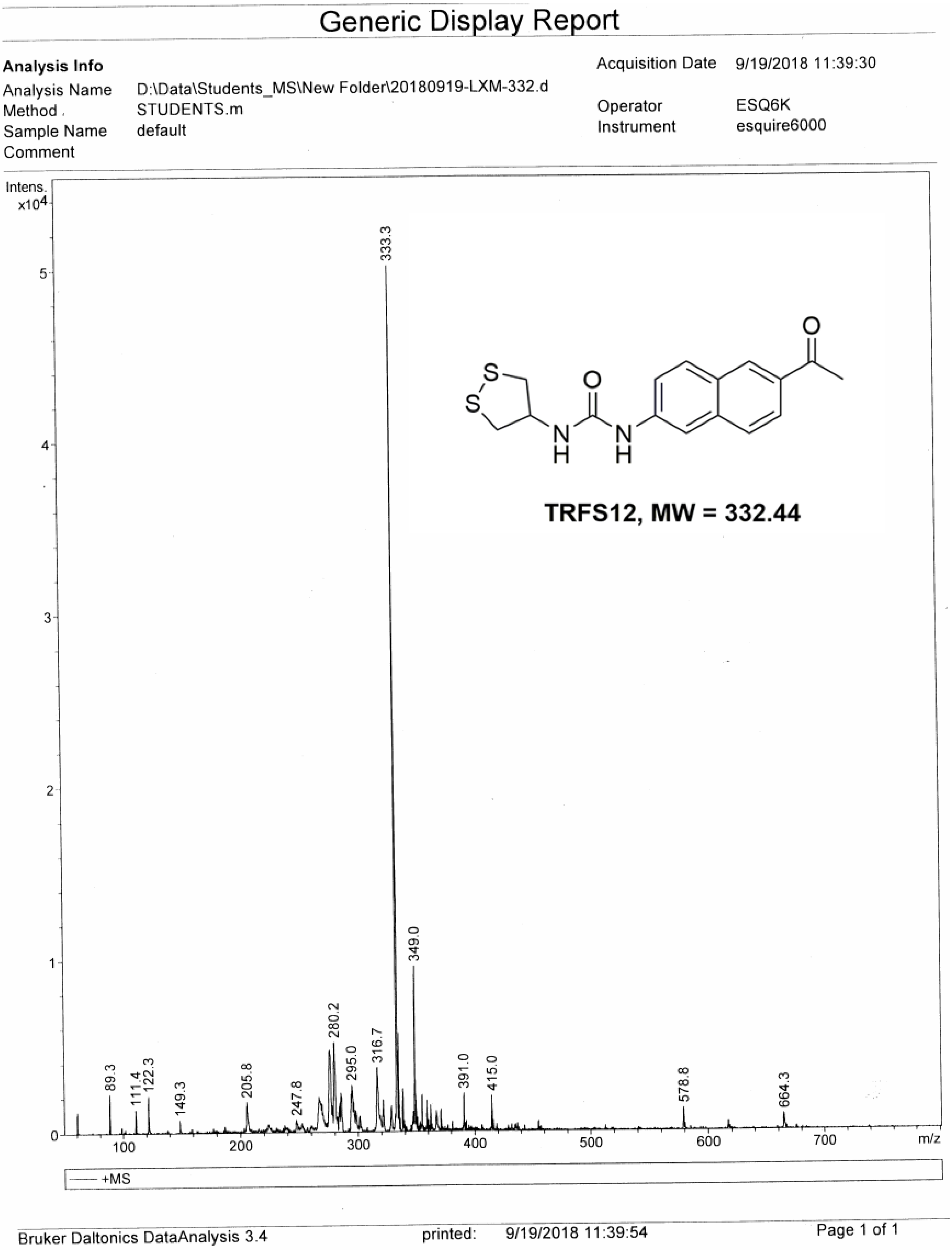

Supplementary Figure 76. MS Spectra of **TRFS12** (ESI).

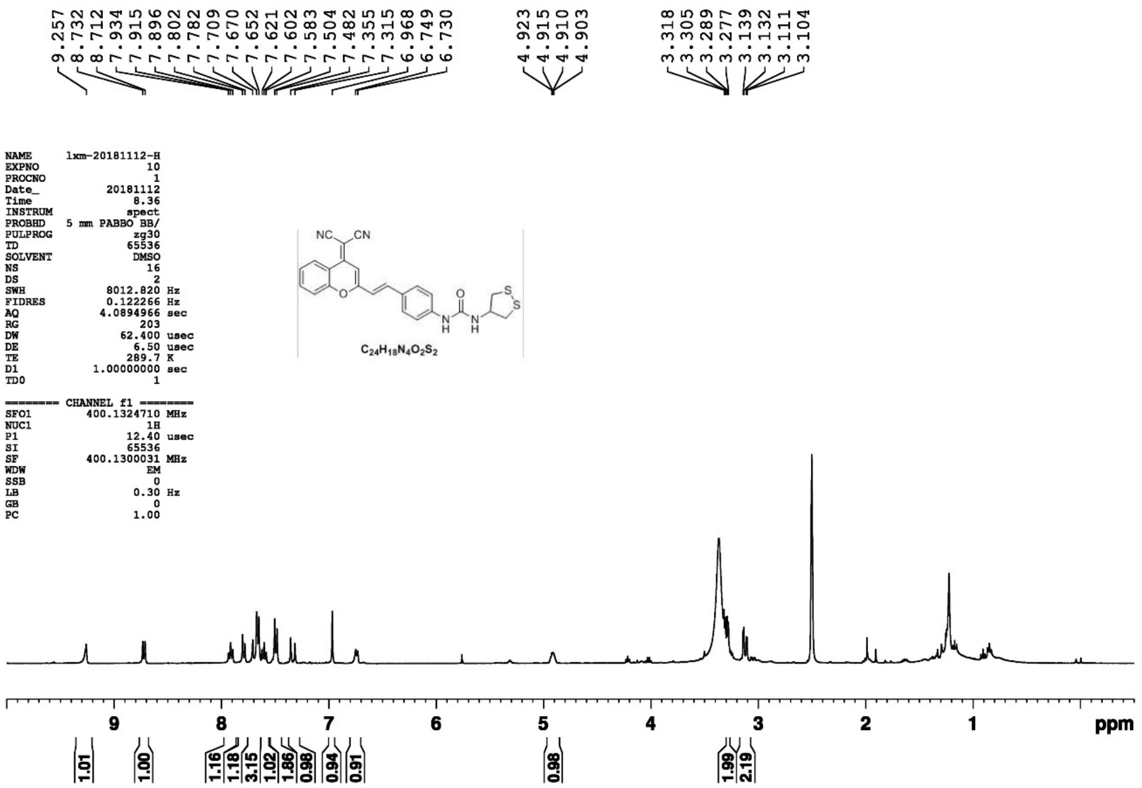

Supplementary Figure 77. ^{1}H NMR Spectra of **TRFS13** in DMSO-d_6 (400 MHz).

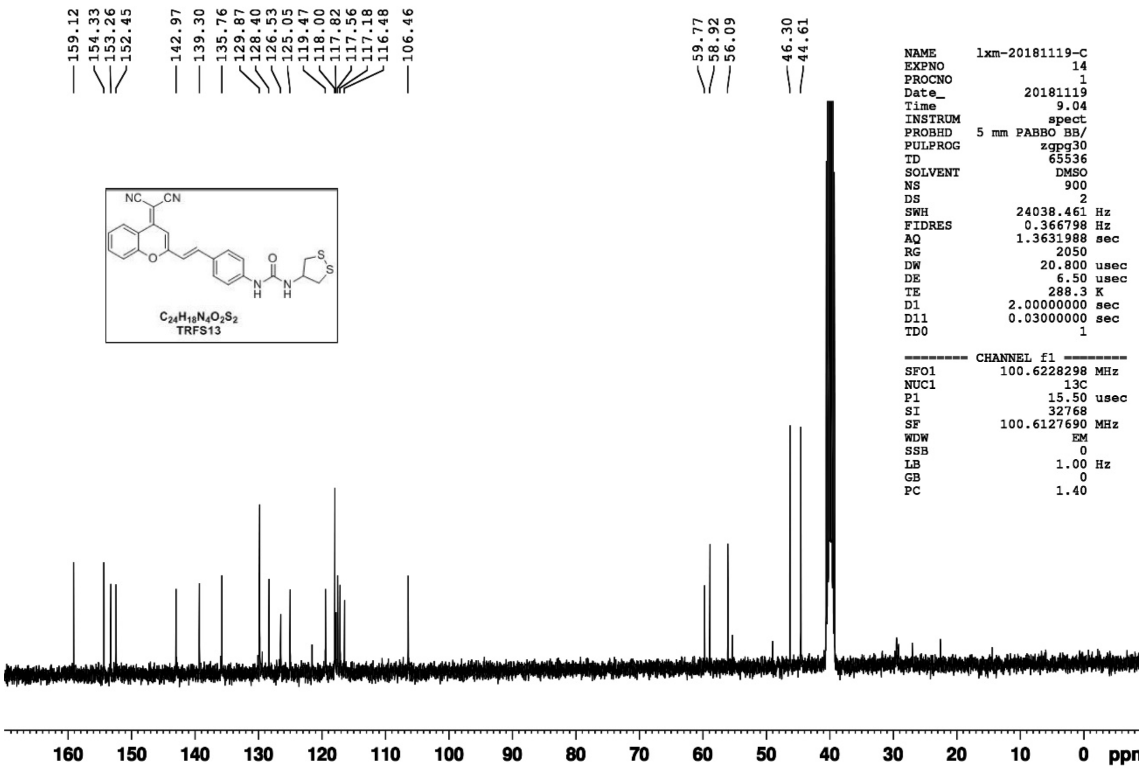

Supplementary Figure 78. ^{13}C NMR Spectra of **TRFS13** in DMSO-d_6 (100 MHz).

Generic Display Report

Analysis Info

Analysis Name	D:\Data\Students_MS\New Folder\LiXM-20171023-458.55--.d	Acquisition Date	10/23/2017 10:50:16
Method	STUDENTS.m	Operator	ESQ6K
Sample Name	default	Instrument	esquire6000
Comment			

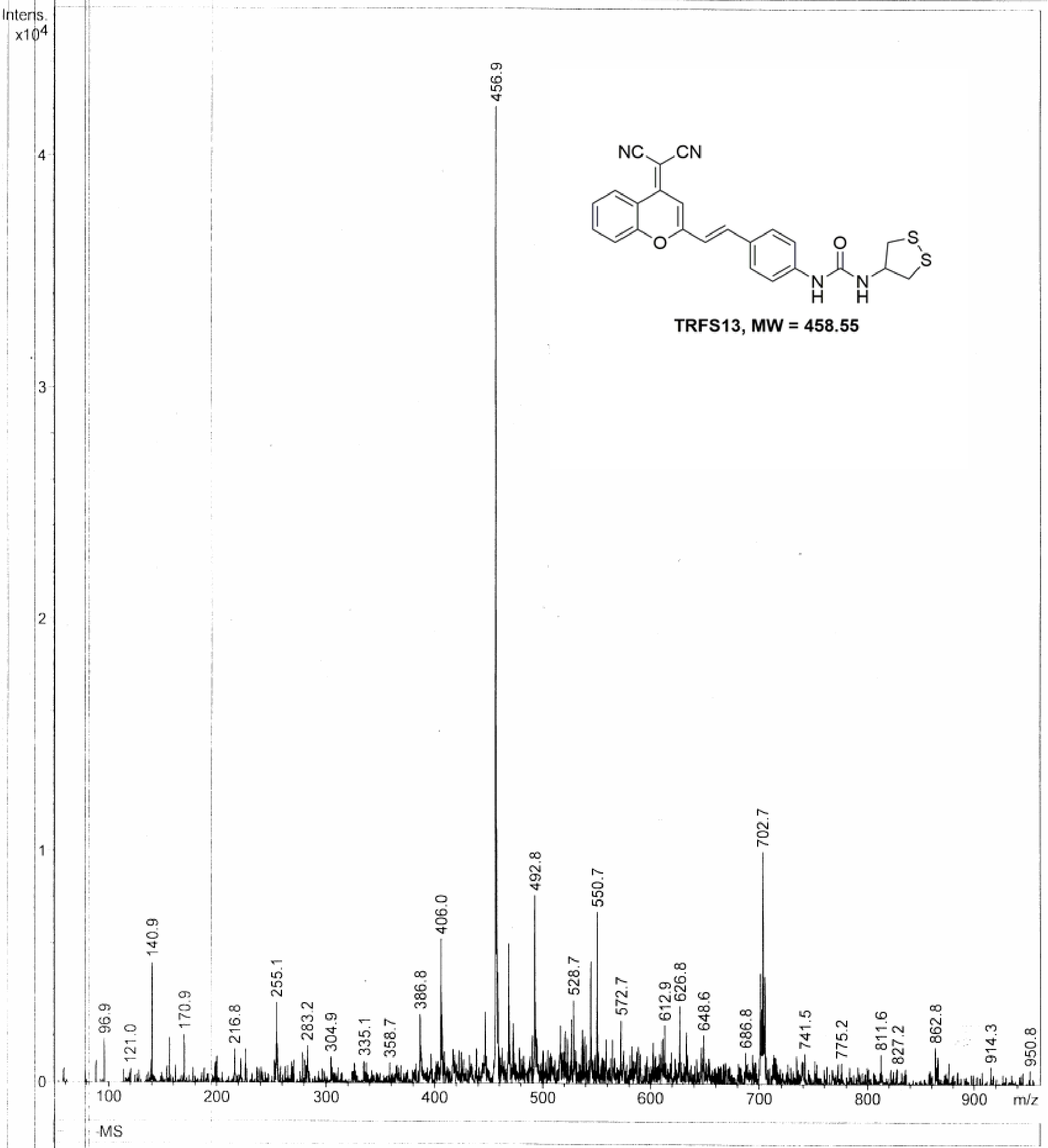

Bruker Daltonics DataAnalysis 3.4 printed: 10/23/2017 10:51:36 Page 1 of 1

Supplementary Figure 79. MS Spectra of **TRFS13** (ESI).

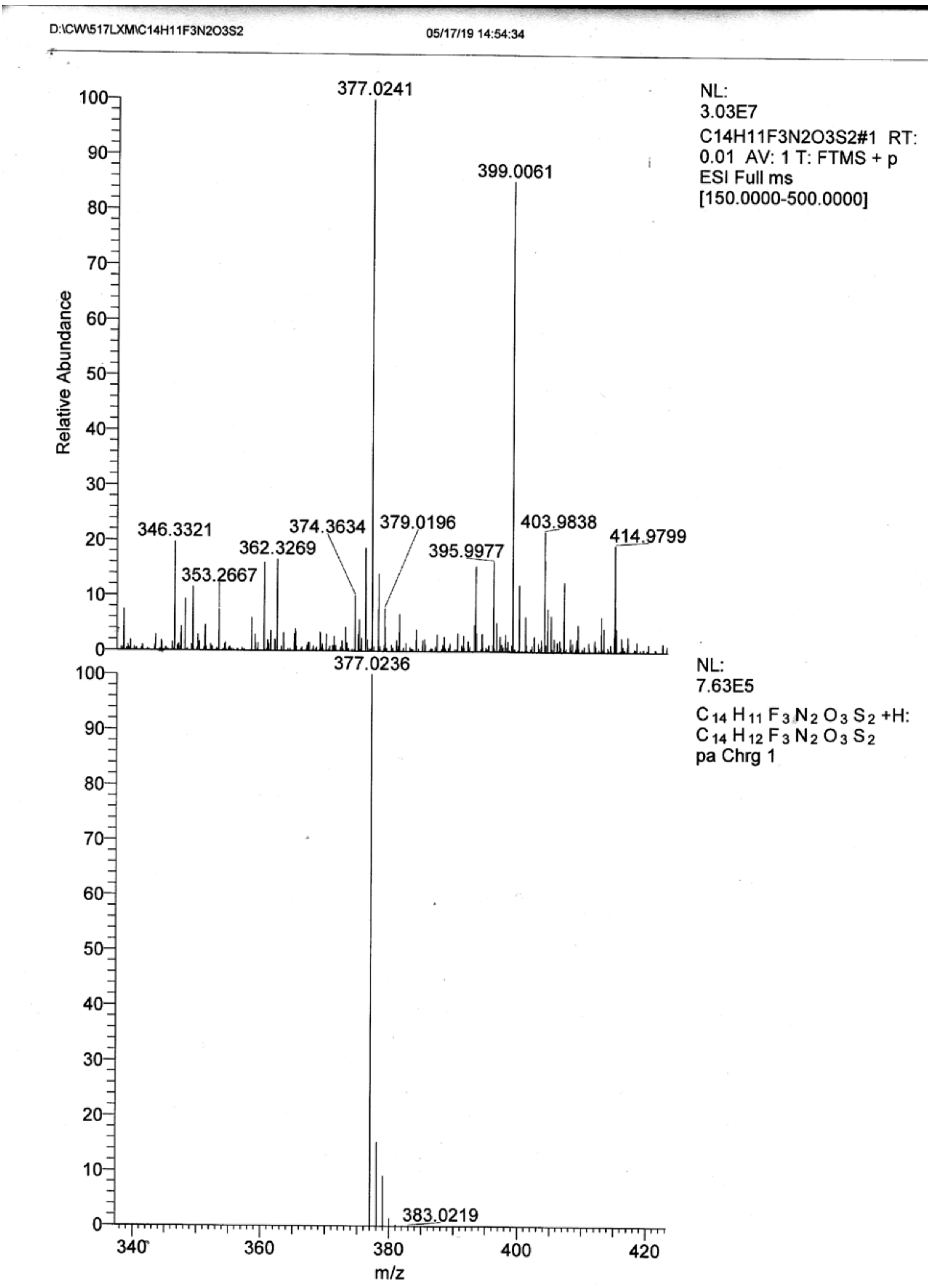

Supplementary Figure 80. HRMS Spectra of **TRFS9** (ESI).

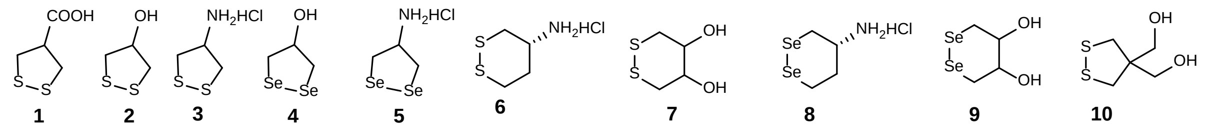

Supplementary Figure 81. Structures of cyclic diselenide/disulfide compounds.

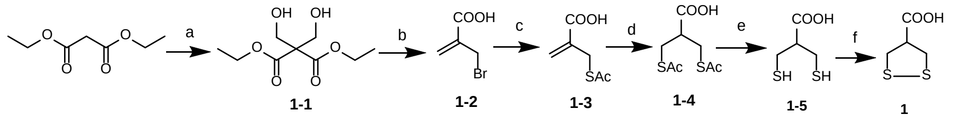

(a) K_2CO_3/ HCHO, 94%; (b) HBr, 40%; (c) AcSH/ Na_2CO_3, 92%; (d) AcSH, 50%; (e) $KHCO_3$; (f) I_2/ NaOAc/ $Na_2S_2O_3$, 43%.

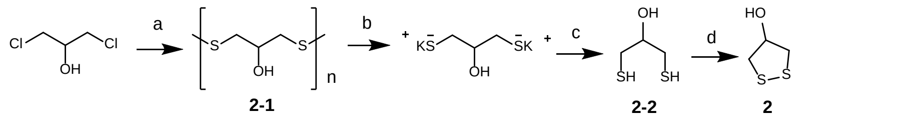

(a) $S_8/N_2H_4•H_2O/NH_2CH_2CH_2OH$, 93%; (b) $N_2H_4•H_2O$/KOH; (c) HCl, 0 °C, air, 86%; (d) $NaHCO_3/I_2/DCM/Na_2S_2O_3$.

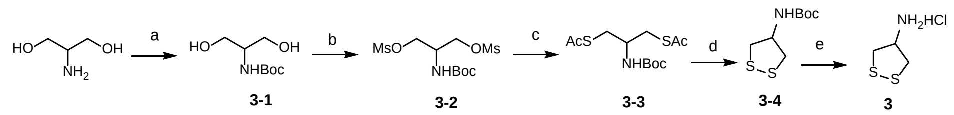

(a) Boc_2O/EtOH, 90%; (b) MsCl/TEA/DCM, 94%; (c) KSAc/DMF, 86%; (d) $NaOH/EtOH/H_2O/I_2/Na_2S_2O_3$, 76%; (e)HCl/ethyl acetate.

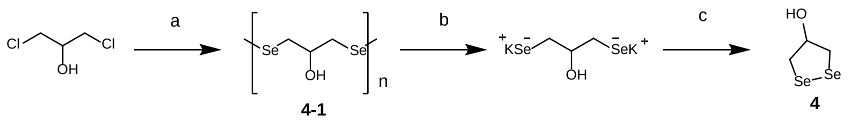

(a) $Se/N_2H_4•H_2O/NH_2CH_2CH_2OH$, 93%; (b) $N_2H_4•H_2O$/KOH; (c) HCl, 0 °C, air, 87%.

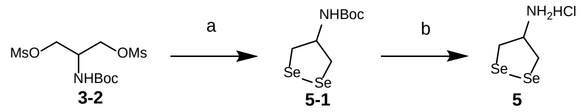

(a) Se/Na/naphthalene/THF, 27%; (b) HCl/ehtyl ecetate.

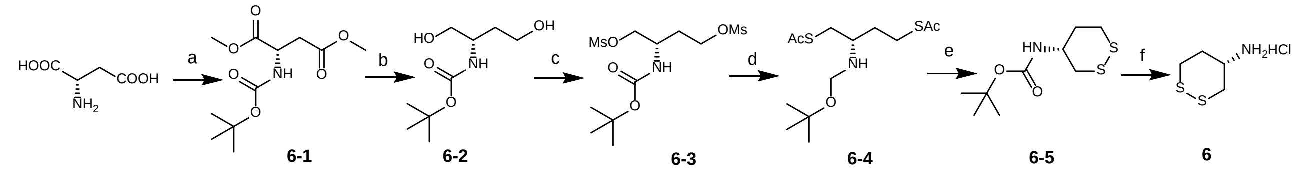

(a) 1)$SOCl_2$, DCM; 2) $NaHCO_3$, DCM, 90%; (b) $LiAlH_4$, THF, 78%; (c) MsCl, TEA,DCM, 87%; (d) KSAC, 18-crown-6, DMF, 82%; (e) KOH, MeOH, air, 84%; (f) HCl/ehtyl acetate.

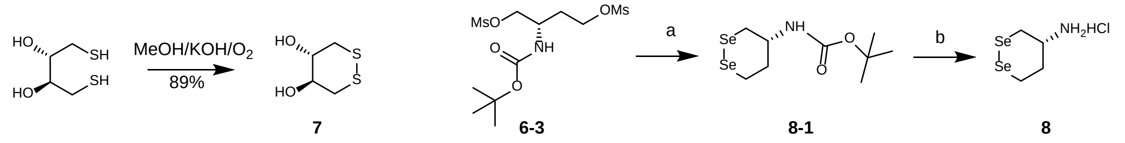

(a) Se/naphthalene/Na, THF, 45%; (b) Hcl/ehtyl ecetate

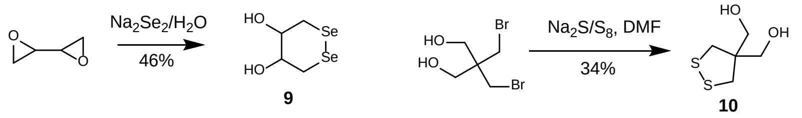

Supplementary Figure 82. Synthesis of compound **1-10**.

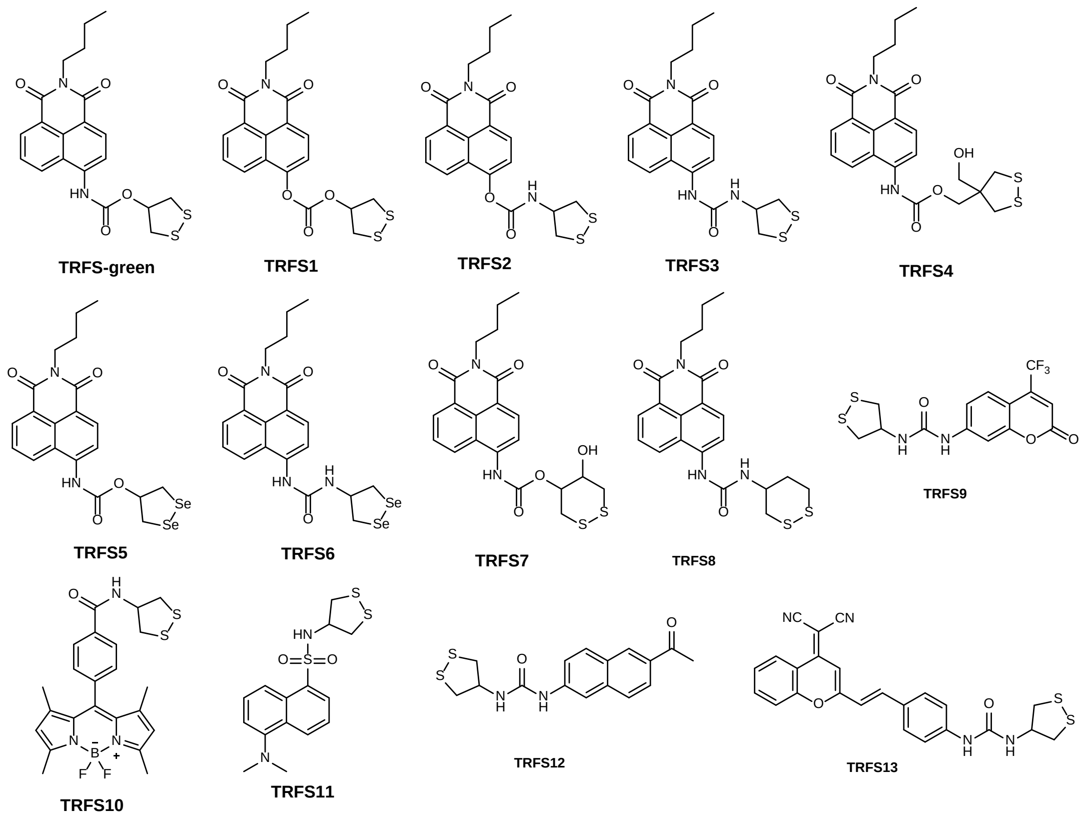

Supplementary Figure 83. Structures of TRFS series probes.

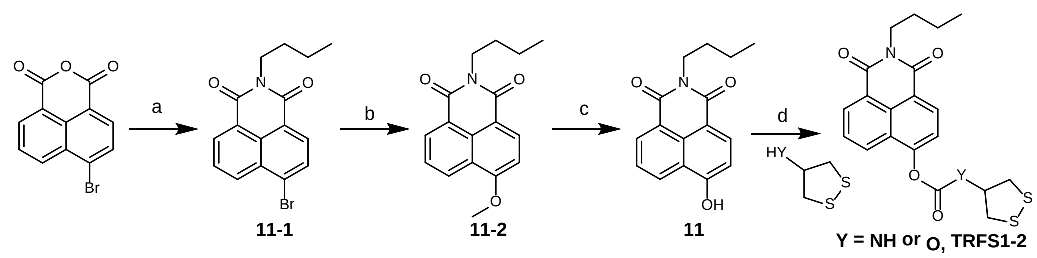

(a) n-Butylamine, EtOH, reflux, 85%; (b) CH_3ONa/CH_3OH, $CuSO_4$, 82%; (c) HI, reflux, 43%; (d) triphosgene, DMAP.

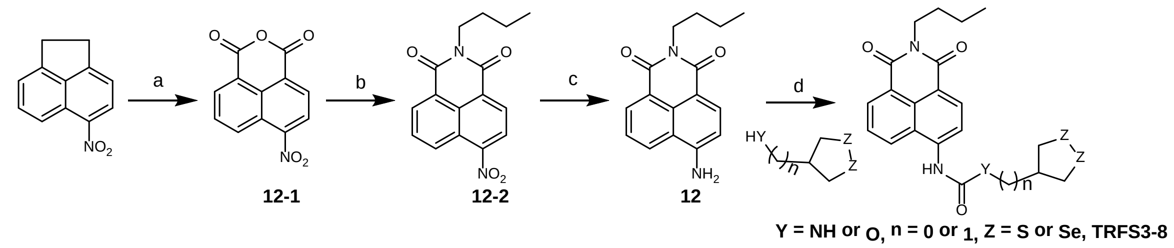

(a) $Na_2Cr_2O_7$/ AcOH,relux, 76%; (b) n-Butylamine/EtOH, reflux; 80%; (c) $SnCl_2$/HCl/EtOH, reflux, 78%; (d) triphosgene, DMAP

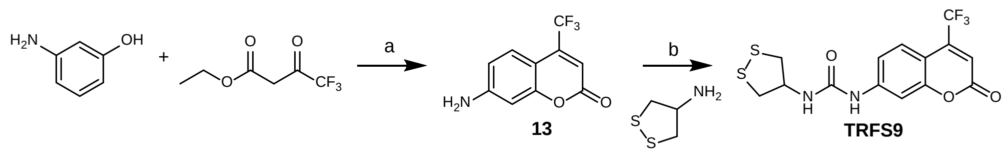

(a) ZnCl2, EtOH,reflux,45%; (b) triphosgene/DMAP/DCM, 72%.

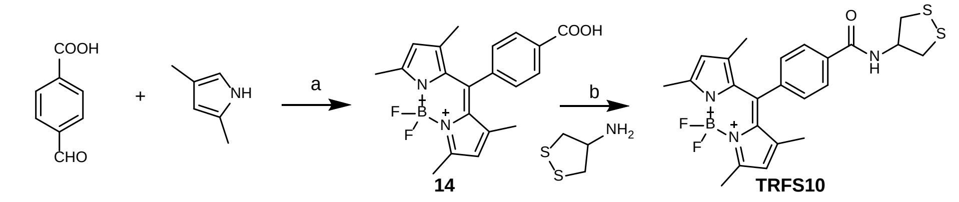

(a) 1) DCM, TFA; 2) DDQ; 3) TEA, $BF_3.OEt_2$; 25%; (b) DCM/TEA/EDC.HCl/HOBt, 75%.

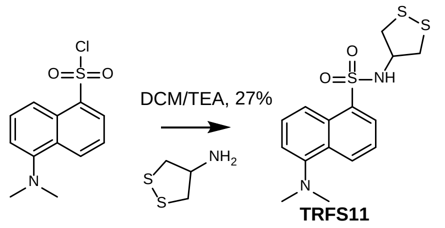

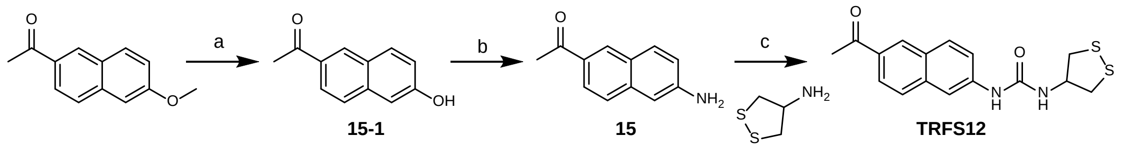

(a) HCl, reflux, 42%; (b) $Na_2S_2O_5$, NH_4OH, 35%; (c) triphosgene/DMAP/toluene, 66%.

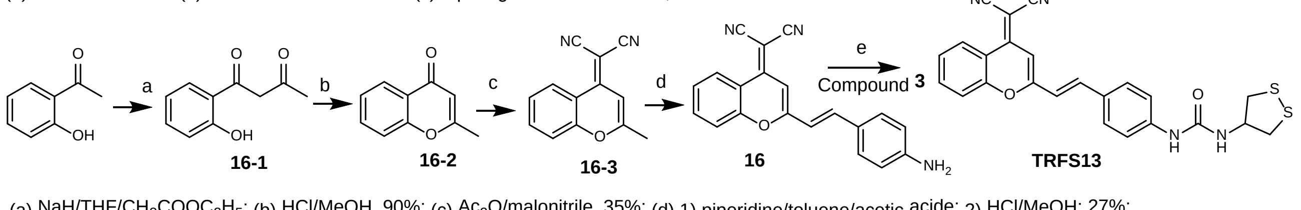

(a) NaH/THF/$CH_3COOC_2H_5$; (b) HCl/MeOH, 90%; (c) Ac_2O/malonitrile, 35%; (d) 1) piperidine/toluene/acetic acide; 2) HCl/MeOH; 27%;
(e) triphosgene/DMAP/toluene, 59%.

Supplementary Figure 84. Synthesis of TRFS series probes.

Supplementary Tables

Supplementary Table 1. The calculated excited states, excitation energy, excitation wavelength, oscillator strength, transition type and corresponding contribution of absorption spectrum of Fast-TRFS using optimized ground state structure.

Excited States	excitation energy/eV	excitation wavelength /nm	oscillator strength	transition type	contribution
**S^1**	3.3440	370.77	0.0004	**HOMO->LUMO+1**	**97.18%**
**S^2**	4.0989	302.48	0.6611	HOMO-2->LUMO	2.62%
				**HOMO-1->LUMO**	**89.37%**
				HOMO->LUMO	2.03%
**S^3**	4.7027	263.64	0.0362	HOMO-2->LUMO	74.94%
				HOMO-1->LUMO	2.07%
				HOMO-1->LUMO+2	15.87%

Supplementary Table 2. The calculated excited states, excitation energy, excitation wavelength, oscillator strength, transition type and corresponding contribution of absorption spectrum of R-Fast-TRFS using optimized ground state structure.

Excited States	excitation energy/eV	excitation wavelength /nm	oscillator strength	transition type	contribution
**S^1**	4.0927	302.94	0.6835	HOMO-3 ->LUMO	2.33%
				HOMO-1->LUMO	12.13%
				**HOMO->LUMO**	**79.84%**
**S^2**	4.7013	263.73	0.0367	HOMO-3 ->LUMO	75.14%
				HOMO-1->LUMO+1	2.12%
				HOMO->LUMO	2.17%
				HOMO->LUMO+1	14.19%

Supplementary Table 3. The calculated excited states, excitation energy, excitation wavelength, oscillator strength, transition type and corresponding contribution of fluorescence emission spectrum of Fast-TRFS using optimized $\mathbf{S^2}$ state structure.

Excited States	excitation energy/eV	excitation wavelength /nm	oscillator strength	transition type	contribution
$\mathbf{S^1}$	3.3297	372.36	0.0011	HOMO-1->LUMO+1	5.22%
				**HOMO->LUMO+1**	**93.02%**
$\mathbf{S^2}$	3.5518	349.07	0.6753	**HOMO-1->LUMO**	**88.51%**
				HOMO->LUMO	6.31%
$\mathbf{S^3}$	4.3500	285.02	0.0258	HOMO-2->LUMO	88.10%
				HOMO-1->LUMO+2	4.43%
				HOMO-1->LUMO+3	3.12%

Supplementary Table 4. The calculated excited states, excitation energy, excitation wavelength, oscillator strength, transition type and corresponding contribution of fluorescence emission spectrum of R-Fast-TRFS using optimized $\mathbf{S^1}$ state structure.

Excited States	excitation energy/eV	excitation wavelength /nm	oscillator strength	transition type	contribution
$\mathbf{S^1}$	3.5432	349.92	0.6959	HOMO-1->LUMO	3.61%
				**HOMO->LUMO**	**91.55%**
$\mathbf{S^2}$	4.3463	285.26	0.0267	HOMO-3->LUMO	88.28%
				HOMO->LUMO+1	4.49%
				HOMO->LUMO+2	3.22%

Supplementary Table 5. Cartesian Coordinates of Fast-TRFS (ground state)

C	-0.11398804	0.19752205	-0.30139488
C	-0.92187092	1.33791399	-0.22548642
C	-2.29798427	1.20429174	-0.09723082
C	-2.92210446	-0.05294571	-0.03833472
C	-2.09105071	-1.18661292	-0.11494508
C	-0.71971602	-1.07758044	-0.24388829
H	-0.49574873	2.34167071	-0.26529962
C	-4.36465969	-0.05805957	0.09417369
H	-2.52793823	-2.18426851	-0.07350868
H	-0.09746903	-1.96549839	-0.30381927
C	-5.05338021	1.10059642	0.15584311
C	-4.37384930	2.39242963	0.09204966
H	-6.13707520	1.13247707	0.25502488
C	-5.11128874	-1.37437386	0.16364330
F	-4.70217269	-2.10168072	1.20771778
F	-4.90062176	-2.10203536	-0.93754035
F	-6.42459292	-1.20285741	0.28520817
O	-3.01593993	2.35514024	-0.03155477
O	-4.91284212	3.46681161	0.13983578
N	1.25513982	0.39414983	-0.42963319
C	2.25560509	-0.56129816	-0.52470252
H	1.54646902	1.36359411	-0.46538419
O	2.05205805	-1.76462381	-0.52788010
N	3.50506739	-0.02761610	-0.63429391
H	3.66496157	0.96261597	-0.47636288
C	4.67365690	-0.87631671	-0.65356785
C	5.84903651	-0.13937058	-1.28702400
C	5.11416917	-1.32691481	0.74367880
H	4.43555410	-1.77253997	-1.24514171
H	5.57270433	0.32880473	-2.24217906
H	6.68875767	-0.82792479	-1.45013187
H	5.83978787	-2.14809726	0.66595114
H	4.25397050	-1.66472428	1.33736822
S	6.34008992	1.20195477	-0.15041262
S	5.88862272	0.08510805	1.61868832

Supplementary Table 6. Cartesian Coordinates of R-Fast-TRFS (ground state)

C	0.34967684	0.54997809	0.00069280
C	1.30679348	1.56907833	0.07064743
C	2.65861497	1.25243334	0.06144476
C	3.11245268	-0.07479770	-0.01558055
C	2.13387824	-1.08447083	-0.08457136
C	0.78339294	-0.79259318	-0.07768889
H	1.01711985	2.61909012	0.13353180
C	4.54733899	-0.27302399	-0.01664510
H	2.43567164	-2.12998376	-0.14634603
H	0.04576132	-1.58751914	-0.13179444
C	5.38694417	0.78101057	0.05294723
C	4.88255589	2.14985496	0.13221015
H	6.46968670	0.66741361	0.05426973
C	5.11522105	-1.67528083	-0.09574715
F	4.71628312	-2.29064436	-1.21317244
F	4.70377527	-2.41653982	0.93754996
F	6.44523035	-1.68142814	-0.08847850
O	3.52633925	2.29484716	0.13112276
O	5.56193266	3.14052707	0.19702332
N	-0.98637864	0.92959343	0.01115066
C	-2.11165389	0.11695950	-0.02680991
H	-1.14433458	1.92902779	0.06137555
O	-2.06336253	-1.10102302	-0.07784320
N	-3.28094570	0.81758116	0.00005955
H	-3.26528163	1.83021287	0.02684523
C	-4.57712175	0.16403895	-0.03563404
C	-5.37603333	0.49186288	1.24050594
C	-5.26819131	0.56552417	-1.34718502
H	-4.36226051	-0.91353556	-0.05093696
H	-5.97013147	1.40959596	1.11156454
H	-4.66220172	0.68041130	2.05408784
H	-4.59761704	0.33852711	-2.18733139
H	-5.44558728	1.65288399	-1.35531248
S	-6.43723681	-0.84043507	1.88902625
S	-6.90469040	-0.16265761	-1.67841824
H	-7.32212516	-0.84899979	0.87118435
H	-6.50668265	-1.44860801	-1.76636938

Supplementary Table 7. Cartesian Coordinates of Fast-TRFS (S^2 state)

C	-0.11076842	0.18384761	-0.27891052
C	-0.92557574	1.34326919	-0.20545126
C	-2.28782986	1.23672059	-0.08666884
C	-2.94370134	-0.05729877	-0.03158517
C	-2.09172265	-1.21002147	-0.10470655
C	-0.73123674	-1.10533562	-0.22353959
H	-0.48251351	2.33975949	-0.24240387
C	-4.35315586	-0.07995677	0.08744696
H	-2.53819892	-2.20248354	-0.06536650
H	-0.10654002	-1.99238683	-0.27873519
C	-5.04975788	1.15104792	0.14351694
C	-4.38894645	2.40680329	0.09064293
H	-6.13488038	1.18090084	0.23197550
C	-5.09749667	-1.37372105	0.15159686
F	-4.71560121	-2.13311051	1.19673537
F	-4.89997647	-2.13369718	-0.94307747
F	-6.41401170	-1.18950230	0.26495282
O	-2.98775012	2.38495363	-0.02297059
O	-4.88516996	3.51644318	0.13191710
N	1.23533510	0.37033183	-0.39952448
C	2.25061796	-0.59588240	-0.49156497
H	1.53279085	1.34112244	-0.42837457
O	2.03489047	-1.79337973	-0.49437813
N	3.48875587	-0.05214420	-0.59323919
H	3.64290530	0.94427031	-0.46608393
C	4.66705286	-0.89002135	-0.63622472
C	5.81625652	-0.14356646	-1.30529824
C	5.14274406	-1.31938760	0.75557542
H	4.42144507	-1.79335994	-1.21365929
H	5.51378245	0.30454149	-2.26222592
H	6.66264357	-0.82236434	-1.47490316
H	5.86966453	-2.13826522	0.66798515
H	4.30011042	-1.65486012	1.37531914
S	6.30523969	1.22137732	-0.19837402
S	5.93487382	0.10546533	1.59417397

Supplementary Table 8. Cartesian Coordinates of R-Fast-TRFS (S^1 state)

C	-0.34121161	0.55743482	-0.00855100
C	-1.31332639	1.58441535	0.11482664
C	-2.65236656	1.28850356	0.10533214
C	-3.12437162	-0.07806169	-0.02951145
C	-2.11699952	-1.09346624	-0.15117421
C	-0.77945201	-0.79931895	-0.14225367
H	-1.01189614	2.62751870	0.22128746
C	-4.52150478	-0.29865911	-0.03002595
H	-2.42252592	-2.13362015	-0.25451979
H	-0.03541880	-1.58501571	-0.23733066
C	-5.38451345	0.81663517	0.10031192
C	-4.90289376	2.14548929	0.23194452
H	-6.46665536	0.69386182	0.10486067
C	-5.08178552	-1.67690788	-0.16251523
F	-4.67397536	-2.49371764	0.82841715
F	-4.70365317	-2.27689017	-1.30805288
F	-6.41590589	-1.67937942	-0.14426698
O	-3.50765970	2.32119404	0.22761873
O	-5.55034188	3.16851151	0.34961213
N	0.97077099	0.93046457	0.00987522
C	2.11497463	0.11810812	-0.07862840
H	1.13023195	1.92993663	0.09809133
O	2.06153007	-1.08854512	-0.22387179
N	3.27075671	0.82454851	0.00205215
H	3.25204658	1.81371451	0.22502095
C	4.56655893	0.16136772	-0.01698070
C	5.55318007	0.98524806	-0.86236861
C	5.00305450	-0.07746025	1.43750265
H	4.40010241	-0.80902780	-0.50512803
H	6.08359067	1.72447639	-0.24316757
H	4.98350494	1.54401113	-1.61778708
H	4.20681275	-0.62257581	1.96313604
H	5.13199238	0.88960162	1.94799672
S	6.75476502	0.02831496	-1.84085415
S	6.58215015	-0.94832384	1.69066119
H	7.44959676	-0.46681331	-0.79601847
H	6.21480578	-2.13038368	1.15395406

Supplementary Methods

Synthesis of compound 1. The compound was synthesized according to Supplementary Figure 82. Compound **1-1** was prepared according to the following procedures. Formaldehyde solution (81 mL, 37%) and K_2CO_3 (1 g, 7.2 mmol) were placed in a 250-mL round-bottom flask, and then diethyl malonate (80 g, 0.5 mol) was added dropwise within 40 min. at such a rate, the temperature of the reaction mixture was held at 30 ^oC. The reaction was continued with stirring for 1 h. A saturated solution of ammonium sulfate (160 mL) was added and the mixture was extracted with 320 mL of ether. The ethereal extract was dried for 1 h with Na_2SO_4 and the solvent was removed under reduced pressure to afford white solid. The residue was used in the next step without purification. Compound **1-2** was prepared was prepared according to the following procedures. Compound **1-1** (50 g, 0.23 mol) and hydrobromic acid (48%, 104 mL, 0.91 mol) was heated to reflux at 120 ^oC for 14 h. After the starting material was consumed, the excess hydrobromic acid was removed under reduced pressure to give a light-orange solid. The crude product was redissolved in dichloromethane (DCM, 50 mL), washed with water and dried over Na_2SO_4, filtered, and concentrated to give white compound **1-2** (35.2 g, 94% yield)[1]. ^{1}H NMR (400 MHz, DMSO-d_6) δ 12.97 (br, 1H), 6.18 (s, 1H), 6.06 (s, 1H), 4.27 (s, 2H); ^{13}C NMR(100 MHz, DMSO-d_6) δ 166.0, 138.2, 129.2, 30.8; ESI-MS (m/z): $[M-H]^-$ 165.1. Compound **1-3** was prepared according to the following procedures. To a stirred suspension of compound **1-2** (4.95 g, 30.0 mmol) in 100 mL of water in an ice bath was added a solution of sodium carbonate (5.50 g, 52.0 mmol) in 20 mL of water in small portions. To the resulting solution was added thiolacetic acid (2.20 mL, 30.5 mmol), and the solution was stirred in an ice bath for 15 min. The solution was acidified with HCl to pH=1 and extracted with ethyl acetate (2 × 50 mL). The organic layer was dried by $MgSO_4$ and concentrated at reduced pressure to yield compound **1-3** as a white solid (1.9 g, 40% yield). ^{1}H NMR (400 MHz, $CDCl_3$) δ 11.94 (br, 1H), 6.03 (s, 1H), 3.77 (s, 2H), 2.34 (s, 2H); ^{13}C NMR (100 MHz, $CDCl_3$) δ 194.9, 1714, 135.6, 130.5, 30.3, 29.4; ESI-MS (m/z): $[M+Na]^+$ 182.9. Compound **1-4** was prepared according to the following procedures. To the compound **1-3** was added thiolacetic acid (3.25 mL, 45.0 mmol). After 15 h at room temperature, the reaction mixture was concentrated at reduced pressure to remove the excess thiolacetic acid. The residue was used in the next step without further purification. Compound **1-5**

was prepared according to the following procedures. The compound **1-4** was added to 200 mL of deoxygenated 10% (wt) aqueous $KHCO_3$ solution and then the mixture was refluxed for 5 h under argon. The reaction mixture was cooled in an ice bath, acidified to pH=1 with HCl, and extracted with ethyl acetate (200 mL). The organic layer was dried with Na_2SO_4 and concentrated at reduced pressure to yield an oily residue which was recrystallized in hexane (by cooling to -80 ^{o}C) to yield a white crystalline solid (3.43 g, 75% yield). This compound was used in the next step without further purification[2]. Compound **1** was prepared according to the following procedures. Compound **1-5** (152 mg, 1 mmol) was added to a stirred solution of KOH (134 mg, 2.4 mmol) 10% w/w in H_2O in a 50 mL flask in MeOH (50 mL). Once dissolved, air was bubbled through the reaction mixture for 24 h. The solution was then acidified to pH = 1 using 1 M HCl and concentrated under reduced pressure. The residue was dissolved in 100 mL of ethyl acetate and washed with brine (2×50 mL), dried over Na_2SO_4 and evaporated to dryness in vacuo. The crude product was purified by silica gel chromatography (DCM/MeOH = 50/1) to give compound **1** as a yellow solid (105 mg, 89% yield). ^{1}H NMR (400 MHz, $CDCl_3$) δ 3.54-3.47 (m, 3H), 3.38-3.31 (m, 2H); ^{13}C NMR (100 MHz, DMSO-d6) δ 173.4, 50.7, 41.5; ESI-MS (m/z): $[M-H]^-$ 149.3.

Synthesis of compound 2. The compound was synthesized according to Supplementary Figure 82. Compound **2-2** was prepared according to the following procedures. This compound was synthesized according to the literature[3]. Elemental sulfur (9.6 g, 300 mmol) was dissolved in a mixture of 45 mL of hydrazine hydrate and 5.5 g of ethanolamine. The mixture was heated to 65 ^{o}C for a while and then cooled to room temperature. 1,3-Dichloro-2-propanol (19.35 g, 150 mmol) was added to the resulting solution dropwise, and the mixture was allowed to stir for another 2.5 h to obtain oligomer **2-1**. Then the oligomer **2-1** (10 g, 82 mmol) was dissolved in a mixture of 102 mL of hydrazine hydrate and 23 g of KOH. The mixture was stirred at 80 ^{o}C for 2.5 h. The solution was cooled to room temperature, poured into a mixture of ice with 240 mL of concentrated hydrochloric acid, and extracted with DCM. The organic phase was dried over Na_2SO_4 and evaporated to dryness in vacuo. The crude product was purified by silica gel chromatography (petroleum ether/ethyl acetate: 4/1) to give compound **2-2** as a pale yellow oil (12.4 g, 86% yield). ^{1}H NMR (400 MHz, $CDCl_3$) δ 3.75-3.67 (m, 1H), 3.50 (d, J = 5.0 Hz, 1H), 2.81-2.67 (m, 4H), 1.47 (m, 2H); ^{13}C NMR (100 MHz, $CDCl_3$) δ 72.9, 29.8; EI-MS m/z (%): 124 (M^+, 16), 122 (53), 77 (26), 59 (42), 44 (100). Compound **2** was

synthesized according to our previous work[4]. ^{1}H NMR (400 MHz, DMSO-d_6) δ 5.28 (d, J = 4.5 Hz, 1H), 4.80−4.74 (m, 1H), 3.17 (dd, J_1 = 11.6 Hz, J_2 = 5.0 Hz, 2H), 2.98 (dd, J_1 = 11.6 Hz, J_2 = 3.2 Hz, 2H); ^{13}C NMR (100 MHz, DMSO-d_6) δ 75.47, 46.24. EI-MS m/z (%): 122 (M^+, 100), 106 (13), 78 (40), 58 (39), 47 (38).

Like many other cyclic disulfide compounds, for example, lipoic acid, this compound upon standing, even in the refrigerator, polymerizes to a gummy material through disulfide exchange. So compound **2** is better to prepare fresh in a DCM solution prior to use[5].

Synthesis of compound 3. The compound was synthesized according to Supplementary Figure 82. Compound **3-1** was prepared according to the following procedures. 2-Amino-1,3-propanediol (1g, 11 mmol) was dissolved in 40 mL of ethanol in ice bath. To the solution was added dropwise of Boc_2O (2.4 g, 11 mmol) in 10 mL of ethanol and the mixture was stirred at room temperature for 4 h. The solvent was removed in vacuo to give compound **3-1** as a white solid (1.9 g, 90% yield), which was used directly in the next step. ^{1}H NMR (400 MHz, $CDCl_3$) δ 5.30 (d, J = 6.8 Hz, 1H), 3.84-3.68 (m, 5H), 2.73 (s, 2H), 1.45 (s, 9H); ^{13}C NMR (100 MHz, $CDCl_3$) δ 156.4, 80.0, 63.5, 53.0, 28.4; ESI-MS (m/z): $[M+Na]^+$ 214.1. Compound **3-2** was prepared according to the following procedures. Methane sulfonyl chloride (1.76 mL, 23 mmol) was added dropwise at 0 ^{o}C to a stirred solution of compound **3-1** (1.91 g, 10 mmol) and trimethylamine (4.18 mL) in DCM (50 mL). The mixture was stirred at room temperature for 3 h. Then, the mixture was washed with an aqueous solution of $NaHCO_3$, dried with Na_2SO_4 and concentrated to dryness under vacuum. Chromatography of the residue on silica gel eluting with petroleum ether/ethyl acetate (1/1) gave compound **3-2** (3.3 g, 94% yield). ^{1}H NMR (400 MHz, $CDCl_3$) δ 5.12 (d, J = 8.4 Hz, 1H), 4.37-4.24 (m, 5H), 3.08 (s, 6H), 1.45 (s, 9H); ^{13}C NMR (100 MHz, $CDCl_3$) δ 154.8, 80.7, 66.9, 48.4, 37.4, 28.2; ESI-MS (m/z): $[M+Na]^+$ 370.1. Compound **3-3** was prepared according to the following procedures. Compound **3-2** (694 mg, 2 mmol) was dissolved in 20 mL of dimethylformamide (DMF). Potassium thioacetate (912 mg, 8 mmol) in 10 mL of DMF was added dropwise. The reaction mixture was stirred overnight at room temperature and then poured into 100 mL of brine and extracted with ethyl acetate (3 × 50 mL). The organic layer was washed with water (3×100 mL), dried over Na_2SO_4 and evaporated under vacuum. Chromatography of the residue on silica gel with a mixture of petroleum ether/ethyl acetate (4/1) gave compound **3-3** (528 mg, 86% yield). ^{1}H NMR (400 MHz, $CDCl_3$) δ 4.79 (s, 1H), 3.86 (s, 1H),

3.06 (s, 4H), 2.33 (s, 6H), 1.40 (s, 9H); ^{13}C NMR (100 MHz, $CDCl_3$) δ 195.4, 155.2, 79.6, 50.8, 32.5, 30.5, 28.2; ESI-MS (m/z): $[M+Na]^+$ 330.1. Compound **3-4** was prepared according to the following procedures. A solution of compound **3-3** (615 mg, 2 mmol) in 20 mL of ethanol was added with an aqueous solution of 1 M NaOH (10 mL) at room temperature for 1 h. The mixture was diluted with DCM (100 mL) and then an aqueous solution of 0.1 M iodine (45 mL) was added dropwise. The reaction mixture was stirred for 2 h at room temperature and an aqueous solution of 1 M $Na_2S_2O_3$ (10 mL) was added to the reaction mixture. The organic phase was separated, washed with water (3 × 100 mL), dried with Na_2SO_4, and evaporated under vacuum. Chromatography of the residue on silica gel with a mixture of petroleum ether/ethyl acetate (10/1) gave compound **3-4** as a yellow solid (336 mg, 76% yield). ^{1}H NMR (400 MHz, $CDCl_3$) δ 5.07 (s, 1H), 4.95 (m, 1H), 3.22 (dd, J_1 = 11.2 Hz, J_2 = 4.8 Hz, 4H), 3.09 (d, J = 11.2 Hz, 2H), 1.44 (s, 9H); ^{13}C NMR (100 MHz, $CDCl_3$) δ 154.7, 80.0, 56.4, 44.9, 28.3; ESI-MS (m/z): $[M+Na]^+$ 224.0. Compound **3** was prepared according to the following procedures. Compound **3-4** (600 mg, 2.7 mmol) was dissolved in 20 mL of an ice-cooled, saturated solution of hydrogen chloride in ethyl acetate. The solution was kept stirring at room temperature overnight. The resulting suspension was evaporated under vacuum to give compound **3**. ^{1}H NMR (400 MHz, D_2O) δ 4.54-4.50 (m, 1H), 3.42-3.38 (m, 2H), 3.32-3.28 (m, 2H); ^{13}C NMR (100 MHz, DMSO-d_6) δ 57.2, 43.6; ESI-MS (m/z): $[M+H]^+$ 122.1.

Synthesis of compound 4. The compound was synthesized according to Supplementary Figure 82. Compound **4** was synthesized according to the literature procedure[6]. Selenium powder (3.95 g, 50 mmol) was dissolved in a mixture of 7.5 mL (150 mmol) of hydrazine hydrate and 0.92 g (15 mmol) of ethanolamine, and heated to 75 ^{o}C. The solution was heated for 2.5 h at that temperature and allowed to cool down to 40 ^{o}C. Then, 3.225 g (25 mmol) of 1,3-dichloro-2-propanol was added dropwise to the mixture. The reaction mixture was allowed to stir for 1 h at about 50 ^{o}C and cooled to room temperature, and the precipitate was separated, washed with water, ethanol, and diethyl ether, and dried under reduced pressure to obtain oligomer **4-1**. A solution of 4.2 g (75 mmol) of KOH in 18.5 mL (375 mmol) of hydrazine hydrate was heated to 60 ^{o}C, and 3.2 g (15 mmol) of oligomer **4-1** was added. The mixture was stirred for 2 h at that temperature, cooled to room temperature, and poured into a mixture of ice with 120 mL of concentrated HCl. The mixture was allowed to stir under air for 2 h and then extracted with ethyl acetate (3×50 mL). The organic phase was separated, dried

with Na_2SO_4, and evaporated under vacuum. Chromatography of the residue on silica gel with a mixture of petroleum ether/ethyl acetate (4/1) gave compound **4** as a red sticky solid (2.8 g, 87% yield). ^1H NMR (400 MHz, DMSO-d_6) δ 5.14 (d, J = 4.4 Hz, 1H), 5.08-5.03 (m, 1H), 3.26 (dd, J_1 = 10.4 Hz, J_2 = 4.0 Hz, 4H),; ^{13}C NMR (100 MHz, DMSO-d_6) δ 77.6, 38.6; EI-MS m/z (%): 218 (M^+, 26), 216 (23), 214 (14), 93 (11), 58 (100), 43 (34).

Synthesis of compound 5. The compound was synthesized according to Supplementary Figure 82. Compound **5** was prepared according to the following procedures. Selenium powder (158 mg, 2 mmol) and naphthalene (256 mg, 2 mmol) were dispersed in 10 mL of anhydrous tetrahydrofuran (THF). The resulting mixture was stirred at room temperature under argon. Freshly shaved sodium metal (46 mg, 2 mmol) was then added to the mixture under argon. The reaction mixture was allowed to stir for 2 h to enable consumption of all the sodium metal. Compound **3-2** (347 mg, 1 mmol) was dissolved in 10 mL of anhydrous THF, and the resulting solution was added dropwise to the reaction mixture under argon. After 12 h, the reaction mixture was filtered, evaporated under reduced pressure. Chromatography of the residue on silica gel with a mixture of petroleum ether/ethyl acetate (3/1) gave compound **5-1** as a red solid. Compound **5-1** (315 mg, 1.0 mmol) was dissolved in 20 mL of an ice-cooled, saturated solution of hydrogen chloride in ethyl acetate. The solution was kept stirring at room temperature overnight. The resulting suspension was evaporated under vacuum to give compound **5** (220 mg, 26% yield over two steps)[7]. ^1H NMR (400 MHz, D_2O) δ 4.94-4.91 (m, 1H), 3.42 (d, J = 4.0 Hz, 4H); ^{13}C NMR (100 MHz, DMSO-d_6) δ 59.6, 35.1; EI-MS m/z (%): 217 (M^+, 31), 215 (27), 213 (17), 123 (26), 121 (14), 56 (100), 44 (32), 43 (49), 42 (44).

Synthesis of compound 6. The compound was synthesized according to Supplementary Figure 82. This compound was synthesized according the literature procedure[8]. ^1H NMR (400 MHz, D_2O) δ 3.51 (s, 1H), 2.82-2.92 (m, 4H), 2.68 (m, 1H), 1.85 (s, 1H); ^{13}C NMR (100 MHz, DMSO-d_6) δ 50.5, 36.3, 33.8, 33.0; ESI-MS (m/z): $[M+H]^+$ 136.0.

Synthesis of compound 7. The compound was synthesized according to Supplementary Figure 82. Compound **7** was prepared according to the following procedures. Dithiothreitol (154 mg, 1 mmol) was added to a stirred solution of KOH (134 mg, 2.4 mmol) 10% w/w in H_2O in a 50 mL flask in

MeOH (25 mL) . Once dissolved, oxygen was bubbled through the reaction mixture for 24 h. The solution was then acidified to pH 5 using 1 M HCl, and extracted with ethyl acetate (3 × 50 mL), the organic layer was washed with brine (2×30 mL), dried over Na_2SO_4 and concentrated under reduced pressure to give compound **8** (135 mg, 89% yield)[9]. ^{1}H NMR (400 MHz, DMSO-d_6) δ 5.27 (d, *J* = 4.4 Hz, 2H), 3.42 (dd, J_1 = 8.8 Hz, J_2 = 3.6 Hz, 2H), 3.14-3.11 (m, 2H), 2.77-2.85 (m, 2H); ^{13}C NMR (100 MHz, DMSO-d_6) δ 73.66, 40.61; EI-MS m/z (%): 152 (M^+, 100), 134 (19.2), 108 (64.7), 87 (36.8), 70 (26.5), 64 (16.2), 47 (21.5), 45 (37.3), 44 (62.8), 43 (36.5).

Synthesis of compound 8. The compound was synthesized according to Supplementary Figure 82. This compound was synthesized according the literature procedure[7]. ^{1}H NMR (400 MHz, DMSO-d_6) δ 8.30 (s, 2H), 3.75 (s, 1H), 3.11-3.07 (m, 1H), 2.93-2.85 (m, 2H), 2.32-2.25 (m, 1H), 2.15-2.06 (m, 1H); ^{13}C NMR (100 MHz, DMSO-d_6) δ 55.3, 35.5, 25.3, 19.2; EI-MS m/z (%): 153 ($[M\text{-}Se]^+$, 8), 151 (52), 149 (24), 147 (10), 125 (10), 123 (56), 121 (27), 119 (12), 57 (24), 56 (100).

Synthesis of compound 9. The compound was synthesized according to Supplementary Figure 82. Compound **9** was prepared according to the following procedures. The aqueous sodium diselenide solution (9.5 mmol) was freshly prepared according the literature[10]. To this solution, 1,3-butadiene diepoxide (0.367 mL, 4.75 mmol) was added dropwise under argon. The mixture was refluxed for 1 h under argon and then stirred overnight at room temperature under air. The resulting mixture was extracted with ethyl acetate (10 × 50 mL), and the ethyl acetate layer was separated, dried over Na_2SO_4 and concentrated under reduced pressure. Compound **9** was obtained from purification by silica gel column chromatography (petroleum ether/ethyl acetate =1/1) as a yellow solid (540 mg, 46% yield). ^{1}H NMR (400 MHz, DMSO-d_6) δ 5.14 (d, *J* = 4.0 Hz, 2H), 3.41-3.39 (m, 2H), 3.37 (d, *J* = 6.8 Hz, 1H), 3.32 (d, *J* = 2.8 Hz, 1H), 3.06-3.01 (m, 2H); ^{13}C NMR (100 MHz, DMSO-d_6) δ 74.4, 31.6; EI-MS m/z (%): 248 (M^+, 90), 246 (82), 244 (52), 204 (40), 202 (35), 160 (83), 158 (76), 87 (80), 70 (82), 44 (56), 43 (100).

Synthesis of compound 10. The compound was synthesized according to Supplementary Figure 82. This compound was synthesized according to the literature[11]. Sulfur (1.43 g, 44.6 mmol) and $Na_2S.9H_2O$ (5.35 g, 22.3 mmol) were dissolved in 50 mL of DMF. The reaction mixture was stirred

at 80 ^{o}C for 2 h and turned to deep blue. To this solution was added pentaerythritol dibromide (5.8 g, 22.3 mmol) and heated to 120 ^{o}C. After 12 h, the reaction mixture was evaporated under reduced pressure. The residue was recrystallized with toluene to afford compound **10** as a yellow solid (1.25 g, 34% yield). ^{1}H NMR (400 MHz, DMSO-d$_6$) δ 4.85 (s, 2H), 3.41 (s, 4H), 2.90 (s, 4H); ^{13}C NMR (100 MHz, DMSO-d$_6$) δ 63.0, 58.7, 43.7; ESI-MS (m/z): [M-H]$^-$ 165.0.

General Procedure for the Synthesis of TRFS1 and TRFS2. The compounds were synthesized according to Supplementary Figure 84. Compound **11** was synthesized according to our previous publication[12]. Compound **11** (54 mg, 0.2 mmol) and DMAP (72 mg, 0.6 mmol) were dissolved in 10 mL of distilled DCM. Triphosgene (24 mg, 0.08 mmol) was added to the reaction mixture under argon and allowed to stir at room temperature. After 30 min, the correspondent cyclic disulfide (compound 2 and compound 3) was added to the mixture and stirred overnight. **TRFS1** and **TRFS2** were unstable and decomposed during purification.

General Procedure for the Synthesis of TRFS3-8. The compounds were synthesized according to Supplementary Figure 84. Compound **12** was synthesized according to our previous publication[4]. Compound **12** (54 mg, 0.2 mmol) and DMAP (72 mg, 0.6 mmol) were dissolved in 20 mL of distilled toluene. The reaction mixture was heated to 70 ^{o}C. Then triphosgene (120 mg, 0.4 mmol) was added and reflux for another 3 h. The solvent was removed under reduced pressure to afford the isocyanate (RNCO) form of compound **12**. The correspondent cyclic disulfide/diselenide (0.5 mmol) and TEA (1 mmol, 140 μL) were dissolved in 20 mL of distilled DCM. After cooling to 0 ^{o}C, the isocyanate (RNCO) in DCM (10 mL) was added dropwise. Then the reaction mixture was removed to room temperature and stirred overnight. The solvent was removed under reduced pressure and the crude product was purified by silica gel column chromatography to afford the target TRFS probes.

Characterization of TRFS3*:* a light yellow solid, 78% yield. ^{1}H NMR (400 MHz, DMSO-d$_6$) δ 9.40 (s, 1H), 8.62 (d, *J* = 8.4 Hz, 1H), 8.57 (d, *J* = 8.4 Hz, 1H), 8.51 (d, *J* = 7.2 Hz, 1H), 8.41 (d, *J* = 8.4 Hz, 1H), 7.90-7.86 (m, 1H), 7.37 (d, *J* = 8.0 Hz, 1H), 5.10-5.05 (m, 1H), 4.03 (t, *J* = 7.4 Hz, 2H), 3.37-3.31 (m, 2H), 3.24-3.21 (m, 2H), 1.64-1.56 (m, 2H), 1.37-1.30 (m, 2H), 0.92 (t, *J* = 7.4 Hz, 3H); ^{13}C NMR (100 MHz, DMSO-d$_6$) δ 164.0, 163.4, 154.1, 142.6, 132.9, 131.2, 128.9, 128.1, 126.5,

122.9, 122.3, 115.1, 114.5, 56.1, 44.9, 30.2, 20.3, 14.2; ESI-MS (m/z): $[M-H]^-$ 414.0.

Characterization of TRFS4*:* a light yellow solid, 75% yield. ^{1}H NMR (400 MHz, DMSO-d_6) δ 10.2 (s, 1H), 8.69 (d, *J* = 8.4 Hz, 1H), 8.50 (d, *J* = 7.2 Hz, 1H), 8.46 (d, *J* = 8.4 Hz, 1H), 8.16 (d, *J* = 8.0 Hz, 1H), 7.85 (t, *J* = 7.8 Hz, 1H), 5.18 (s, 1H), 4.22 (s, 2H), 4.02 (t, *J* = 6.8 Hz, 2H), 3.54 (d, *J* = 4.0 Hz, 2H), 3.11-3.04 (m, 4H), 1.61-1.58 (m, 2H), 1.37-1.31 (m, 2H), 0.92 (t, *J* = 7.2 Hz, 3H); ^{13}C NMR (100 MHz, DMSO-d_6) δ 163.9, 163.4, 154.4, 141.1, 132.1, 131.4, 129.8, 128.8, 126.8, 124.3, 122.7, 118.7, 117.5, 66.3, 63.0, 62.9, 56.8, 43.8, 30.2, 20.3, 14.2; ESI-MS (m/z): $[M-H]^-$ 459.7.

Characterization of TRFS5*:* a brownish-red solid, 54% yield. ^{1}H NMR (400 MHz, DMSO-d_6) δ 10.44 (s, 1H), 8.67 (d, *J* = 8.4 Hz, 1H), 8.51-8.46 (m, 2H), 8.12 (d, *J* = 8.4 Hz, 1H), 7.86-7.82 (m, 1 H), 6.20-6.16 (m, 1H), 4.03 (t, *J* = 7.2 Hz, 2H), 3.63-3.54 (m, 4H), 1.64-1.55 (m, 2H), 1.39-1.32 (m, 2H), 0.92 (t, *J* = 7.6 Hz, 3H); ^{13}C NMR (100 NMR, DMSO-d_6) δ 164.0, 163.4, 153.9, 141.0, 132.0, 131.4, 130.1, 128.8, 126.9, 124.8, 122.7, 119.8, 117.9, 81.4, 36.3, 30.2, 20.3, 14.3; ESI-MS (m/z): $[M-H]^-$ 510.9.

Characterization of TRFS6*:* a brownish-red solid, 75% yield. ^{1}H NMR (400 MHz, DMSO-d_6) δ 9.53 (s, 1H), 8.66 (d, *J* = 8.4 Hz, 1H), 8.58 (d, *J* = 8.4 Hz, 1H), 8.52 (d, *J* = 7.2 Hz, 1H), 8.42 (d, *J* = 7.2 Hz, 1H), 7.88 (t, *J* = 8.0 Hz, 1H), 7.23 (d, *J* = 8.0 Hz, 1H), 5.62-5.59 (m, 1H), 4.05-3.98 (m, 2H), 3.43-3.42 (m, 4H), 1.69-1.57 (m, 3H), 1.38-1.23 (m, 2H), 0.92 (t, *J* = 7.6 Hz, 3H); ^{13}C NMR (100 NMR, DMSO-d_6) δ 164.0, 163.4, 154.1, 142.7, 133.0, 131.2, 128.9, 128.3, 126.4, 122.9, 122.3, 115.0, 114.5, 58.1, 38.4, 30.2, 21.4, 20.3, 14.2, 11.9; ESI-MS (m/z): $[M-H]^-$ 509.9.

Characterization of TRFS7*:* a light yellow solid, 34% yield. ^{1}H NMR (400 MHz, DMSO-d_6) δ 10.4 (s, 1H), 8.72 (d, *J* = 8.4 Hz, 1H), 8.51 (d, *J* = 7.6 Hz, 1H), 8.48 (d, *J* = 8.4 Hz, 1H), 8.17 (d, *J* = 8.4 Hz, 1H), 7.84 (t, *J* = 7.6 Hz, 1H), 5.65 (d, *J* = 5.6 Hz, 1H), 4.83-4.77 (m, 1H), 4.05-4.01 (m, 2H), 3.75-3.68 (m, 1H), 3.40-3.21 (m, 2H), 3.05-2.94 (m, 2H), 1.64-1.57 (m, 2H), 1.37-1.34 (m, 2H), 0.92 (t, *J* = 7.6 Hz, 3H); 13C NMR δ 164.0, 163.4, 154.0, 141.2, 132.1, 131.4, 129.9, 128.8, 126.8, 124.4, 122.7, 118.9, 117.6, 76.7, 70.7, 49.1, 30.2, 29.5, 20.3, 14.2; ESI-MS (m/z): $[M-H]^-$ 445.1.

Characterization of TRFS8*:* a light yellow solid, 45% yield. ^{1}H NMR (400 MHz, DMSO-d_6) δ 9.31 (s, 1H), 8.60 (d, *J* = 8.4 Hz, 1H), 8.54-8.50 (m, 2H), 8.40 (d, *J* = 8.4 Hz, 1H), 7.89 (t, *J* = 8.0 Hz, 1H), 7.36 (brs, 1H), 5.76 (m, 1H), 4.02 (t, *J* = 7.2 Hz, 2H), 3.16-3.12 (m, 2H), 2.96-2.76 (m, 2H), 2.18 (m, 1H), 1.85 (m, 1H), 1.62-1.56 (m, 2H), 1.37-1.29 (m, 2H), 0.92 (t, *J* = 7.2 Hz, 3H); ^{13}C NMR (100 NMR, DMSO-d_6) δ 164.0, 163.4, 156.2, 153.9, 142.7, 133.0, 131.2, 128.9, 128.1, 126.5, 122.9, 122.3, 115.0, 114.5, 55.4, 38.3, 37.9, 30.2, 20.3, 14.2; ESI-MS (m/z): $[M-H]^-$ 428.0.

Synthesis of TRFS9. The compound was synthesized according to Supplementary Figure 84. Compound **13** was synthesized according to the literature[13]. Compound **13** (100 mg, 0.436 mmol) and TEA (120 μL, 0.872 mmol) were dissolved in 10 mL of distilled DCM. Triphosgene (388 mg, 1.31 mmol) was dissolved in 2 mL of distilled DCM and added to the reaction mixture dropwise at 0 ^{o}C. After 2 h, the solvent was removed under reduced pressure to afford the isocyanate (RNCO) form of compound **13**. Compound **3** (157 mg, 1 mmol) and TEA (280 μL, 2 mmol) were dissolved in 20 mL of distilled DCM and stirred at room temperature for 5 min. After cooling to 0 ^{o}C, the isocyanate (RNCO) in DCM (10 mL) was added dropwise. Then the reaction was removed to room temperature and stirred overnight. The solvent was removed under reduced pressure and the crude product was purified by silica gel column chromatography (petroleum ether/ethyl acetate = 2/1) to afford TRFS9 as a light yellow solid (118 mg, 72% yield). ^{1}H NMR (400 MHz, DMSO-d_6) δ 7.66 (m, 3H), 7.28 (s, 1H), 6.67 (s, 1H), 5.58 (d, *J* = 8.8 Hz, 1H), 5.27-5.23 (m, 1H), 3.32-3.27 (m, 2H), 3.23-3.20 (m, 2H); ^{13}C NMR (100 MHz, DMSO-d_6) δ 159.3, 155.6, 154.1, 1453, 139.8, 125.9, 123.6, 115.3, 113.4, 107.0, 104.7, 56.1, 44.6; ESI-MS (m/z): $[M-H]^-$ 374.8. HRMS-ESI (m/z): $[M+H]^+$ calcd. for 377.0163, found 377.0241.

Synthesis of TRFS10. The compound was synthesized according to Supplementary Figure 84. Compound **14** was synthesized according to the literature[14]. Compound **3** (60 mg, 0.4 mmol) and TEA (56 μL, 0.4 mmol) were dissolved in 20 mL of distilled DCM and stirred at room temperature for 5 min. Compound **14** (37 mg, 0.1 mmol), TEA (28 μL, 0.2 mmol), EDC/HCl (40 mg, 0.2 mmol) and HOBt (27 mg, 0.2 mmol) were added in order under argon and allowed to stir overnight. The solvent was removed under reduced pressure and the crude product was purified by silica gel column

chromatography (DCM/methanol=50/1) to afford TRFS10 as a red solid (35 mg, 75% yield). ^{1}H NMR (400 MHz, DMSO-d$_6$) δ 7.92 (d, *J* = 8.0 Hz, 2 H), 7.42 (d, *J* = 8.4 Hz, 2H), 6.73 (d, *J* = 8.0 Hz, 1H), 5.99 (s, 2H), 5.51 (t, *J* = 4.0 Hz, 1H), 3.39-3.35 (m, 2H), 3.31-3.28 (m, 2H), 2.56 (s, 6H), 1.36 (s, 6H); ^{13}C NMR (100 MHz, DMSO-d$_6$) δ 165.5, 155.1, 142.6, 141.0, 137.0, 134.4, 130.4, 128.3, 128.0, 121.5, 56.6, 42.9, 14.2, 14.1; ESI-MS (m/z): $[M+H]^+$ 472.3.

Synthesis of TRFS11. The compound was synthesized according to Supplementary Figure 84. Compound **3** (157 mg, 1 mmol) and TEA (280 μL, 2 mmol) were dissolved in 20 mL of distilled DCM and stirred at room temperature for 5min. Dansyl chloride (108 mg, 0.4 mmol) was added to the reaction mixture and stirred overnight. The solvent was removed under reduced pressure and the crude product was purified by silica gel column chromatography (petroleum ether/ethyl acetate = 4/1) to afford TRFS11 as a yellow solid (39 mg, 27% yield). ^{1}H NMR (400 MHz, $CDCl_3$) δ 8.58 (d, *J* = 8.8 Hz, 1H), 8.31 (d, *J* = 7.2 Hz, 1H), 8.21 (d, *J* = 8.8 Hz, 1H), 7.60 (t, *J* = 8.0 Hz, 1H), 7.55 (t, *J* = 7.6 Hz, 1H), 7.21 (d, *J* = 7.6 Hz, 1H), 5.31 (d, *J* = 10.0 Hz, 1H), 4.57-4.52 (m. 1H), 2.99-2.95 (m, 2H), 2.90 (s, 6H), 2.87-2.83 (m, 2H); ^{13}C NMR (100 MHz, $CDCl_3$) δ 152.4, 135.2, 131.3, 130.2, 130.0, 129.7, 129.0, 123.5, 118.7, 115.7, 59.1, 45.7, 45.0; ESI-MS (m/z): $[M+H]^+$ 355.0.

Synthesis of TRFS12. The compound was synthesized according to Supplementary Figure 84. Compound **15** was synthesized according to the literature[15]. Compound **15** (74 mg, 0.4 mmol) and DMAP (144 mg, 1.2 mmol) were dissolved in 20 mL of distilled toluene. The reaction mixture was heated to 70 ^{o}C. Then triphosgene (240 mg, 0.8 mmol) was added and reflux for another 3 h. The solvent was removed under reduced pressure to afford the isocyanate (RNCO) form of compound **15**. Compound **3** (157 mg, 1 mmol) and TEA (2 mmol, 140 μL) were dissolved in 20 mL of distilled DCM. After cooling to 0 ^{o}C, the isocyanate (RNCO) in DCM (10 mL) was added dropwise. Then the reaction was removed to room temperature and stirred overnight. The solvent was removed under reduced pressure and the crude product was purified by silica gel column chromatography (petroleum ether/ethyl acetate=2/1) to afford the target TRFS12 as a light yellow solid (85 mg, 66% yield). ^{1}H NMR (400 MHz, DMSO-d$_6$) δ 9.07 (s, 1H), 8.53 (s, 1H), 8.10 (d, *J* = 2.0 Hz, 1H), 8.00 (d, *J* = 8.8, 1H), 7.90 (d, *J* = 1.6 Hz, 1H), 7.88 (d, *J* = 1.6 Hz, 1H), 7.81 (d, *J* = 8.8 Hz, 1H), 7.51 (dd, J_1

= 2.0 Hz, J_2 = 8.8 Hz, 1H), 6.59 (d, J = 8.0 Hz, 1H), 5.00-4.95 (m,1H), 3.32-3.29 (m, 2H), 3.18-3.15 (m, 2H), 2.66 (s, 1H); ^{13}C NMR (100 MHz, DMSO-d_6) δ 197.9, 154.6, 140.8, 136.7, 132.6, 130.9, 130.7, 128.2, 127.6, 124.5, 120.5, 112.6, 56.2, 44.6, 27.0; ESI-MS (m/z): $[M+H]^+$ 333.3.

Synthesis of TRFS13. The compound was synthesized according to Supplementary Figure 84. Compound **16** was synthesized according to our previous publication[16]. Compound **16** (62 mg, 0.2 mmol) and DMAP (72 mg, 0.6 mmol) were dissolved in 20 mL of distilled toluene. The reaction mixture was heated to 70 ^{o}C. Then triphosgene (120 mg, 0.4 mmol) was added and reflux for another 3 h. The solvent was removed under reduced pressure to afford the isocyanate (RNCO) form of compound **16**. Compound **3** (157 mg, 1 mmol) and TEA (2 mmol, 140 μL) were dissolved in 20 mL of distilled DCM. After cooling to 0 ^{o}C, the isocyanate (RNCO) form in DCM (10 mL) was added dropwise. Then the reaction was removed to room temperature and stirred overnight. The solvent was removed under reduced pressure and the crude product was purified by silica gel column chromatography (DCM/MeOH=50/1) to afford TRFS13 as a red solid (54 mg, 59% yield). ^{1}H NMR (400 MHz, DMSO-d_6) δ 9.26 (s, 1H), 8.72 (d, J = 8.0 Hz, 1H), 7.92 (t, J = 7.6 Hz, 1H), 7.79 (d, J = 8.0 Hz, 1H), 7.71-7.65 (m, 3H), 7.60 (t, J = 7.6 Hz, 1H), 7.49 (d, J = 8.8 Hz, 2H), 7.34 (d, J = 16.0 Hz, 1H), 6.97 (s, 1H), 6.74 (d, J = 7.6 Hz, 1H), 4.92-4.90 (m, 1H), 3.32-3.28 (m, 2H), 3.14-3.10 (m, 2H);^{13}C NMR (100 MHz, DMSO-d_6) δ 159.1, 154.3, 153.3, 152.5, 143.0, 139.3, 135.8, 129.9, 128.4, 126.5, 125.1, 119.5, 118.0, 117.8, 117.6, 117.2, 116.5, 106.5, 59.8, 58.9, 56.1, 46.3, 44.6; ESI-MS (m/z): $[M-H]^-$ 456.9.

Supplementary Notes

Detailed Mechanism of TRFS-green Activation. One major unfavorable property of TRFS-green is its slow response to TrxR[4]. As shown in Figure 1A, there are two steps in the activation of TRFS-green: 1) generation of the intermediate *via* cleavage of the disulfide bond, and 2) a CDR process to liberate the aminonaphthalimide (ANA) fluorophore. We presented experimental evidence herein to demonstrate that the cyclization of the intermediate (the second step in Figure 1A) contributes to the sluggish response of the probe. TRFS-green (20 μM) was incubated with TCEP (1 mM), and the reaction process was monitored by the high-performance liquid chromatography (HPLC) with a mass detector or photo diode array (PDA) detector. The original time-dependent HPLC chromatograms were shown in Figure 2A. Quantification of the intermediate and ANA production was shown in Figure 2B. Different concentrations of ANA and TRFS-green were run on HPLC to obtain calibration curves, from which the concentrations of ANA and TRFS-green in the reaction mixture were acquired. Since the intermediate is not stable and the authentic compound cannot be obtained, we estimated its concentration by assuming that there is no side reaction during the conversion of TRFS-green to the intermediate in the first 10 min. This is reasonable as there are no other detectable peaks shortly after the probe reduction. The peak with a retention time (RT) of 30.5 min was identified as the corresponding intermediate (m/z=417.0, $[M-H]^-$). Consumption of TRFS-green was fast, and >95% of the probe disappeared within 10 min. Coincident with the disappearance of TRFS-green, the intermediate appeared as a predominant product (~90%). There was only trace amount of ANA (<5%) production in the first 10 min. The concentrations of the intermediate increased within the first 1 h, and then gradually decreased. In contrast, the concentrations of ANA kept increasing within the first 4 h. We also determined the time-dependent emission signal of the reaction mixture (Figure 2C). Consistent with the steady increase of ANA production in the first 4 h, the fluorescence signal kept increasing during the period of detection. Taken together, these results strongly support that the cyclization of the intermediate is a rate-limiting step for the activation of TRFS-green.

Screening of TRFS probes. TCEP is a strong reducing agent, and may readily reduce disulfide and

diselenide bonds. To simplify the experimental procedure, we employed TCEP in place of TrxR/NADPH for the initial screening of the probes (TRFS3-8). Incubation of TRFS3 with TCEP for 3 h gave no apparent ANA signal when the mixture excited at 438 nm (Supplementary Figure 8A). However, when the mixture was excited at 363 nm (the maximal absorbance of TRFS3), a fast increase of the emission signal centering at ~495 nm was observed, and the fluorescence intensity reaches a plateau (~10-fold increase) within 2 min (Supplementary Figure 1A). When TRFS4 was incubated with TCEP, a weak but repeatable ANA fluorescence signal appeared, and the fluorescence intensity centering at 538 nm increased constantly within 4 h giving a ~4-fold elevation of the emission signal (Supplementary Figure 1A). Compared to the TRFS-green that shows nearly an 18-fold increase of fluorescence intensity within 3 h (Figure 2C), this observation led us to conclude that the release of the fluorophore is unfavorable when a six-membered cyclization product (referring to the second step of TRFS-green in Figure 1A) was formed. TRFS5, a selenium analogue of TRFS-green, responded to TCEP to release the ANA fluorophore (Supplementary Figure 1C). It gave a >80-fold increase of the ANA emission signal within 10 min, which is much faster than the probe TRFS-green (Figure 2C). This observation indicated that replacement of the five-membered cyclic disulfide moiety with the corresponding diselenide could improve the response rate drastically. It is known that selenolates have stronger nucleophilicity than do thiolates[17, 18], which may account for the observed fast response of TRFS5. Similar to its sulfur analogue TRFS3, TRFS6 displayed a negligible ANA fluorescence signal when it was incubated with TCEP for 3 h (Supplementary Figure 8B). Also, a great increase (>100-fold) of the emission signal centering at ~495 nm was observed, and the fluorescence intensity reached a plateau within 5 min (Supplementary Figure 1D). Replacement of the recognition part in TRFS-green to the 6-membered cyclic disulfide yields the probe TRFS7. Treatment of TRFS7 with TCEP gradually liberated the ANA fluorophore (Supplementary Figure 1E). The fluorescence signal increased ~17-fold within 3 h, which is comparable to the response of TRFS-green to TCEP (Figure 2C). TRFS8, similar to the probe TRFS3 and TRFS6 that have two nitrogen atoms in the linker unit, showed marginal fluorescence signal of the ANA within 3 h (Supplementary Figure 8C), indicating the probe was incapable of releasing the fluorophore. Again, the increase (~3-fold) of the emission signal centering at ~495 was observed within 15 min when the mixture was excited at 360 nm (Supplementary Figure 1F). For clarity, the response of the probes TRFS3-8 was summarized in Table 2 (the second and third columns).

Based on these results, a preliminary structure-activity relationship (SAR) of these probes (Figure 1B & Figure 3) in responding to TCEP could be drawn. First, the linker atom directly connecting to the fluorophore (X in Figure 1B) determines the stability of the probes, and the nitrogen atom is expected to improve the stability while probes contain the oxygen atom are not stable (TRFS1 and TRFS2). Second, both the Y atom and the number of carbons between the recognition part and the Y atom (Y and n in Figure 1B) determine the release of the fluorophore. To efficiently liberate the fluorophore, Y=O and n=0 are preferred (TRFS-green, TRFS5 and TRFS7). Third, replacement of the disulfide in the recognition part with diselenide promotes the release of the fluorophore (TRFS5 & TRFS-green). As such CDR strategy (the second step of TRFS-green activation shown in Scheme 1) is widely applied in designing probes, prodrugs and theranostic agents[19, 20, 21, 22, 23, 24, 25, 26, 27, 28, 29, 30, 31], our clarification of the SAR of such type molecules would have a general interest and could advance the development of various probes, prodrugs and theranostic agents.

GSH is the most abundant biothiol in cells, and actively participates in various thiol-disulfide exchange reactions. Before we determined the response of the probes to TrxR, we examined whether GSH could activate the probes to give fluorescence signals. TRFS5 is the only one that gave a ~6-fold increment of fluorescence signal after incubation of the probe with GSH for 3 h (Supplementary Figure 2A), suggesting that the 5-membered cyclic diselenide could be reduced by GSH. Other TRFS probes showed negative response to GSH either excited at 438 nm or 363 nm (Supplementary Figure 9). We next determined whether TrxR could turn on the fluorescence of these probes. Incubation of TRFS3 with TrxR and NADPH, no significant ANA fluorescence signal appeared when excited at 438 nm, which is similar to its reduction by TCEP (Supplementary Figure 8A). However, when the reaction mixture was excited at 363 nm, a fast increase (~6-fold) of the fluorescence signal centering at ~495 nm was observed (Supplementary Figure 2B). TRFS4 gave an emission signal centering at ~475 nm with the excitation at 382 nm (Supplementary Figure 2C), but there was no apparent signal of the ANA fluorophore. This observation indicated that TrxR could reduce the disulfide bond but the following CDR process did not happen due to the unfavorable formation of a six-membered cyclization product. The fluorescence signal of TRFS5 was also switched on by TrxR, and a rough 6-fold increase of the ANA signal (λ_{ex}=438 nm) was observed within 3 h (Supplementary Figure 2D). There was no significant fluorescence increment when

incubation of TRFS6, TRFS7 or TRFS8 with TrxR and NADPH, either excited at 363 nm or 438 nm (Supplementary Figure 10), indicating that the six-membered cyclic disulfides and five-membered cyclic diselenides are likely not reduced by TrxR. Taken together, after we examined the response of the probes with GSH and TrxR, we could conclude that the recognition part containing five-membered cyclic disulfides showed selectivity for TrxR over GSH. For clarity, the response of the probes to GSH and TrxR was summarized in Table 2 (the last two columns).

Sensing Mechanism of TRFS3. An interesting observation from the reduction of TRFS3-8 by TCEP is that there are two different types of fluorescence spectra. One is the blue emission centering at ~490 nm with the excitation at 360-390 nm (Supplementary Figure 1A, D and F), and the other is the green emission centering at 538 nm with the excitation at 438 nm (Supplementary Figure 1B, C and E). The fluorescence spectra of the later one are from the released ANA. We were curious about the blue emission. In addition, TRFS3 showed marginal response to GSH. Thus the detailed reaction process of TRFS3 with TCEP was monitored by HPLC coupled with a mass or PDA detector. As expected, there was no detectable ANA production even incubation of the reaction mixture for 4 h (Figure 4A). Consumption of TRFS3 was very fast, and over 95% of the probe disappeared within 1 min. Coincident with the consumption of TRFS3, a new peak with a molecular weight (MW) of 416.0 ($[M-H]^-$) under the negative ionization mode was observed within 1 min, and this peak intensity hardly changed in the following 4 h. TRFS3 has a MW of 415.1. This new peak is believed to be from the direct reduction of TRFS3 by TCEP, which gave the product with a MW of 417.1 (Figure 4B). Due to the stability of the linker unit (-NH-C(O)-NH-), the cyclization by the nascent thiolate attack did not take place, and thus no ANA was released. The fast appearance of blue emission from the reaction mixture (Supplementary Figure 1A) also supported a direct reduction of TRFS3 by TCEP. It was reported that carbonylation of the amino group of ANA causes a blue shift of its absorbance from ~440 nm to ~370 nm. In addition, this modification also changes the green emission of the ANA (~540 nm) to a blue emission of the amide form of ANA (~470 nm)[32, 33, 34]. Taken together, these results demonstrated that a direct reduction of TRFS3 without the following cyclization (Figure 4B) occurred in the response of the probe to TCEP. Furthermore, the off-on fluorescence signal of TRFS3 (and other probes, such as TRFS6 and TRFS8) in response to TCEP also suggested that the disulfide/diselenide bond could quench the emission of certain fluorophores,

and may serve as a trigger in designing fluorescent probes.

Reduction of Cyclic Disulfides/Diselenides. Discovery of small molecule ligands of a protein of interest is critical for chemical manipulation of the protein. Disulfides and diselenides are a class of redox-active compounds with multiple biological functions. It has been well documented that many linear disulfides/diselenides are good substrates of TrxR[35, 36, 37, 38, 39]. However, studies on the interaction of cyclic disulfides with TrxR are limited[40, 41, 42, 43], and there is no study on the interaction of cyclic diselenides with TrxR. To extend this preliminary result, *i.e.*, the selective reduction of 5-membered cyclic disulfides by TrxR, we further prepared a series of cyclic disulfides/diselenides (**1**-**9**, Table 3), and studied their interactions with TrxR and GSH. The detailed synthetic procedures and characterization of these molecules were described in the Supplementary Information. It is not convenient to determine the direct reduction of the molecules by GSH. Thus, we adopted a coupled enzymatic assay with GR and NADPH[44]. The decay of absorbance at 340 nm (A_{340}), due to the oxidation of NADPH, was monitored for 10 min, and the rates of A_{340} decay within the initial 3 min were calculated and summarized in Table 3. Without the test compounds, the background decay of A_{340} in the TrxR assay is $0.54 x 10^{-4}$ s^{-1}. In the presence of compounds **1**, **2**, **3**, **5**, and **9**, the decay of A_{340} was significantly higher than the background, indicating these compounds are substrates of TrxR. Without the test compounds, the background decay of A_{340} in the GR/GSH assay is $0.70 x 10^{-4}$ s^{-1}. In the presence of compounds **4**, **5**, **8** and **9**, the oxidation of NADPH was faster than the background, indicating that GSH could reduce these compounds. Based on these data, the SAR could be drawn: 1) The 1, 2-dithianes (6-membered cyclic disulfides, compounds **6** & **7**) cannot be reduced by either TrxR or GSH; 2) The 1, 2-dithiolanes (5-membered cyclic disulfides, compounds **1**, **2** & **3** are substrates of TrxR but cannot be reduced by GSH. The reduction of the cyclic diselenides is a little bit complicated. Compounds **5** and **9** are substrates of both TrxR and GSH, while compound **8** is resistant to TrxR but appears a weak substrate of GSH. Interestingly, compound **4** seems to be selectively reduced by GSH but not by TrxR. In the comparison of 1, 2-dithiolanes reduction, the reduction of aminodithiolane (**3**) is much faster than the hydroxydithiolane (**2**) and carboxylicdithiolane (**1**). Under our assay conditions, the amino group in **3** and the carboxylic group in **1** are positively and negatively charged, respectively. The positively charged amino group would have a favorable electrostatic interaction with the negatively charged

C-terminal active site of TrxR, while the interaction between the negatively charged carboxylic group and the latter is unfavorable. This might account for the observed different reduction rates of the three dithiolanes by TrxR. Taken together, although more data are needed to obtain a clear picture of reduction of cyclic diselenides, it is evident that 1, 2-dithiolanes display promising selectivity to TrxR over GSH, which strongly supports the selective activation of TRFS3 and TRFS4 by TrxR. This discovery demonstrated that the 1, 2-dithiolane moiety may serve a general ligand in designing chemical tools to target TrxR selectively.

Theoretical Calculations. All optimized structures were shown in Supplementary Figure 3A. The Frontier molecular orbitals and corresponding energies of Fast-TRFS and R-Fast-TRFS in their ground states were shown in Supplementary Figure 3B. The UV-Vis absorption spectra of Fast-TRFS and R-Fast-TRFS were calculated based on optimized ground state structures, which are shown in Supplementary Figures 4 and 5. The calculated UV-Vis absorption peaks of Fast-TRFS and R-Fast-TRFS were very close, which is in agreement with experimental spectra, although they are both about 40 nm blue-shifted than experimental spectra.

In Supplementary Figure 4 and Supplementary Table 1, for molecule Fast-TRFS, the absorbance of $\mathbf{S^1}$ excited state can be hardly seen due to its low oscillator strength, while $\mathbf{S^2}$ excited state contributed dominantly to the absorption peak near 300 nm (corresponding to the absorption peak at 346 nm in experiment). While in Supplementary Figure 5 and Supplementary Table 2, for molecule R-Fast-TRFS, the absorbance of S^1 excited state makes major contribution to the absorption peak near 300 nm (corresponding to the absorption peak at 346 nm in experiment). Therefore, we consider that Fast-TRFS will be excited from ground state to S^2 excited state, while R-Fast-TRFS will be excited from ground state to S^1 excited state.

Then we optimized the S^2 excited state structure of Fast-TRFS, and the S^1 excited state structure of R-Fast-TRFS, their fluorescence emission spectra were calculated based on the optimized excited state structures.

From Supplementary Figure 6 and Supplementary Table 3 it can be seen that: when the molecule Fast-TRFS is in S^2 excited state structure, its S^1 emission peak has low oscillator strength (0.0011), while S^2 emission peak has high oscillator strength (0.6753). However the strong S^2 emission peak is not observed in experiment, we thought that internal conversion might occur from

S^2 to S^1 excited state. When the molecule Fast-TRFS is in S^2 excited state structure, the energy difference between S^2 and S^1 excited states are small (~0.22 eV, Supplementary Table 3), making the internal conversion possible. After internal conversion of Fast-TRFS, S^1 emission took place, leading to low intensity of fluorescence.

From Supplementary Figures 5 and 7, Supplementary Tables 2 and 4 we can draw conclusion that: for molecule R-Fast-TRFS, the UV-Vis absorption and fluorescence emission can be mainly attributed to HOMO-LUMO transition type, therefore its absorption peak and emission peak have similar oscillator strengths.

Supplementary References

1. Luo XB, Dong RZ, Luo SL, Zhan YC, Tu XM, Yang LX. Preparation of Water-Compatible Molecularly Imprinted Polymers for Caffeine with a Novel Ionic Liquid as a Functional Monomer. *J. Appl. Polymer Sci.* **127**, 2884-2890 (2013).
2. Venditti A*, et al.* Dihydroasparagusic acid: antioxidant and tyrosinase inhibitory activities and improved synthesis. *J. Agric. Food Chem.* **61**, 6848-6855 (2013).
3. Levanova E*, et al.* Selective synthesis of isomeric dithioglycerols. *Russ. J. Org. Chem.* **44**, 1428-1433 (2008).
4. Zhang L*, et al.* Highly selective off-on fluorescent probe for imaging thioredoxin reductase in living cells. *J. Am. Chem. Soc.* **136**, 226-233 (2014).
5. Thomas RC, Reed LJ. Disulfide Polymers of DL-α-Lipoic Acid. *J. Am. Chem. Soc.* **78**, 6148-6149 (1956).
6. Elaev AV*, et al.* Reactions of chloromethyloxirane and dihalopropanols with chalcogens in basic reducing systems. *Russ. J. Org. Chem.* **44**, 505-510 (2008).
7. Lukesh JC, 3rd, Vanveller B, Raines RT. Thiols and selenols as electron-relay catalysts for disulfide-bond reduction. *Angew. Chem. Int. Ed.* **52**, 12901-12904 (2013).
8. Lukesh JC, 3rd, Palte MJ, Raines RT. A potent, versatile disulfide-reducing agent from aspartic acid. *J. Am. Chem. Soc.* **134**, 4057-4059 (2012).
9. Gasparini G, Sargsyan G, Bang EK, Sakai N, Matile S. Ring Tension Applied to Thiol-Mediated Cellular Uptake. *Angew. Chem. Int. Ed.* **54**, 7328-7331 (2015).
10. Klayman DL, Griffin TS. Reaction of selenium with sodium borohydride in protic solvents. A Facile Method for the introduction of selenium into organic molecules. *J. Am. Chem. Soc.* **95**, 197-199 (1973).
11. Wessig P*, et al.* Molecular Rods Based on Oligo-spiro-thioketals. *J. Org. Chem.* **81**, 1125-1136 (2016).
12. Zhou P, Yao J, Hu G, Fang J. Naphthalimide Scaffold Provides Versatile Platform for Selective Thiol Sensing and Protein Labeling. *ACS Chem. Biol.* **11**, 1098-1105 (2016).
13. Sun Q, Li J, Liu WN, Dong QJ, Yang WC, Yang GF. Non-peptide-based fluorogenic small-molecule probe for elastase. *Anal. Chem.* **85**, 11304-11311 (2013).
14. Kolemen S*, et al.* Optimization of distyryl-Bodipy chromophores for efficient panchromatic sensitization in dye sensitized solar cells. *Chem. Sci.* **2**, 949-954 (2011).
15. Chen PZ*, et al.* Light-Harvesting Systems Based on Organic Nanocrystals To Mimic Chlorosomes. *Angew. Chem. Int. Ed.* **55**, 2759-2763 (2016).
16. Zhang L*, et al.* A specific fluorescent probe reveals compromised activity of methionine sulfoxide reductases in Parkinson's disease. *Chem. Sci.* **8**, 2966-2972 (2017).
17. Reich HJ, Hondal RJ. Why Nature Chose Selenium. *ACS Chem. Biol.* **11**, 821-841 (2016).
18. Zhang B, Ge C, Yao J, Liu Y, Xie H, Fang J. Selective selenol fluorescent probes: design, synthesis, structural determinants, and biological applications. *J. Am. Chem. Soc.* **137**, 757-769 (2015).
19. Yan C, Guo Z, Shen Y, Chen Y, Tian H, Zhu WH. Molecularly precise self-assembly of theranostic nanoprobes within a single-molecular framework for in vivo tracking of tumor-specific chemotherapy. *Chem. Sci.* **9**, 4959-4969 (2018).
20. Yan C, Guo Z, Liu Y, Shi P, Tian H, Zhu WH. A sequence-activated AND logic dual-channel fluorescent probe for tracking programmable drug release. *Chem. Sci.* **9**, 6176-6182 (2018).
21. Li X*, et al.* Selective Activation of a Prodrug by Thioredoxin Reductase Providing a Strategy to Target Cancer Cells. *Angew. Chem. Int. Ed.* **57**, 6141-6145 (2018).
22. Lee MH*, et al.* Fluorogenic reaction-based prodrug conjugates as targeted cancer theranostics. *Chem. Soc. Rev.* **47**, 28-52 (2018).

23. Kong F, *et al.* Highly Selective Fluorescent Probe for Imaging H2Se in Living Cells and in Vivo Based on the Disulfide Bond. *Anal. Chem.* **89**, 688-693 (2017).
24. Chen W, Xu S, Day JJ, Wang D, Xian M. A General Strategy for Development of Near-Infrared Fluorescent Probes for Bioimaging. *Angew. Chem. Int. Ed.* **56**, 16611-16615 (2017).
25. Ye M, *et al.* Dual-channel NIR activatable theranostic prodrug for in vivo spatiotemporal tracking thiol-triggered chemotherapy. *Chem. Sci.* **7**, 4958-4965 (2016).
26. Kong F, Liang Z, Luan D, Liu X, Xu K, Tang B. A Glutathione (GSH)-Responsive Near-Infrared (NIR) Theranostic Prodrug for Cancer Therapy and Imaging. *Anal. Chem.* **88**, 6450-6456 (2016).
27. Lee MH, Sessler JL, Kim JS. Disulfide-based multifunctional conjugates for targeted theranostic drug delivery. *Acc. Chem. Res.* **48**, 2935-2946 (2015).
28. Liu C, *et al.* Rational design and bioimaging applications of highly selective fluorescence probes for hydrogen polysulfides. *J. Am. Chem. Soc.* **136**, 7257-7260 (2014).
29. Lee MH, *et al.* Toward a chemical marker for inflammatory disease: a fluorescent probe for membrane-localized thioredoxin. *J. Am. Chem. Soc.* **136**, 8430-8437 (2014).
30. Liu C, *et al.* Capture and visualization of hydrogen sulfide by a fluorescent probe. *Angew. Chem. Int. Ed.* **50**, 10327-10329 (2011).
31. Qi R, *et al.* Nanoparticle conjugates of a highly potent toxin enhance safety and circumvent platinum resistance in ovarian cancer. *Nat. Commun.* **8**, 2166 (2017).
32. Silvers WC, Prasai B, Burk DH, Brown ML, McCarley RL. Profluorogenic reductase substrate for rapid, selective, and sensitive visualization and detection of human cancer cells that overexpress NQO1. *J. Am. Chem. Soc.* **135**, 309-314 (2013).
33. Zhang JF, Lim CS, Bhuniya S, Cho BR, Kim JS. A highly selective colorimetric and ratiometric two-photon fluorescent probe for fluoride ion detection. *Org. Lett.* **13**, 1190-1193 (2011).
34. Jiang J, *et al.* A colorimetric and ratiometric fluorescent probe for palladium. *Org. Lett.* **13**, 4922-4925 (2011).
35. Cunniff B, Snider GW, Fredette N, Hondal RJ, Heintz NH. A direct and continuous assay for the determination of thioredoxin reductase activity in cell lysates. *Anal. Biochem.* **443**, 34-40 (2013).
36. Sausen de Freitas A, *et al.* Reduction of diphenyl diselenide and analogs by mammalian thioredoxin reductase is independent of their gluthathione peroxidase-like activity: a possible novel pathway for their antioxidant activity. *Molecules* **15**, 7699-7714 (2010).
37. Zhao R, Holmgren A. A novel antioxidant mechanism of ebselen involving ebselen diselenide, a substrate of mammalian thioredoxin and thioredoxin reductase. *J. Biol. Chem.* **277**, 39456-39462 (2002).
38. Kirkpatrick DL, *et al.* Mechanisms of inhibition of the thioredoxin growth factor system by antitumor 2-imidazolyl disulfides. *Biochem. Pharmacol.* **55**, 987-994 (1998).
39. Holmgren A. Bovine thioredoxin system. Purification of thioredoxin reductase from calf liver and thymus and studies of its function in disulfide reduction. *J. Biol. Chem.* **252**, 4600-4606 (1977).
40. Tibodeau JD, Benson LM, Isham CR, Owen WG, Bible KC. The anticancer agent chaetocin is a competitive substrate and inhibitor of thioredoxin reductase. *Antioxid. Redox Signal.* **11**, 1097-1106 (2009).
41. Choi HS, Shim JS, Kim JA, Kang SW, Kwon HJ. Discovery of gliotoxin as a new small molecule targeting thioredoxin redox system. *Biochem. Biophys. Res. Commun.* **359**, 523-528 (2007).
42. Arner ES, Nordberg J, Holmgren A. Efficient reduction of lipoamide and lipoic acid by mammalian thioredoxin reductase. *Biochem. Biophys. Res. Commun.* **225**, 268-274 (1996).
43. Lothrop AP, Ruggles EL, Hondal RJ. No selenium required: reactions catalyzed by mammalian thioredoxin reductase that are independent of a selenocysteine residue. *Biochemistry* **48**, 6213-6223 (2009).

44. Tietze F. Enzymic method for quantitative determination of nanogram amounts of total and oxidized glutathione: applications to mammalian blood and other tissues. *Anal .Biochem.* **27**, 502-522 (1969).
