# Supplementary material for: A fast and specific fluorescent probe for thioredoxin reductase that works via disulphide bond cleavage
Source: Nat Commun. 2019 Jun 21;10:2745. doi: 10.1038/s41467-019-10807-8 (PMC6588570; doi:10.1038/s41467-019-10807-8)
Supplement: Supplementary file 3 — Reporting Summary [file 41467_2019_10807_MOESM3_ESM.pdf]

nature research

Corresponding author(s): Jianguo Fang
Last updated by author(s): May 23, 2019

Reporting Summary

Nature Research wishes to improve the reproducibility of the work that we publish. This form provides structure for consistency and transparency in reporting. For further information on Nature Research policies, see Authors & Referees and the Editorial Policy Checklist.

Statistics

For all statistical analyses, confirm that the following items are present in the figure legend, table legend, main text, or Methods section.

n/a	Confirmed	
☒	☐	The exact sample size (n) for each experimental group/condition, given as a discrete number and unit of measurement
☐	☒	A statement on whether measurements were taken from distinct samples or whether the same sample was measured repeatedly
☐	☒	The statistical test(s) used AND whether they are one- or two-sided<br>*Only common tests should be described solely by name; describe more complex techniques in the Methods section.*
☒	☐	A description of all covariates tested
☒	☐	A description of any assumptions or corrections, such as tests of normality and adjustment for multiple comparisons
☐	☒	A full description of the statistical parameters including central tendency (e.g. means) or other basic estimates (e.g. regression coefficient) AND variation (e.g. standard deviation) or associated estimates of uncertainty (e.g. confidence intervals)
☒	☐	For null hypothesis testing, the test statistic (e.g. F, t, r) with confidence intervals, effect sizes, degrees of freedom and P value noted<br>*Give P values as exact values whenever suitable.*
☒	☐	For Bayesian analysis, information on the choice of priors and Markov chain Monte Carlo settings
☒	☐	For hierarchical and complex designs, identification of the appropriate level for tests and full reporting of outcomes
☒	☐	Estimates of effect sizes (e.g. Cohen's d, Pearson's r), indicating how they were calculated

Our web collection on statistics for biologists contains articles on many of the points above.

Software and code

Policy information about availability of computer code

Data collection	ChemBioDraw Ultra 14.0; Origin 8.0; Microsoft Excel 2010.
Data analysis	ChemBioDraw Ultra 14.0; Origin 8.0; Microsoft Excel 2010.

For manuscripts utilizing custom algorithms or software that are central to the research but not yet described in published literature, software must be made available to editors/reviewers. We strongly encourage code deposition in a community repository (e.g. GitHub). See the Nature Research guidelines for submitting code & software for further information.

Data

Policy information about availability of data

All manuscripts must include a data availability statement. This statement should provide the following information, where applicable:

- Accession codes, unique identifiers, or web links for publicly available datasets
- A list of figures that have associated raw data
- A description of any restrictions on data availability

We confirm that all relevant data are included in the paper and/ or its supplementary information files.

Field-specific reporting

Please select the one below that is the best fit for your research. If you are not sure, read the appropriate sections before making your selection.

☒ Life sciences ☐ Behavioural & social sciences ☐ Ecological, evolutionary & environmental sciences

For a reference copy of the document with all sections, see nature.com/documents/nr-reporting-summary-flat.pdf

Life sciences study design

All studies must disclose on these points even when the disclosure is negative.

Sample size	No sample-size calculation was performed.
Data exclusions	No data were excluded from analysis.
Replication	All biological tests were performed at least 3 times from independent experiments.
Randomization	All samples were randomly allocated into experimental groups.
Blinding	We were blinded to group allocation during data collection and analysis.

Reporting for specific materials, systems and methods

We require information from authors about some types of materials, experimental systems and methods used in many studies. Here, indicate whether each material, system or method listed is relevant to your study. If you are not sure if a list item applies to your research, read the appropriate section before selecting a response.

Materials & experimental systems

n/a	Involved in the study	
☐	☒	Antibodies
☐	☒	Eukaryotic cell lines
☒	☐	Palaeontology
☒	☐	Animals and other organisms
☒	☐	Human research participants
☒	☐	Clinical data

Methods

n/a	Involved in the study	
☒	☐	ChIP-seq
☒	☐	Flow cytometry
☒	☐	MRI-based neuroimaging

Antibodies

Antibodies used	Aniti-TrxR1 antibody (sc-28321) and anti-mouse IgG-HRP (sc-2031) were purchased from Santa Cruz Biotechnology (Santa Cruz, USA)
Validation	TrxR1 Antibody (B-2) is a mouse monoclonal IgG2a (kappa light chain) provided at 200 µg/ml<br>raised against amino acids 71-340 of TrxR1 of human origin<br>recommended for detection of TrxR1 of mouse, rat and human origin by WB, IP, IF, IHC(P) and ELISA<br>available conjugated to agarose for IP; and to either phycoerythrin or FITC for IF, IHC(P) and FCM<br>also available conjugated to Alexa Fluor® 488, Alexa Fluor® 546, Alexa Fluor® 594 or Alexa Fluor® 647 for WB (RGB), IF, IHC(P) and FCM, and for use with RGB fluorescent imaging systems, such as iBright™ FL1000, FluorChem™, Typhoon, Azure and other comparable systems<br>also available conjugated to Alexa Fluor® 680 or Alexa Fluor® 790 for WB (NIR), IF and FCM; for use with Near-Infrared (NIR) detection systems, such as LI-COR®/Odyssey®, iBright™ FL1000, FluorChem™, Typhoon, Azure and other comparable systems<br>See m-IgGκ BP-HRP (mouse IgGκ binding protein-HRP), our highly recommended

Eukaryotic cell lines

Policy information about cell lines

Cell line source(s)	HeLa cells: from the Shanghai Institute of Biochemistry and Cell Biology, Chinese Academy of Sciences.
Authentication	The cell line used has been authenticated.
Mycoplasma contamination	The cell line used was not tested for Mycoplasma contamination
Commonly misidentified lines<br>(See ICLAC register)	none
